# A measurement comparability study to support instrument harmonization in the Canadian Longitudinal Study on Aging

**DOI:** 10.1093/aje/kwag137

**Published:** 2026-06-22

**Authors:** Talha Rafiq, Lauren E Griffith, Parminder Raina, Christina Wolfson, Susan A Kirkland, Cynthia Balion, Andrew P Costa, Varun Chaudhary, My Linh Duong, Danielle Glista, Philip Joseph, Maureen J MacDonald, Jack Scott, David B Hogan, Edwin van den Heuvel

**Affiliations:** Department of Health Research Methods, Evidence, and Impact, McMaster University, Hamilton, Ontario, Canada; Labarge Centre for Mobility in Aging, McMaster University, Hamilton, Ontario, Canada; McMaster Institute for Research on Aging, McMaster University, Hamilton, Ontario, Canada; Department of Health Research Methods, Evidence, and Impact, McMaster University, Hamilton, Ontario, Canada; Labarge Centre for Mobility in Aging, McMaster University, Hamilton, Ontario, Canada; McMaster Institute for Research on Aging, McMaster University, Hamilton, Ontario, Canada; Department of Health Research Methods, Evidence, and Impact, McMaster University, Hamilton, Ontario, Canada; Labarge Centre for Mobility in Aging, McMaster University, Hamilton, Ontario, Canada; McMaster Institute for Research on Aging, McMaster University, Hamilton, Ontario, Canada; Department of Medicine and Department of Epidemiology, Biostatistics and Occupational Health, McGill University, Montreal, Quebec, Canada; Department of Community Health and Epidemiology and Division of Geriatric Medicine, Dalhousie University, Halifax, Nova Scotia, Canada; Department of Pathology and Molecular Medicine, McMaster University, Hamilton, Ontario, Canada; Department of Health Research Methods, Evidence, and Impact, McMaster University, Hamilton, Ontario, Canada; Department of Surgery, McMaster University, Hamilton, Ontario, Canada; Division of Respirology, Department of Medicine, McMaster University, Hamilton, Ontario, Canada; Firestone Institute for Respiratory Health, McMaster University, Hamilton, Ontario, Canada; School of Communication Sciences and Disorders, Faculty of Health Sciences, Western University, London, Ontario, Canada; National Centre for Audiology, Western University, London, Ontario, Canada; Population Health Research Institute, Hamilton Health Sciences and McMaster University, Hamilton, Ontario, Canada; Department of Kinesiology, McMaster University, Hamilton, Ontario, Canada; National Centre for Audiology, School of Communication Sciences and Disorders, Western University, London, Ontario, Canada; Cumming School of Medicine, University of Calgary, Calgary, AB, Canada; Department of Mathematics and Computer Science, Eindhoven University of Technology, Eindhoven, The Netherlands

**Keywords:** measurement comparability, longitudinal studies, systematic differences, measurement error, conversion equations, Canadian Longitudinal Study on Aging (CLSA)

## Abstract

Longitudinal studies are essential for understanding health trajectories, but necessary upgrades to measurement instruments over time may compromise data comparability. The Canadian Longitudinal Study on Aging conducted a measurement comparability study to quantify systematic differences between instruments and derive conversion equations to correct for any differences. Overall, 50 participants aged ≥45 years (52% female) underwent repeated assessments with old and new instruments, including the blood pressure monitor, electrocardiogram (ECG), carotid ultrasound, spirometer, audiometer, tonometer, and dynamometer. Linear mixed-effects models were used to identify systematic differences. Cohen’s *d* was used to assess standardized mean difference, Bland–Altman plots evaluated agreement between instruments, and repeatability was assessed within instruments. Mixed-effects models showed no clinically meaningful difference across instruments, except for systolic blood pressure (SBP), which was on average 5.19 mmHg (95% CI, 2.64-7.74) higher using the new instrument. Cohen’s *d* demonstrated moderate systematic differences for several measures. Repeatability estimates showed good-to-excellent reliability for most measures; however, certain ECG and tonometer parameters showed moderate or poor precision. The results support the utility of conversion equations to account for systematic differences between instruments. The difference in SBP warrants the implementation of a conversion equation, whereas it is unclear for measures with moderate differences.

## Introduction

Longitudinal studies provide invaluable insights into how sociodemographic, biological, psychosocial, behavioral, and environmental factors interact over time and affect health outcomes. To preserve the continuity and validity of research findings in longitudinal studies, it is crucial to ensure accurate and reliable variable measurements over time. Long-term, longitudinal studies often require periodic updates to their instruments due to wear-and-tear that occurs with regular use, advances in technology, discontinuation of support for older equipment, and changes in methodological standards. Even when supplied by the same manufacturer, new equipment may introduce measurement differences that obscure the intra-individual changes seen with the passage of time. Measurement comparability studies (MCS) should be done when consistent instrumentation is not possible. These studies can identify systematic differences between different instruments, guide other studies where consistent equipment is not possible or feasible, and if necessary, inform the creation of conversion models to correct for differences between instruments.^1-5^

Several statistical approaches have been proposed to evaluate systematic differences between instruments used in longitudinal studies. Ordinary least squares and errors-in-variables regression techniques have been widely used,^1^^,^^3^^,^^4^ yet they result in biased estimates and underestimate the variance in measurements, especially in the presence of complex error patterns and missing data.^6^ Although, an errors-in-variables regression (eg, Deming regression) analysis resolves the bias, the assumption is that the measurement error is constant and independent of the true value.^7^ Such assumptions may not always hold as the error may not be constant and/or may depend on the magnitude of the value being measured. To our knowledge, most longitudinal studies have either not performed or published MCS. Even when done, previous MCS did not include multiple measurements per individual for each instrument, implement randomized testing order,^1-3^ and/or had extended time gaps between the measurements, which may introduce additional variability not attributable to the measurement devices.^4^ To address these challenges, repeated measurements from both instruments on the same individuals and the use of a mixed effects model are necessary to quantify within-subject variability and between-subject variability for both instruments separately and obtain a precise estimate for the differences in measurements between instruments.

The Canadian Longitudinal Study on Aging (CLSA) is a large, national, longitudinal research platform examining the factors influencing health and aging.^8^ Participants are followed every 3 years with CLSA now entering its fifth wave (fourth follow-up) of assessments. In this wave, instruments initially used for data collection, including blood pressure (BP) monitor, electrocardiogram (ECG), carotid ultrasound, spirometer, audiometer, tonometer, and dynamometer, were replaced. The primary objective of the CLSA MCS was to compare the average performance of new instruments being introduced with the older instruments across the following 3 dimensions: (1) statistical significance, (2) standardized mean difference, and (3) clinical significance of any differences found. The secondary objective was to quantitatively evaluate the intra- and inter-variability in the measures obtained from each instrument and qualitatively assess the variability attributable to factors such as age, sex, and testing order. Finally, the study accounted for the potential sources of error when deriving conversion equations to correct any systematic differences observed between the instruments.

## Methods

### Study design and setting

A single center MCS with 50 volunteers (see Appendix S1 for sample size estimation) from different age groups (45-54, 55-64, 65-74, 75-85) and sex, reflecting the CLSA baseline sampling strata, and without contraindications for any of the assessments, was conducted to compare the performance of new instruments being introduced with existing (referred to as “old”) instruments used in the CLSA. Faculty/staff from McMaster University, members of McMaster University Retirees Association, and members of the Ancaster Probus Club (a local organization for retired/semi-retired people) were invited to participate in this study.

Participants underwent assessments using established protocols at McMaster Innovation Park, Hamilton, Ontario, Canada, between August 28 and September 8, 2023. Except for spirometry, a single rater collected the data, and the testing order was randomized so that half of the participants were first assessed with the old followed by the new instrument while the other half were first assessed with the new followed by the old instrument. Given the physical effort required for spirometry, the first and second assessments were conducted at least 20 min apart. For all other instruments, measurements were obtained on the first instruments followed immediately by the second instrument. For operational reasons all participants were tested with the old spirometer first and the new spirometer second. While all participants provided written informed consent, the project was deemed to not require a full review by the local Research Ethics Board (REB).

### Instrument measures

A minimum of 3 repeated measures were obtained from each instrument, except for audiometer, where 1 measure each from the right and left ear at each of the 7 frequencies was obtained. Table 1 provides an overview of the model used for each instrument and the corresponding measurements collected. Trained research staff conducted all measurements in accordance with CLSA standard operating procedures. Detailed descriptions of each measurement instrument, including calibration and quality assurance procedures, are provided in Appendix S2.

**Table 1:** Summary of instruments and measurement in MCS^b^.

**Instrument**	**Old instrument**	**New instrument**	**Measures**
Blood pressure monitor	BpTRU BPM200 blood pressure monitor (VSM MedTech Ltd, Vancouver, Canada)	Microlife WatchBP (Home A, Microlife AG Swiss Corporation, Switzerland)	SBP (mmHg) Diastolic blood pressure (mmHg) Pulse rate (bpm)
Spirometer^a^	TruFlow Easy-One Air spirometer (NDD Medical Technologies, Switzerland)	Easy on-PC spirometer (NDD Medical Technologies, Zurich, Switzerland)	FEV1 (L) FVC (L)
Carotid ultrasound	GE VIVIDi	GE VIVID IQ	Carotid intima-media thickness [right and left] (mm)
Dynamometer	Handheld dynamometer (Tracker Freedom® Wireless Grip, JTECH Medical, Utah, USA)	Digital handheld dynamometer (Commander Echo Wireless Muscle Testing, JTECH Medical, Utah, USA)	Grip strength (kg)
Audiometer (dB HL)	Tremetrics RA300+ (Eden Prairie MN)	Tremetrics RA660 (Eden Prairie, MN)	Air-conduction thresholds for frequency at 500 Hz, 1000 Hz, 2000 Hz, 3000 Hz, 4000 Hz, 6000 Hz, and 8000 Hz
Tonometer (mmHg)	Reichert tonometer and ocular response analyzer	Reichert ocular response analyzer G3	Corneal-compensated IOP—right-eye Corneal-compensated IOP—left-eye Intraocular pressure—right-eye Intraocular pressure—left-eye Corneal hysteresis—right-eye Corneal hysteresis—left-eye
ECG	GE MAC 1600 ECG Analysis System using 10 Silver Mactrode ECG Electrode	MAC 5 Resting ECG | GE HealthCare (United States)	Ventricular rate (bpm), QRS duration (ms), QT interval (ms), QTC interval (ms), P axis (degree), R axis (degree), T axis (degree), P offset (ms), P onset (ms), Q offset (ms), Q onset (ms), and T offset (ms)

Abbreviations: ECG, electrocardiogram; FEV1, forced expiratory volume in 1 second; FVC, forced vital capacity; IOP, intraocular pressure; MCS, measurement comparability study; SBP, systolic blood pressure.

a For operational reasons, the testing order was not randomized for spirometry. All participants first underwent assessment with the old instrument and then the new instrument at the end of the study visit.

b A minimum of 3 repeated measures were obtained from each instrument, except when considering the audiometer, with 1 measure each from the right and left ear at each of the 7 frequencies.

### Statistical analyses

#### Agreement between instruments

The agreement between the average over 3 repeated measures from the old and new instrument were summarized using Bland–Altman plots.

#### ***Systematic differences between instruments (*** ${\boldsymbol{\beta}}_{\boldsymbol{M}}$ ***in mixed effects model)***

To better understand the differences between instruments, linear mixed-effects models were used to identify systematic differences in results between the 2 instruments and to estimate the individual and measurement variation of the instruments (see Appendix S3 for details). Although the focus of the MCS is on the estimation of the systematic difference between the instruments, the linear mixed model makes it possible to transform a measurement from 1 instrument into a predicted measurement for the other instrument (ie, a conversion or calibration model). Such a model would make it possible to produce measurements as if they were all measured with the same instrument (see Appendix S4). The systematic effect was corrected for age group and sex and controlled for testing order. A random intercept for participants was implemented for each instrument, applying the unstructured covariance structure. This participant-level random effect represents the underlying true value that the instruments are trying to measure or quantify (in metrology often referred to as the measurand), while the instrument-specific part of this random effect would allow the opportunity to have instrument-specific measurands to (1) identify a difference in measurands between instruments and (2) allow for temporal intra-individual variability related to the moment of measuring. Thus, 2 correlated random intercepts per individual were modeled (1 for each instrument) using an unstructured covariance matrix. The correlation would quantify the combination of intra-individual stability and how well the instruments measure the same measurand. Repeated measurements were considered within subjects, with observations nested within participant and instrument, to quantify variability that is induced by the instrument (referred to as repeatability). Conditionally on the mean of the repeated measurements for participant and instruments, the repeated measurements were assumed independent, with instrument-specific variances to be able to determine if instruments have similar repeatability (or measurement variability). For each type of measurement, we tested whether each instrument may measure a different quantity on the same individual. The instruments are not measuring the volunteer at the exact same time and the quantity that the instrument is trying to capture may have changed slightly from instrument to instrument due to small intra-individual variation. Additionally, we assumed that each instrument may have its own bias (assumed to be 0 for the old instrument) and repeatability. The systematic difference is quantified by the bias of the new instrument. We also investigated whether the instruments have the same repeatability and whether the measure has changed with instruments using information criteria.

#### Standardized mean difference between instruments

A Cohen’s *d* is used to quantify the systematic difference between instruments relative to the between-subject variability of measurements obtained with the old instrument (see Appendix S5). It helps evaluate whether a change in instruments would exceed biological variation, which may be of relevance when data from CLSA obtained with different instruments are being used in temporal analysis. Clearly, a smaller Cohen’s *d* indicates a smaller systematic difference between the 2 instruments. Further, an approximate or asymptotic CI was computed for Cohen’s *d* based on the number of participants and repeated measurements per instrument (see Appendix S5). Note that another form of Cohen’s *d* was also used to investigate the representativeness of the MCS volunteers with respect to CLSA participants (see Appendix S6). Cohen’s *d* of 0.2, 0.5, and 0.8 were considered as small, medium, and large effect sizes, respectively.^9^

#### Reliability of instruments

Repeatability of the instrument for a single observation, as a measure of performance for the instrument, is quantified as the proportion of measurement variability in the repeated observations of the participants expressed in terms of the total variability, which is the sum of the biological variability and this measurement variability (see Appendix S7). A lower repeatability indicates greater precision in measuring individuals and therefore a more reliable instrument, making it easier to detect changes between individuals with a single observation. The repeatability is directly related to an instrument-specific intraclass correlation coefficient (ICC) (see Appendix S7) that can be calculated as 100% minus the repeatability. These ICCs are provided for completeness. Additionally, an asymptotic CI for repeatability was calculated, with lower and upper 2.5% quantiles of the F-distribution with 96 and 47 degrees of freedom. A repeatability value of <10% indicates excellent measurement reliability, 10%-25% is considered good reliability, 25%-50% is considered moderate reliability, and more than 50% is considered poor reliability.^10^

#### Clinical significance

A clinically meaningful measurement difference (ie, one that would change the medical treatment of an individual) between the instruments was identified by consulting clinicians and reviewing evidence from relevant literature. Systematic differences were further evaluated using Cohen’s *d* to quantify effect sizes, aiding in the decision-making process for conversion.

#### Conversion equation

The conversion model was applied to the results of the MCS to compare the predicted measurements for the new instrument with the observed measurements from the old instrument by calculating ${R}^2$ and by checking the correctness of a straight regression line (Appendix S4). For all measurements, the conversion for a single observation from the new instrument to the old instrument is based on the conditional expectation of the value of the old instrument given the value of the new instrument on the same participant, using the mixed effects model. The resulting conversion equation can be written in simplified form as:


$$ {\hat{y}}_0={\hat{\mu}}_0+{\hat{c}}_{01}\big({y}_1-{\hat{\mu}}_0-{\hat{\beta}}_M\big), $$

where ${\hat{y}}_0$ is the predicted measurement on the old instrument or an individual, ${\hat{\mu}}_1$is the estimated mean value for all similar participants as the individual (based on sex and age group) with the old instrument, ${y}_1$is the observed measurement for the individual with the new instrument, and ${\hat{\beta}}_M$ is the estimated systematic difference between the new and old instrument. The coefficient ${\hat{c}}_{01}$ is a scaling factor. It is a complex function of the different variance components, representing biological and measurement variation, and the correlation between measurands. This equation converts an observed measurement from the new instrument to an expected value on the old instrument scale.

#### Sensitivity analysis

Logarithmic transformation of all measurements was used to accommodate possible heterogeneity in the residual variance. The fixed effect coefficient ${\beta}_M$ for the difference in instruments in the mixed effects model applied on the log-transformed data would represent the log ratio of the median values obtained with the new and old instruments. When the fixed effect is exponentiated and multiplied by 100%, the percentage change in measurements across the 2 instruments is attained. We evaluated whether this sensitivity analysis would lead to substantially different results and conclusions.

This study is reported using the STROBE guidelines (see Appendix S8). All analyses were conducted in SAS version 9.4 at an *α* value of .05 (2-sided).

## Results

A total of 50 individuals (26 [52%] female) were included in the analyses. The distribution across the 4 age groups were: 45-54 (*n* = 10, 20%), 55-64 (*n* = 13, 26%), 65-74 (*n* = 12, 24%), and 75-85 (*n* = 15, 30%). Descriptive statistics, Cohen’s *d*, and repeatability estimates for all instruments are reported in Table 2 and interpreted below.

**Table 2:** Descriptive statistics for key variables obtained from the old and new instruments.

**Characteristics**	**Mean (SD)**			**Cohen’s *d***	**Repeatability** ^**a**^	
	**Old instrument**	**New instrument**	***P* value** ^**b**^	**Effect size (95% CI)**	**Old instrument**	**New instrument**
**Blood pressure monitor**						
SBP (mmHg)	124.99 (17.56)	130.47 (17.98)	.0002	0.29 (0.11, 0.47)	10.75 (6.34, 17.64)	12.02 (7.13, 19.53)
Diastolic blood pressure (mmHg)	78.90 (11.67)	78.82 (11.03)	.8609	0.01 (-0.19, 0.21)	15.01 (9.03, 23.89)	11.72 (6.94, 19.09)
Pulse rate (bpm)	70.20 (13.31)	69.60 (12.19)	.0766	0.09 (-0.11, 0.29)	3.06 (1.74, 5.31)	6.44 (3.72, 10.90)
**Spirometer (L)**						
FEV1	2.57 (0.77)	2.63 (0.76)	.0001	0.10 (-0.18, 0.38)	3.90 (2.36, 6.14)	3.12 (1.88, 4.94)
FVC	3.45 (0.95)	3.48 (0.92)	.0059	0.09 (-0.20, 0.37)	3.08 (1.86, 4.88)	6.64 (4.06, 10.29)
**Carotid ultrasound (mm)**						
cIMT right	0.73 (0.20)	0.70 (0.19)	.0012	0.15 (-0.13, 0.44)	1.98 (1.19, 3.15)	1.47 (0.88, 2.35)
cIMT left	0.77 (0.19)	0.76 (0.18)	.0048	0.15 (-0.13, 0.44)	2.01 (1.21, 3.21)	2.23 (1.34, 3.55)
Grip strength (kg)	32.53 (11.31)	33.84 (12.37)	.0090	0.20 (-0.08, 0.48)	11.95 (7.47, 17.94)	7.64 (4.69, 11.76)
**Audiometer (dB HL)**						
Frequency 500 Hz	26.45 (13.61)	21.10 (13.99)	<.0001	0.43 (0.03, 0.84)	33.28 (22.88, 44.56)	34.52 (23.86, 45.92)
Frequency 1000 Hz	25.45 (14.21)	19.10 (16.02)	<.0001	0.49 (0.08, 0.89)	29.43 (18.98, 42.56)	26.95 (17.17, 39.60)
Frequency 2000 Hz	27.70 (19.23)	22.55 (18.40)	<.0001	0.31 (-0.09, 0.71)	18.10 (11.04, 28.19)	22.78 (14.22, 34.39)
Frequency 3000 Hz	33.45 (21.16)	28.90 (21.05)	.0007	0.28 (-0.12, 0.68)	25.77 (16.32, 38.15)	22.30 (13.89, 33.77)
Frequency 4000 Hz	35.80 (21.10)	30.35 (22.54)	<.0001	0.35 (-0.05, 0.75)	20.65 (12.76, 31.62)	15.32 (9.23, 24.33)
Frequency 6000 Hz	44.36 (22.75)	39.30 (24.93)	.0004	0.34 (-0.06, 0.74)	32.14 (21.02, 45.70)	20.06 (12.36, 30.84)
Frequency 8000 Hz	49.13 (23.98)	43.30 (27.46)	.0004	0.33 (-0.07, 0.73)	29.56 (19.08, 42.71)	23.69 (14.85, 35.55)
**Tonometer (mmHg)**						
Corneal-compensated IOP—right-eye	17.45 (3.28)	16.35 (3.73)	.0002	0.35 (0.07, 0.64)	23.54 (15.48, 33.16)	14.92 (9.44, 22.03)
Corneal-compensated IOP—left-eye	16.90 (3.39)	16.07 (3.68)	.0003	0.26 (-0.03, 0.54)	20.26 (13.13, 29.04)	13.97 (8.81, 20.74)
Intraocular pressure—right-eye	16.12 (3.85)	15.75 (4.39)	.1294	0.10 (-0.18, 0.39)	13.85 (8.72, 20.57)	8.90 (5.49, 13.60)
Intraocular pressure—left-eye	15.56 (3.84)	15.24 (4.34)	.0688	0.09 (-0.20, 0.37)	9.72 (6.02, 14.78)	8.42 (5.19, 12.90)
Corneal hysteresis—right-eye	9.58 (1.32)	10.25 (1.14)	<.0001	0.59 (0.30, 0.88)	41.61 (29.77, 53.45)	23.20 (15.23, 32.73)
Corneal hysteresis—left-eye	9.65 (1.23)	10.18 (0.95)	<.0001	0.52 (0.24, 0.81)	55.10 (42.18, 66.41)	43.22 (31.16, 55.08)
**ECG**						
Ventricular rate (bpm)	66.39 (11.31)	66.37 (11.80)	.8997	0.01 (-0.28, 0.29)	17.17 (10.97, 25.03)	12.84 (8.06, 19.19)
QRS duration (ms)	88.75 (41.07)	87.80 (12.70)	<.0001	0.63 (0.34, 0.91)	5.24 (3.18, 8.17)	6.78 (4.15, 10.50)
QT interval (ms)	406.02 (26.57)	406.23 (26.99)	.2744	0.06 (-0.22, 0.34)	8.16 (5.02, 12.52)	6.41 (3.91, 9.93)
QTC interval (ms)	428.36 (31.42)	424.32 (23.17)	.1048	0.10 (-0.18, 0.38)	26.04 (17.31, 36.19)	12.52 (7.84, 18.74)
P axis (degree)	52.92 (17.82)	52.09 (21.27)	.7771	0.04 (-0.24, 0.33)	30.21 (20.47, 41.09)	5.47 (3.33, 8.53)
R axis (degree)	20.58 (35.61)	20.18 (35.78)	.6120	0.01 (-0.27, 0.29)	1.27 (0.76, 2.03)	0.73 (0.43, 1.17)
T axis (degree)	41.44 (31.96)	41.65 (32.66)	.2349	0.03 (-0.25, 0.32)	5.36 (3.26, 8.35)	1.36 (0.81, 2.18)
P offset (ms)	182.02 (18.21)	180.12 (40.52)	<.0001	0.16 (-0.13, 0.44)	2.83 (1.70, 4.49)	1.24 (0.74, 1.98)
P onset (ms)	136.32 (16.02)	127.57 (31.20)	<.0001	0.16 (-0.13, 0.44)	2.94 (1.77, 4.66)	1.97 (1.18, 3.14)
Q offset (ms)	257.63 (4.60)	260.53 (5.31)	<.0001	0.79 (0.50, 1.09)	43.34 (31.26, 55.20)	21.51 (14.01, 30.63)
Q onset (ms)	217.00 (4.99)	217.29 (6.03)	.0951	0.15 (-0.14, 0.43)	36.70 (25.64, 48.30)	28.47 (19.14, 39.07)
T offset (ms)	417.03 (23.57)	421.31 (13.46)	.6705	0.03 (-0.25, 0.31)	14.67 (9.27, 21.69)	14.16 (8.93, 21.00)

Abbreviations: ECG, Electrocardiogram; FEV1, forced expiratory volume in 1 second; FVC, forced vital capacity; IOP, intraocular pressure; SBP, systolic blood pressure.

a Repeatability represents the proportion of variability of the instrument expressed in terms of the total variability: < 10% excellent, 10%-25% good, 25%-50% moderate, and > 50% poor.

b Statistical significance based on *P* value obtained from mixed effects models using *α* = .05, 2-tail test.

### Agreement between instruments using Bland–Altman

Agreement between the average of 3 repeated measurements from the old and new instruments was summarized using Bland–Altman plots (Figures S1-S7; Table S1). Most data points fell within the 95% limits of agreement, but we also saw some outliers, indicating that incidental larger differences between the 2 instruments on individuals may occur. For instance, a large difference in diastolic blood pressure (approximately −15 mmHg) was observed on individual 46 (male; older than 75 year). The potential outliers were also observed in the residual plots from the mixed-effects models, and as part of a sensitivity analysis, we excluded these outliers and found similar results overall. We did not discard the outliers in reporting of the results so as to not underestimate the performance of the instruments.

From the Bland–Altman plot, we may also see a systematic difference between instruments. For example, the Bland–Altman plot for systolic blood pressure (SBP) showed a mean difference of 5.48 mmHg with 95% limits of agreement between −13.43 and 24.40 mmHg. However, this systematic difference is better studied with the mixed effects model due to the correction of individual characteristics.

### Systematic differences between instruments and biological variability

Systematic differences in results between the old and new instruments can be found in Tables S2-S8. The systematic difference was statistically different from zero for SBP (*P* < .001), QRS duration (*P* < .001), P-offset (*P* < .001), P-onset (*P* < .001), Q-offset (*P* < .001), right (*P* < .001) and left cIMT (*P* < .001), FEV1 (*P* < .001), FVC (*P* < .001), all frequencies of the audiometer (*P* < .001), left-eye (*P* < .001) and right-eye corneal compensated IOP (*P* < .001), left-eye (*P* < .001) and right-eye corneal hysteresis (*P* < .001), and grip strength (*P* = .009). The correlation coefficient between the measurands of the instruments for all measures ranged between 0.91 and 1.0, except for the ECG parameter P-axis (*ρ* = 0.68). This indicates that both instruments measure the same quantity on individuals for all measurements, except possibly for the ECG parameters p-axis. The biological variability between individuals was similar for most of the measurements when measured with the new and old instruments, except for P-axis, 4000 and 6000 Hz, where the biological variability was larger with the new instruments (indicating that the new instruments may have less measurement noise) and for left-eye corneal hysteresis where the old instrument showed larger biological variation (Tables S2-S8). The variability attributable to factors such as age, sex, and testing order was qualitatively assessed and no notable differences were observed across these factors. The normality of model residuals was evaluated using conditional Pearson residuals, which were approximately normally distributed across all measures, with no substantial deviations observed (Figures S8-S14).

### Standardized mean differences using Cohen’s d

Although we may have shown statistically significant systematic differences between the instruments, Cohen’s *d* would quantify standardized mean differences between the instruments relative to the between-subject variability measured by the old instrument, which puts the systematic differences in perspective of biological variability. Moderate differences were observed for SBP (*d: 0.29)*, ECG parameters (QRS duration (*d: 0.63*) and Q-offset (*d: 0.79*)), tonometer measures (right and left eye IOPcc (*d: 0.35 and 0.26)* and corneal hysteresis (*d: 0.59 and 0.52*)), grip strength (*d: 0.20*)*,* and audiometer measures at all frequencies (*d: 0.28-0.49)* (Table 2)*.* Small differences were observed for all other measures from the BP monitor, ECG, and tonometer as well as measures of carotid ultrasound and spirometer. Thus, none of the systematic differences were considered biologically relevant.

Additionally, Cohen’s *d* was also used to understand the representativeness of the participants in the measurement analysis study and participants of CLSA. This analysis indicated a small to moderate effect for the difference in measures overall and stratified by age groups between the MCS and the CLSA (Tables S9-S15), suggesting that the estimates obtained in the MCS are comparable to those in the CLSA.

### Reliability of instruments (repeatability for a single measurement)

Repeatability and ICCs were calculated using variance components from the mixed-effects models. Repeatability values indicated that both BP instruments have good-to-excellent reliability, as did most ECG parameters across both instruments (Table 2). Moderate repeatability was observed for Q-onset (both instruments), Q-offset and P-axis (older instrument only). Carotid ultrasound and spirometer measures demonstrated excellent and comparable precision, while audiometer measurements varied from moderate to good depending on frequency. Regarding tonometry, both IOPcc and IOP showed good and excellent repeatability, respectively on both tonometers. However, poor repeatability was observed in the measurement of corneal hysteresis in both eyes on the old tonometer and in the left eye on the new tonometer, with moderate precision in the right eye on the new instrument. Lastly, the grip strength demonstrated excellent precision with the new dynamometer and good precision with the old dynamometer. ICCs were consistent with repeatability findings, indicating that the instrument variability of repeated measuring is for most measurement good to excellent (Table S16).

### Clinical significance of instrument differences according to experts

With one exception, the performance of the new and old instruments was found to be comparable, with observed differences between the instruments deemed not clinically meaningful based on available evidence^11-14^ and expert consultations. The only notable exception was SBP, which showed a systematic difference of 5.19 (95% CI, 2.64-7.74) mmHg with higher values on the new BP monitor compared to the old BP monitor, corresponding to a 4.18% difference (statistically significant and moderately different according to Cohen’s *d*). This difference exceeds the clinical significance threshold of 5-10 mmHg.^15^

### Conversion equations

**Figure 1:**
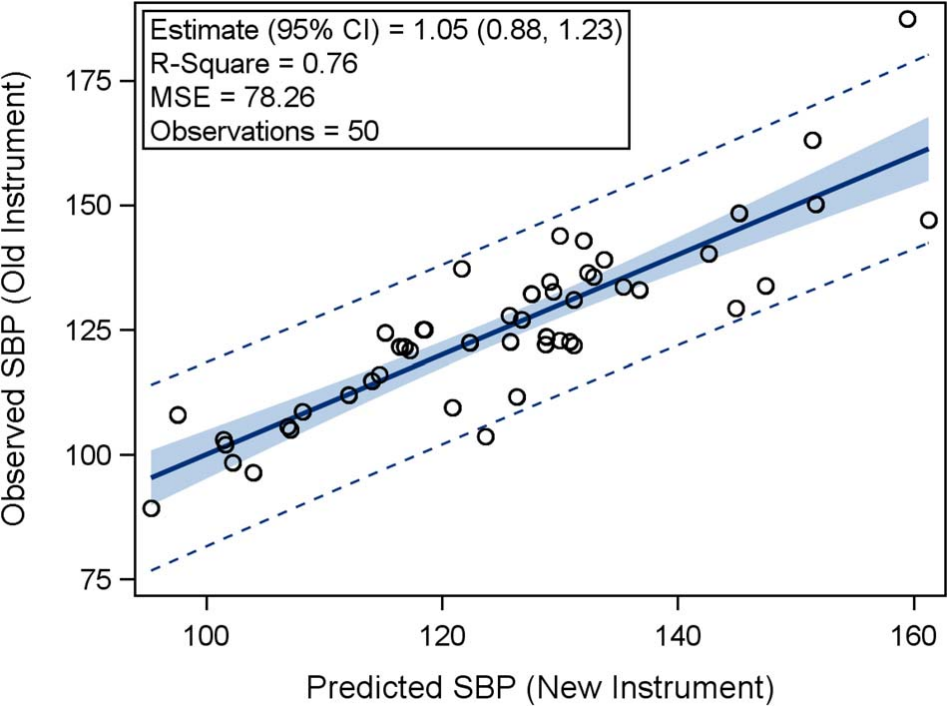
Fit plot for cross-calibration of SBP. Abbreviation: SBP, systolic blood pressure.

The conversion equations, to predict measurements for the old instrument from the new instrument, were reported and visualized in Figures S15-S21. The *R*^2^ ranges from 0.41 the left-eye corneal hysteresis to 0.99 for FEV1. Many measurements have a good prediction performance with an *R*^2^ above 0.8, except for SBP (0.76) (Figure 1), P-axis (0.74), Q-offset (0.75), Q-onset (0.78), hearing at 500 Hz (0.73), left-eye corneal compensation IOP (0.72), and right-eye (0.58) and left-eye corneal hysteresis (0.41). Conversion equations for ventricular rate (0.98), QT (0.92) and QTC interval (0.90), R (0.97) and T-axis (0.96), P onset (0.93), T offset (0.90), right (0.94) and left cIMT (0.92), FEV1 (0.99), FVC (0.98), hearing at frequencies 3000 (0.91) and 4000 Hz (0.91), left-eye intraocular pressure (0.92), and grip strength (0.93) all showed an *R*^2^ of more than 0.9, indicating excellent conversions.

The conversion equations for all measured variables across instruments are provided in Table S17. To illustrate how the conversion equation works, we focus on the linear conversion equation for SBP: ${\hat{y}}_0=120.94+0.80\times \left({y}_1-126.13\right).$For a participant with an SBP of 140 mmHg measured using the new instrument, the predicted SBP on the old instrument scale is: $132.8\approx 120.94+0.80\times \left(140-126.13.\right)$

### Sensitivity analyses

Results from models fitted on the log-transformed observations were consistent with those from the original scale, with similar estimates of systematic instrument differences.

## Discussion

We conducted a comprehensive comparison of the performance of the old and new instruments including the BP monitor, ECG, carotid ultrasound, spirometer, audiometer, tonometer, and dynamometer in the CLSA. Although most instruments showed a statistically significant systematic difference, the results (Cohen’s *d*) showed small-to-moderate systematic differences in relation to biological variability in all instruments. The most notable difference was observed for SBP, where the new BP monitor showed a clinically meaningful difference of 5.19 mmHg. The remaining instruments demonstrated good-to-excellent repeatability, with no clinically meaningful discrepancies. Conversion equations were derived to correct for systematic differences, ensuring greater accuracy and consistency in measurements across the instruments in conversion is needed for longitudinal analysis of CLSA data.

While not clinically meaningful, the statistical differences observed for several measurements between the old and new instruments were expected due to technological factors such as advancements in the manufacturers’ calibration process, computing algorithms, and/or detection technologies. The assessment of instrument repeatability showed either a comparable or improved performance of the newer instruments in comparison to the old instruments. With the exception of the BP monitor, all other instruments were sourced from the same manufacturer. The high correlation coefficients between the true values the instruments are measuring, provided evidence that the 2 instruments are indeed measuring the same quantity.

The observed difference in SBP exceeded the established minimal clinically important difference of 5-10 mmHg.^15^ This clinical difference between the Microlife WatchBP and the BpTRU BPM200 may reflect the inherent difference in the design and/or algorithm of the BP monitors. Each manufacturer uses proprietary algorithms against specific reference populations to determine the level of cuff inflation mechanics, which can result in differences in SBP.^16^ Additionally, differences in cuff design and measurement protocols may further contribute to the observed difference between the instruments. The Bland–Altman plot illustrated that the mean difference in SBP between the instruments were more pronounced at higher values, but the residuals of the mixed effects model did not clearly confirm heteroscedasticity. This supports the use of the random effects model, which corrects for participant characteristics and controls for the testing order and use instrument specific variance components, to obtain proper systematic differences between instruments and to provide conversion equations.

It may be so clear for all measurements when the conversion equations should be applied. For instance, we observed that grip strength was 1.31 kg higher on the new dynamometer, which is more than the average change of approximately -0.50 kg for men and -0.30 kg for women seen in grip strength per year among individuals over 50 years of age.^17^ So, for aging research the use of the conversion equation with excellent prediction power may be valid for longitudinal data analysis to eliminate a systematic shift in grip strengths that is larger than what is normally seen annually, but this may be less clear for all other measurements. Either for lack of information on annual change or because the predictive power of the conversion equation is insufficient. Based on our results, we recommend that future population-based studies derive their own study-specific conversion equations to account for variations in measurements across populations.

### Strengths and limitations

The present study has several strengths. The sample reflects the demographics of the CLSA, making the derived conversion equations appropriate and relevant for our research context. The study had sufficient power to detect systematic differences within 25% of repeatability, including several instruments, ensuring broad applicability for research and clinical practice, and implemented randomized testing order to reduce bias from sequence effects. Nevertheless, although multiple measurements were obtained for all parameters, only 1 measurement per ear was obtained at each frequency for the audiometer, and the values from each ear were treated as repeated measures in the analysis. While the testing order was randomized to minimize any potential order effects, logistical constraints resulted in all participants completing the spirometry assessment using the old device before the new device.

## Conclusion

Overall, the findings suggest that the performance of new and old instruments is comparable and the observed differences between new and old instruments were not deemed clinically meaningful, except for SBP. This indicates that a conversion equation is necessary for SBP to correct for differences in instruments when conducting longitudinal analyses. For instruments with a small systematic difference, no conversion equation is most likely required to correct for differences in instruments (if any) during longitudinal data analyses. Nevertheless, we have developed conversion equations to calibrate and adjust for any differences observed between new and old instruments to help researchers in their data analyses.

## Supplementary Material

Web_Material_kwag137

## Data Availability

Summary data from this study and statistical code will be made available upon request.
