# Supplementary material for: A measurement comparability study to support instrument harmonization in the Canadian Longitudinal Study on Aging
Source: Am J Epidemiol. 2026 Jun 22;195(9):2391–8. doi: 10.1093/aje/kwag137 (PMC13537845; doi:10.1093/aje/kwag137)
Supplement: Supplementary file 1 [file web_material_kwag137.pdf]

Supplemental Materials

A Measurement Comparability Study to Support Instrument Harmonization in the Canadian Longitudinal Study on Aging

Authors: Talha Rafiq; Lauren E. Griffith; Parminder Raina; Christina Wolfson; Susan A. Kirkland; Cynthia Balion; Andrew P Costa; Varun Chaudhary; MyLinh Duong; Danielle Glista; Philip Joseph; Maureen J MacDonald; Jack Scott; David B. Hogan; Edwin van den Heuvel

Table of Contents

Appendix S1: Sample size estimation 6

Appendix S2: Instruments and Procedures 6

Appendix S3: Systematic differences between instruments 8

Appendix S4: Calibration Model 9

Appendix S5: Standardized mean difference between instruments 10

Appendix S6: Comparison of MCS and CLSA participants 10

Appendix S7: Repeatability for a single observation of the instrument 10

Appendix S8: STROBE Statement—Checklist of items that should be included in reports of *cross-sectional studies* 12

Figure S1A-B: Bland-Altman plot comparing systolic and diastolic blood pressure 15

Figure S1C: Bland-Altman plot comparing pulse rate 15

Figure S2A-B: Bland-Altman plot comparing ventricular rate and QRS duration 16

Figure S2C-D: Bland-Altman plot comparing QT and QTC interval 16

Figure S2E-F: Bland-Altman plot comparing P and R axis 17

Figure S2G-H: Bland-Altman plot comparing T axis and P offset 17

Figure S2I-J: Bland-Altman plot comparing P onset and Q offset 18

Figure S2K-L: Bland-Altman plot comparing Q onset and T offset 18

Figure S3A-B: Bland-Altman plot comparing cIMT (right and left) 19

Figure S4A-B: Bland-Altman plot comparing FEV1 and FVC (using highest value) 19

Figure S5A-B: Bland-Altman plot comparing at decibels hearing level (dB HL) at Frequency 500 and 1000 Hz 20

Figure S5C-D: Bland-Altman plot comparing at decibels hearing level (dB HL) at Frequency 2000 and 3000 Hz 20

Figure S5E-F: Bland-Altman plot comparing at decibels hearing level (dB HL) at Frequency 4000 and 6000 Hz 21

Figure S5G: Bland-Altman plot comparing at decibels hearing level (dB HL) at Frequency 8000 Hz 21

Figure S6A-B: Bland-Altman plot comparing corneal compensated IOP (right and left eye) 22

Figure S6C-D: Bland-Altman plot comparing intraocular pressure (right and left eye) 22

Figure S6E-F: Bland-Altman plot comparing corneal hysteresis (right and left eye) 23

Figure S7: Bland-Altman plot comparing grip strength 23

Table S1: Bland-Altman agreement for key variables between old and new instruments 24

Table S2A: Covariance Parameter Estimates for Variability Analysis in SBP, DBP, and Pulse Rate 25
Table S2B: Mixed effects models comparing SBP, DBP, and Pulse Rate between the old and new instruments 25
Table S2C: Mixed effects models comparing log transformed SBP, DBP, and Pulse Rate between old and new instruments 26
Table S3A: Covariance Parameter Estimates for Variability Analysis in ECG measures 27
Table S3B: Mixed effects models comparing ECG measures between the old and new instruments 28
Table S3C: Mixed effects models comparing log transformed ECG measures between the old and new instruments 30
Table S4A: Covariance Parameter Estimates for Variability Analysis in carotid intima-media thickness 32
Table S4B: Mixed effects models comparing carotid intima-media thickness between the old and new instruments 32
Table S4C: Mixed effects models comparing log transformed carotid intima-media thickness between the old and new instruments 33
Table S5A: Covariance Parameter Estimates for Variability Analysis in spirometer measures .. 33
Table S5B: Mixed effects models comparing FEV1 and FVC between the old and new instruments 34
Table S5C: Mixed effects models comparing log transformed FEV1 and FVC between the old and new instruments 34
Table S6A: Covariance Parameter Estimates for Variability Analysis in audiometer measures . 35
Table S6B: Mixed effects models comparing audiometer measures between the old and new instruments 36
Table S6C: Mixed effects models comparing log transformed audiometer measures between the old and new instruments 37
Table S7A: Covariance Parameter Estimates for Variability Analysis in tonometer measures ... 39
Table S7B: Mixed effects models comparing tonometry measures between the old and new instruments 40
Table S7C: Mixed effects models comparing log transformed tonometry measures between the old and new instruments 41
Table S8A: Covariance Parameter Estimates for Variability Analysis 42
Table S8B: Mixed effects model comparing grip strength between the old and new instruments 42

Table S8C: Mixed effects model comparing log transformed grip strength between the old and new instruments 43

Figures S8: Diagnostic plots of Pearson residuals for the mixed model of SBP, DBP, and pulse rate........ 44

Figure S9: Diagnostic plots of Pearson residuals for the mixed model of grip strength 44

Figures S10: Diagnostic plots of Pearson residuals for the mixed model of audiometer measures 46

Figures S11: Diagnostic plots of Pearson residuals for the mixed model of spirometer measures 46

Figures S12: Diagnostic plots of Pearson residuals for the mixed model of cIMT measures 47

Figures S13: Diagnostic plots of Pearson residuals for the mixed model of tonometer measures48

Figures S14: Diagnostic plots of Pearson residuals for the mixed model of ECG measures 50

Table S9A: Effect Size (Cohen's d) of the difference in SBP, DBP, and Pulse Rate measures using the old instrument between Calibration study and CLSA........ 51

Table S9B: Effect Size (Cohen's d) of the difference in SBP, DBP, and Pulse Rate measures using the old instrument between Calibration study and CLSA by age 51

Table S10A: Effect Size (Cohen's d) of the difference in ECG parameters using the old instrument between Calibration study and CLSA 52

Table S10B: Effect Size (Cohen's d) of the difference in ECG parameters using the old instrument between Calibration study and CLSA by age........ 52

Table S11: Effect Size (Cohen's d) of the difference in cIMT using the old instrument between Calibration study and CLSA overall and by age 54

Table S12: Effect Size (Cohen's d) of the difference in FEV1 and FVC using the old instrument between Calibration study and CLSA overall and by age 54

Table S13A: Effect Size (Cohen's d) of the difference in audiometer measures between Calibration study and CLSA using the old instrument 55

Table S13B: Effect Size (Cohen's d) of the difference in audiometer measures between Calibration study and CLSA using the old instrument by age........ 55

Table S14A: Effect Size (Cohen's d) of the difference in tonometer measures using the old instrument between Calibration study and CLSA 56

Table S14B: Effect Size (Cohen's d) of the difference in tonometer measures using the old instrument between Calibration study and CLSA by age........ 56

Table S15: Effect Size (Cohen's d) of the difference in grip strength using the old instrument between Calibration study and CLSA overall and by age 57

Table S16: Intraclass correlation coefficient (ICC) for key variables obtained from the old and new instruments 58

Figure S15: Fit Plots for Cross-calibration of SBP, DBP, and Pulse Rate 59
Figure S16A: Fit Plots for Cross-calibration of ECG measures 60
Figure S16B: Fit Plots for Cross-calibration of ECG measures 61
Figure S17: Fit Plots for Cross-calibration of cIMT... 62
Figure S18: Fit Plots for Cross-calibration of FEV1 and FVC.. 62
Figure S19A: Fit Plot for Cross-calibration of Hearing Assessment measures............................ 63
Figure S19B: Fit Plot for Cross-calibration of Hearing Assessment measures............................ 64
Figure S20: Fit Plot for Cross-calibration of Tonometry measures... 65
Figure S21: Fit Plot for Cross-calibration of Grip Strength 66
Table S17: Conversion equation for key variables between old and new instruments 67

Appendix S1: Sample size estimation

It was expected that the measures obtained from the new instruments would be very similar to the old instruments. Therefore, the sample size (number of participants m and repeats n) was calculated to demonstrate equivalence between the two instruments, where the equivalence margin was expressed in terms of the measurement variability of a single reading (i.e., repeatability). For example, if we wish to demonstrate that the true difference in mean value of the instruments in the MCS is within 25% of the repeatability, we need at least $m = 72$ individuals and $n = 2$ repeats, $m = 49$ individuals with $n = 3$ repeats, or $m = 30$ individuals with $n = 5$ repeats. Since the standard data collection for most equipment includes at least 3 repeats, our target was to recruit at least 48 participants, with six participants from each of the eight sex and age-group strata used in the CLSA sampling. This approach ensures a balanced representation of participants across the CLSA target population. The calculations were based on a two-way ANOVA model making use of (non-central) F-statistics.

Appendix S2: Instruments and Procedures

Blood Pressure: Prior to the instrument update, systolic and diastolic blood pressure and pulse rate were measured six times while the participant was seated using a fully automated BpTRU BPM200 Blood Pressure Monitor (VSM MedTech Ltd, Vancouver, Canada). These same measurements were also obtained six times using the new Microlife WatchBP (Home A, Microlife AG Swiss Corporation, Switzerland), automatic oscillometric blood pressure monitor. For both sets of measurements, we discarded the first reading and used the subsequent five readings for analysis.

Electrocardiogram (ECG): Standard resting 12-lead ECGs were performed using the old (MAC 1600 System, GE Healthcare) and the new (MAC5, GE Healthcare) instrument. We obtained three measurements for each of the ECG parameters including Ventricular Rate, QRS Duration, QT Interval, QTC Interval, P Axis, R Axis, T Axis, P offset, P onset, Q offset, Q onset, and T offset.

Carotid doppler ultrasound: The cIMT images were obtained using a 12 MHz linear array probe attached to the old (GE VIVIDi) and the new (VIVID IQ 025) high-resolution B-mode and Doppler ultrasonography. Participants were lying in a supine position with a slightly extended neck and head turned 45° towards the opposite side. We obtained three measurements on the

posterior wall of the right and left common carotid artery. The images were analyzed using the automated edge-detection software within the ultrasound system. All measurements were obtained by a trained researcher following standard operating procedures.

Spirometer: Forced expiratory volume in the first second (FEV1) and forced vital capacity (FVC) was measured using the old TruFlow Easy-On Spirometer (NDD Medical Technologies, Zurich, Switzerland) and the new Easy on-PC spirometer (NDD Medical Technologies, Zurich, Switzerland) following the ATS/ERS guidelines. It is recommended to perform annual quality verification of volume calibration syringes to ensure the accuracy of spirometry tests, which in our study were last calibrated in October 2021. All measurements were obtained following a standardized protocol with the participant seated with a nose-clip. Eight measures for both FEV1 and FVC were obtained, and only those that met the following acceptability criteria on the instrument were retained: 90% for at least 3 measures on the old spirometer and 86% for the new spirometer. The order of testing was not randomized for spirometry as all participants first underwent assessment with the old system and then the new system at the end of study visit.

Audiometer: Hearing detection thresholds were measured using both the old instrument, a Tremetrics RA300+ (Eden Prairie, MN), and the new instrument, a Tremetrics RA660 (Eden Prairie, MN), with a digital screening audiometer and Audiocup headphone. Both instruments were calibrated in accordance with manufacturers' specifications (current [December 2022], new [July 2023]). A trained CLSA technician conducted each test in a noise-free room. According to the CLSA protocol, unaided pure-tone thresholds were assessed for each ear at frequencies of 500, 1000, 2000, 4000, 6000, and 8000 Hz. Additional details on the automated testing procedure are provided in the operations manual of the audiometer (Tremetrics RA300 and RA300+ Operations Manual 2011). The RA660 audiometer from Levitt Safety will replace the old audiometer for the fifth wave (and onwards) of CLSA data collection. We obtained one measure each from the right and left ear at each of the six frequencies listed above. The two values at each frequency were assessed as repeated measures in the analysis.

Tonometer: Paired measures of intraocular pressure were performed with the old Reichert Tonometer and Ocular Response Analyzer (TRC-NW8) and the new Reichert Ocular Response Analyzer G3 non-mydriatic retinal camera to measure intraocular pressure (IOP), corneal compensated intraocular pressure (IOPcc), and corneal hysteresis. Single field full colour fundus

disc- and macular centred images for each eye were acquired with a 45° field-of-view. Each of the tonometers were calibrated in accordance with the manufacturer's guidelines prior to the use in this study.

Dynamometer: Handgrip strength was measured using the old hand-held dynamometer (Tracker Freedom® Wireless Grip, JTECH Medical, Utah, USA) and new digital handheld dynamometer (Commander Echo Wireless Muscle Testing, JTECH Medical, Utah, USA). Handgrip was measured (in kilograms) on the dominant hand while the participants were seated with their feet flat on the floor, elbow unsupported and flexed at 90 degrees, and their forearm and wrist in a neutral position. Participants were instructed to squeeze the dynamometer handle as hard as they could three times with 15 seconds rest between trials. Three trials were performed and were included as repeated measures in the analysis. A zero-calibration check was performed every 24 hours to ensure no pressure was present on the grip gauge. Furthermore, the device underwent annual weight calibration and maintenance, conducted off-site by the manufacturer (JTECH) during the annual scheduled (December) downtime period. If the gauge readings varied by more than 5% from previous calibrations or if the device was dropped, additional recalibration was performed immediately to maintain measurement precision.

Appendix S3: Systematic differences between instruments

(1) $$y_{ijk} = \mu + Z_{ij} + \beta_M m_j + \beta_O o_i + \beta_S s_i + \sum_{r=1}^{p} \beta_{A_r} a_{r,i} + e_{ijk},$$

with y_{ijk} the kth measurement with instrument j $(= 0{,}1)$ on participant i and

- μ the mean value for women in the oldest age group who were measured with the old instrument first,
- β_M the systematic difference between the new and old instrument,
- m_j an indicator variable being equal to one when the measurement was obtained with the new instrument ($j = 1$) and zero otherwise,
- β_O the systematic difference between the order of measurement,
- o_i an indicator variable being equal to one when the new instrument was first applied to participants and zero otherwise,
- β_S the systematic difference between men and women,
- s_i an indicator variable being equal to one when the participant was male and zero

otherwise,

- β_{A_r} the systematic difference between the rth age group and the oldest age group,
- $a_{r,i}$ an indicator variable being equal to one when the participant has an age in age group r and zero otherwise
- (Z_{i0}, Z_{i1}) bivariate latent variables indicating the true value or measurand of participant i obtained with the old and new instrument.
- e_{ijk} the residual

We assumed that the latent variables (Z_{i0}, Z_{i1}) were normally distributed with mean zero and variance-covariance matrix given by $\Sigma = \begin{pmatrix} \sigma_0^2 & \rho\sigma_0\sigma_1 \\ \rho\sigma_0\sigma_1 & \sigma_1^2 \end{pmatrix}$

In case the correlation coefficient ρ is away from one ($\rho < 1$), either the two instruments do not measure the same measurand or the measurand of participants vary with time. Thus, when $\rho = 1$ and the variance components are equal ($\sigma_0^2 = \sigma_1^2$), the two instruments measure the exact same measurand. Furthermore, we assumed that the residuals are independent and normally distributed with variance τ_j^2. In case both instruments have the same repeatability (measurement variation), the variance components τ_0^2 and τ_1^2 are equal.

We investigated the general model in (1) with the model where $\tau_0^2 = \tau_1^2$ was assumed and with the model where $\tau_0^2 = \tau_1^2$, $\sigma_0^2 = \sigma_1^2$, and $\rho = 1$ was assumed. We used the AICC (the corrected Akaike Information Criterion) and BIC (the Bayesian Information Criterion) for model comparisons. Based on these metrics, the full model was generally the most suitable model for each outcome. Hence, we used the full model for all measures across all instruments.

Appendix S4: Calibration Model

Using normal distribution theory, the calibration model for a single measurement with the old instrument that is predicted by the measurement of the new instrument is given by

(2) $\hat{y}_{i0} = \hat{\mu}_i + \hat{c}_{01}(y_{i1} - \hat{\mu}_i - \hat{\beta}_M),$

with $\hat{\mu}_i = \hat{\mu} + \hat{\beta}_M m_j + \hat{\beta}_O o_i + \hat{\beta}_S s_i + \sum_{r=1}^{p} \hat{\beta}_{A_r} a_{r,i}$, $\hat{c}_{01} = \tilde{\rho}[(\hat{\sigma}_0^2 + \hat{\tau}_0^2)/(\hat{\sigma}_1^2 + \hat{\tau}_1^2)]^{1/2}$, and $\tilde{\rho} = \hat{\rho}\hat{\sigma}_0\hat{\sigma}_1/[(\hat{\sigma}_0^2 + \hat{\tau}_0^2)(\hat{\sigma}_1^2 + \hat{\tau}_1^2)]^{1/2}$.

The calibration model can also be applied to the results of the MCS to compare the predicted measurements for the new instrument with the observed measurements from the old instrument by calculating an R^2 value and by checking the correctness of a straight regression line $y_{i0k} = \hat{y}_{i0k} + \delta_{ik}$.

Appendix S5: Standardized mean difference between instruments

Standardized mean difference between instruments were quantified using Cohen's *d* derived from the mixed-effects calibration model. Parameter estimates from the fitted model (2) were substituted directly into the effect size formula:

(3) $$d = \beta_M/\sigma_0$$

where β_M instrument represents the fixed-effect estimate for the difference between instruments, and σ_0 is the square root of the between-subject variance for the reference (current) instrument. The 95% CI for Cohen's *d* was calculated as:

(4) $$95\% \text{ CI} = d \pm 2\sqrt{2(1 + d^2/8)/(n(m-1))}$$

with n the number of participants and m the number of repeats for one instrument. Note that an absolute value for Cohen's d smaller than 0.2 is considered small, while absolute values larger than 0.8 are considered large differences.[1]

Appendix S6: Comparison of MCS and CLSA participants

To investigate the representativeness of the volunteers in the MCS with CLSA participants, Cohen's d was used to compare the effect size for all measures obtained from the old instrument between the MCS and CLSA by age groups, i.e.,

(5) $$d = \frac{|\bar{x}_1 - \bar{x}_2|}{\sqrt{\frac{(n_1-1)s_1^2 + (n_2-1)s_2^2}{n_1+n_2-2}}}$$

with $\bar{x}_i$ the average for study i (with $i = 1$ the MCS and $i = 2$ the CLSA), s_i^2 the variance of the results in study i, and n_i the number of participants in study i. Note that the repeated results for participants were first averaged out before the elements of Cohen's d were estimated.

Appendix S7: Repeatability for a single observation of the instrument

(6) $$r_j = 100\% \times \tau_j^2/[\tau_j^2 + \sigma_j^2]$$

An asymptotic confidence interval for repeatability was calculated (6), with F_L and F_U as the lower and upper 2.5% quantile of the F-distribution with 96 and 47 degrees of freedom.

(7) $$LCL_j = \hat{\tau}_j^2 / [\hat{\tau}_j^2 + F_U \hat{\sigma}_j^2],$$

$$UCL_j = \hat{\tau}_j^2 / [\hat{\tau}_j^2 + F_L \hat{\sigma}_j^2]$$

This measure of repeatability is related to an intraclass correlation coefficient of the statistical model in (1) that is familiar in epidemiology, since $ICC_j(\%) = 100\% \times (1 - r_j/100) = 100\% \times \sigma_j^2/\left[\tau_j^2 + \sigma_j^2\right]$. However, this intraclass correlation is specific to the instrument j due to the measurand it is trying to quantify and its variation in repeated observations. The smaller the repeatability the better the instrument is capable of detecting differences between individuals with a single observation. Note that the repeatability of an instrument can always be reduced by increasing the number of repeats and report the average of these repeated observations. The repeatability would then become $r_j = 100\% \times \tau_j^2/\left[\tau_j^2 + K\sigma_j^2\right]$ with K the number of repeats on which the average is calculated. A large number of repeats would drive the repeatability close to zero.

Appendix S8: STROBE Statement—Checklist of items that should be included in reports of *cross-sectional studies*

	Item No	Recommendation	Page No.
**Title and abstract**	1	(*a*) Indicate the study's design with a commonly used term in the title or the abstract	Abstract: Methods [pg.3]
		(*b*) Provide in the abstract an informative and balanced summary of what was done and what was found	Abstract: Conclusion [pg.3]
**Introduction**			
Background/ rationale	2	Explain the scientific background and rationale for the investigation being reported	Introduction: Paragraph 2 [pg.4]
Objectives	3	State specific objectives, including any prespecified hypotheses	Introduction: Paragraph 3 [pg.5]
**Methods**			
Study design	4	Present key elements of study design early in the paper	Methods: Paragraphs 1-2 [pg.5]
Setting	5	Describe the setting, locations, and relevant dates, including periods of recruitment, exposure, follow-up, and data collection	Methods: Paragraphs 2 [pg.5]
Participants	6	(*a*) Give the eligibility criteria, and the sources and methods of selection of participants	Methods: Paragraphs 1 [pg.5]
Variables	7	Clearly define all outcomes, exposures, predictors, potential confounders, and effect modifiers. Give diagnostic criteria, if applicable	Methods: Instrument Measures [pg.6]
Data sources/ measurement	8*	For each variable of interest, give sources of data and details of methods of assessment (measurement). Describe comparability of assessment methods if there is more than one group	
Bias	9	Describe any efforts to address potential sources of bias	Methods: Paragraphs 2 [pg.3]
Study size	10	Explain how the study size was arrived at	Methods: Paragraphs 1 [pg.5] (details in Supplementary Materials)
Quantitative variables	11	Explain how quantitative variables were handled in the analyses. If applicable, describe which groupings were chosen and why	Methods: Statistical Analyses [pg.6]

Statistical methods	12	(*a*) Describe all statistical methods, including those used to control for confounding	Methods: Statistical Analyses [pg.6]
		(*b*) Describe any methods used to examine subgroups and interactions	NA
		(*c*) Explain how missing data were addressed	Linear mixed models were used for analyses under the assumption of missing at random.
		(*d*) If applicable, describe analytical methods taking account of sampling strategy	
		(*e*) Describe any sensitivity analyses	Methods: Sensitivity analysis [pg.7]
**Results**			
Participants	13*	(a) Report numbers of individuals at each stage of study—eg numbers potentially eligible, examined for eligibility, confirmed eligible, included in the study, completing follow-up, and analysed	Results: Paragraph 1 [pg.8]
		(b) Give reasons for non-participation at each stage	NA
		(c) Consider use of a flow diagram	NA
Descriptive data	14*	(a) Give characteristics of study participants (eg demographic, clinical, social) and information on exposures and potential confounders	Results: Paragraph 1 [pg.8]
		(b) Indicate number of participants with missing data for each variable of interest	Left cIMT (n=49); All other variable had at least one data point for each variable.
Outcome data	15*	Report numbers of outcome events or summary measures	[pg.15] Table 2: Descriptive statistics for key variables obtained from the old and new instruments
Main results	16	(*a*) Give unadjusted estimates and, if applicable, confounder-adjusted estimates and their precision (eg, 95% confidence	Results [pg.8]; Table 2 [pg.15];

		interval). Make clear which confounders were adjusted for and why they were included	Supplementary Tables 1-7
		(*b*) Report category boundaries when continuous variables were categorized	NA
		(*c*) If relevant, consider translating estimates of relative risk into absolute risk for a meaningful time period	NA
Other analyses	17	Report other analyses done—eg analyses of subgroups and interactions, and sensitivity analyses	Supplementary Tables 1C-7C
**Discussion**			
Key results	18	Summarise key results with reference to study objectives	Discussion: Paragraph 1 [pg.10]
Limitations	19	Discuss limitations of the study, taking into account sources of potential bias or imprecision. Discuss both direction and magnitude of any potential bias	Discussion: Strengths and Limitations [pg.11]
Interpretation	20	Give a cautious overall interpretation of results considering objectives, limitations, multiplicity of analyses, results from similar studies, and other relevant evidence	Discussion: Conclusion [pg.12]
Generalisabilit y	21	Discuss the generalisability (external validity) of the study results	Discussion: Strengths [pg.11]
**Other information**			
Funding	22	Give the source of funding and the role of the funders for the present study and, if applicable, for the original study on which the present article is based	

*Give information separately for exposed and unexposed groups.

Note: An Explanation and Elaboration article discusses each checklist item and gives methodological background and published examples of transparent reporting. The STROBE checklist is best used in conjunction with this article (freely available on the Web sites of PLoS Medicine at http://www.plosmedicine.org/, Annals of Internal Medicine at http://www.annals.org/, and Epidemiology at http://www.epidem.com/). Information on the STROBE Initiative is available at www.strobe-statement.org.

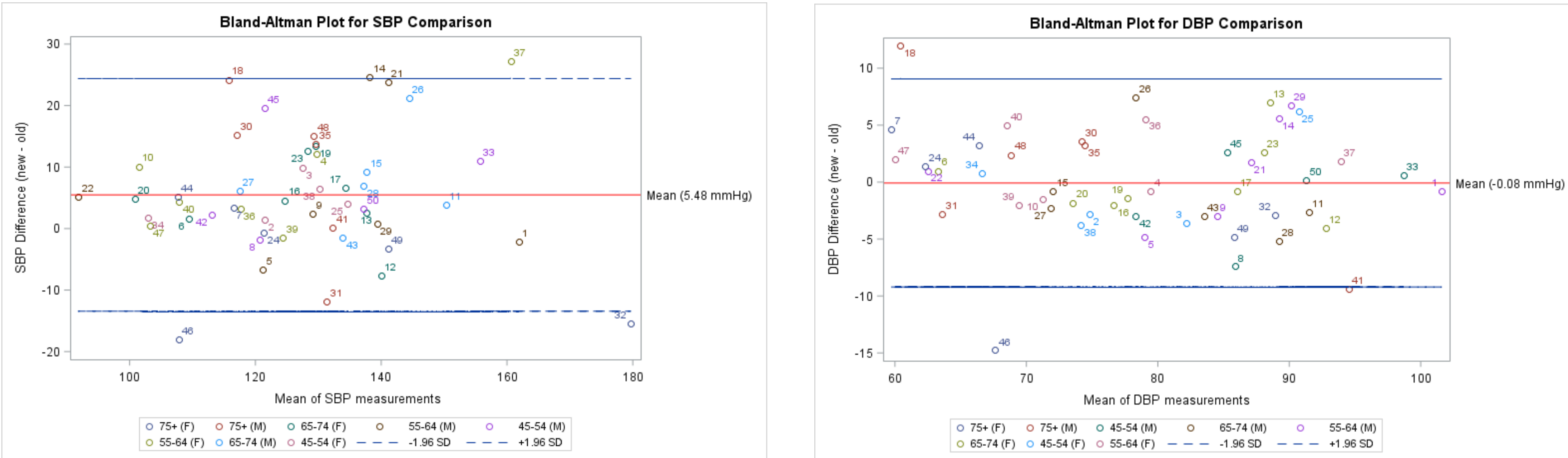

Figure S1A-B: Bland-Altman plot comparing systolic and diastolic blood pressure

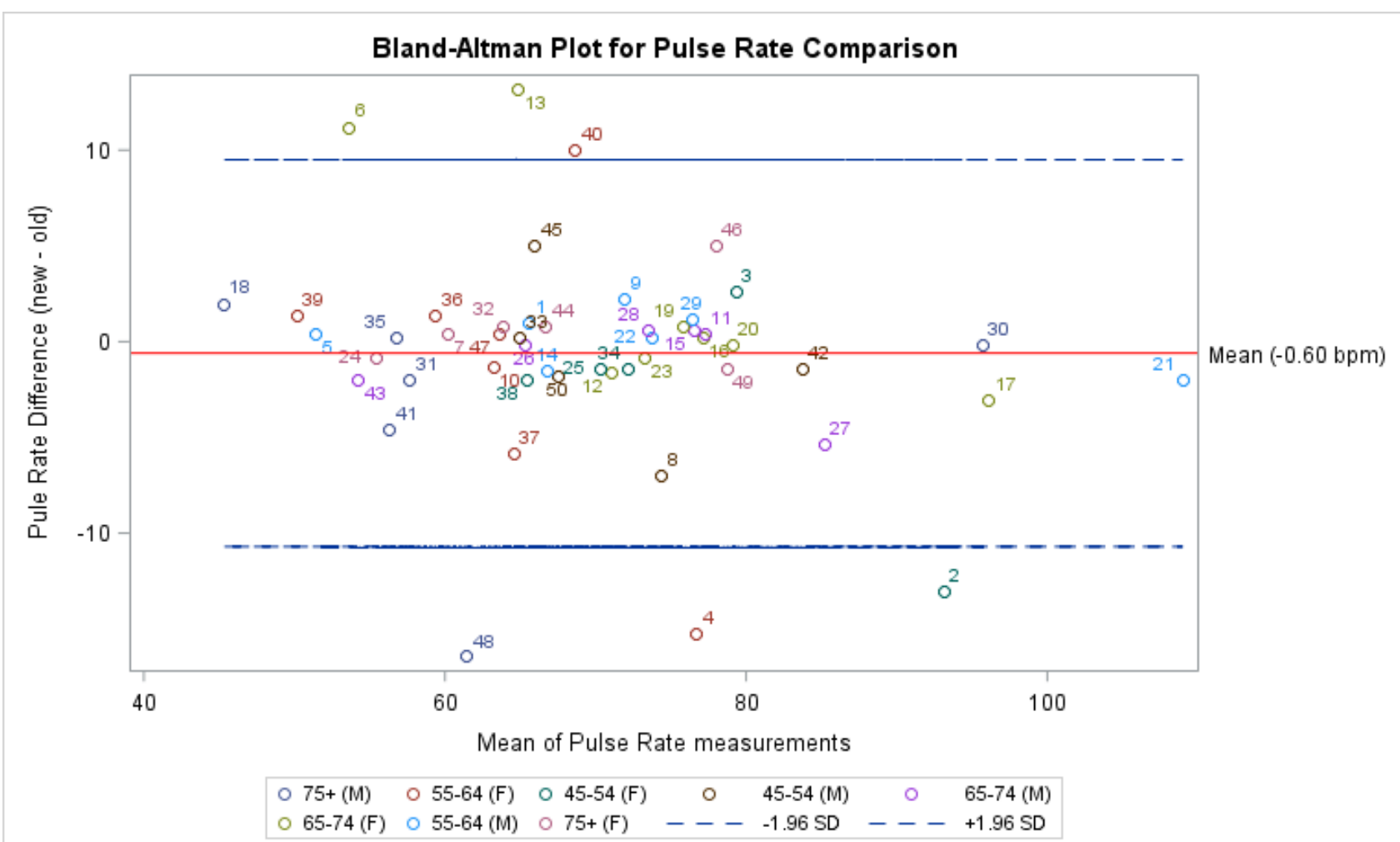

Figure S1C: Bland-Altman plot comparing pulse rate

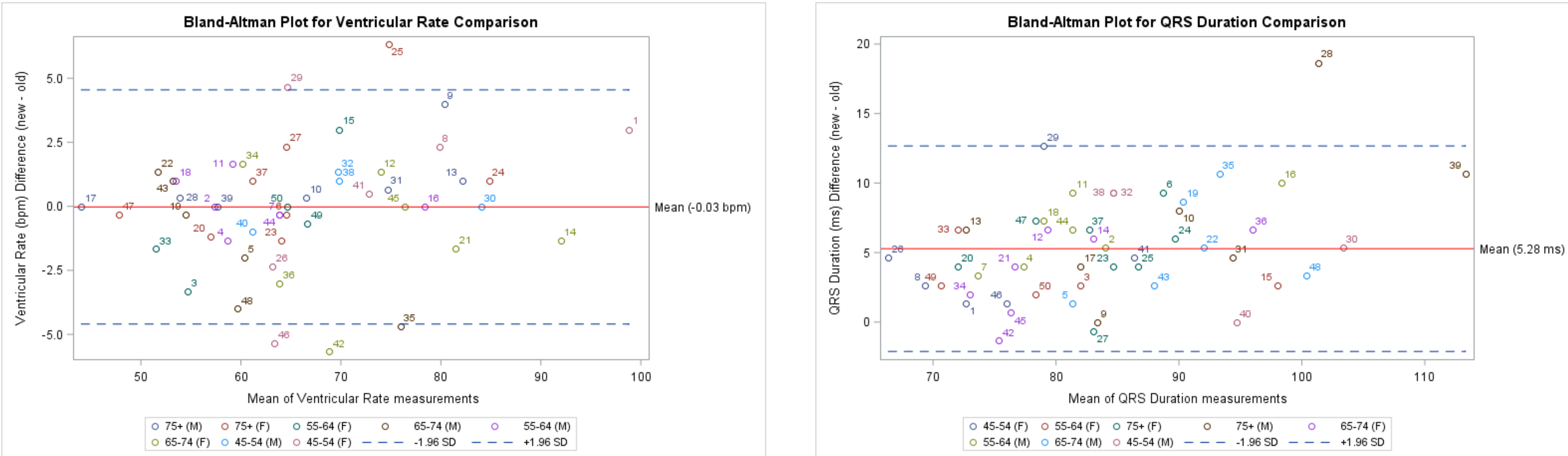

Figure S2A-B: Bland-Altman plot comparing ventricular rate and QRS duration

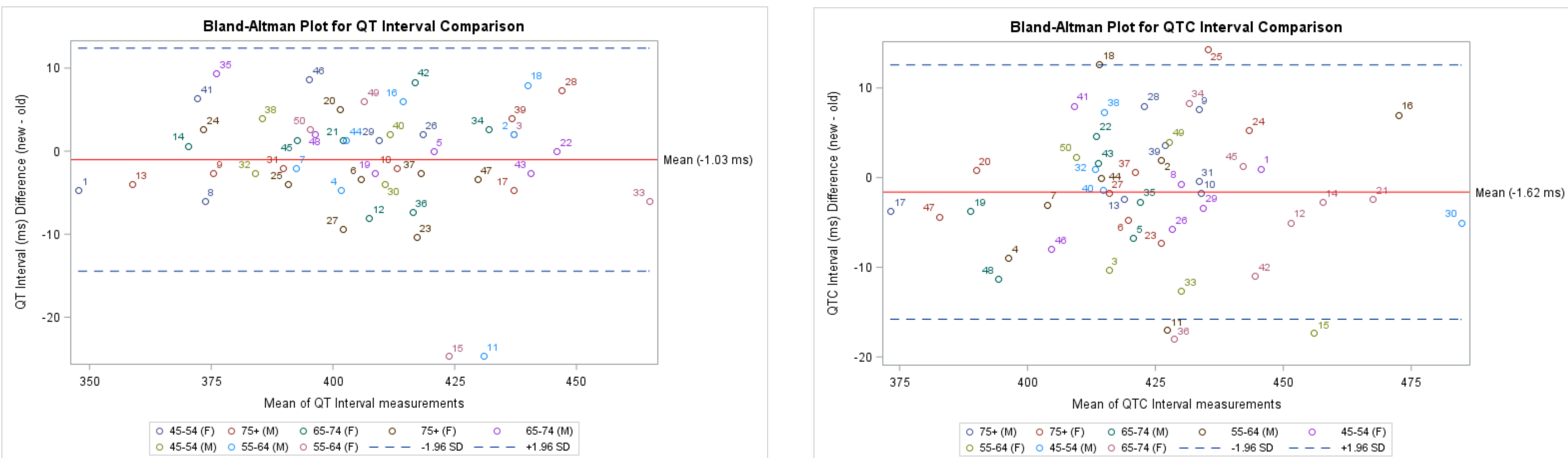

Figure S2C-D: Bland-Altman plot comparing QT and QTC interval

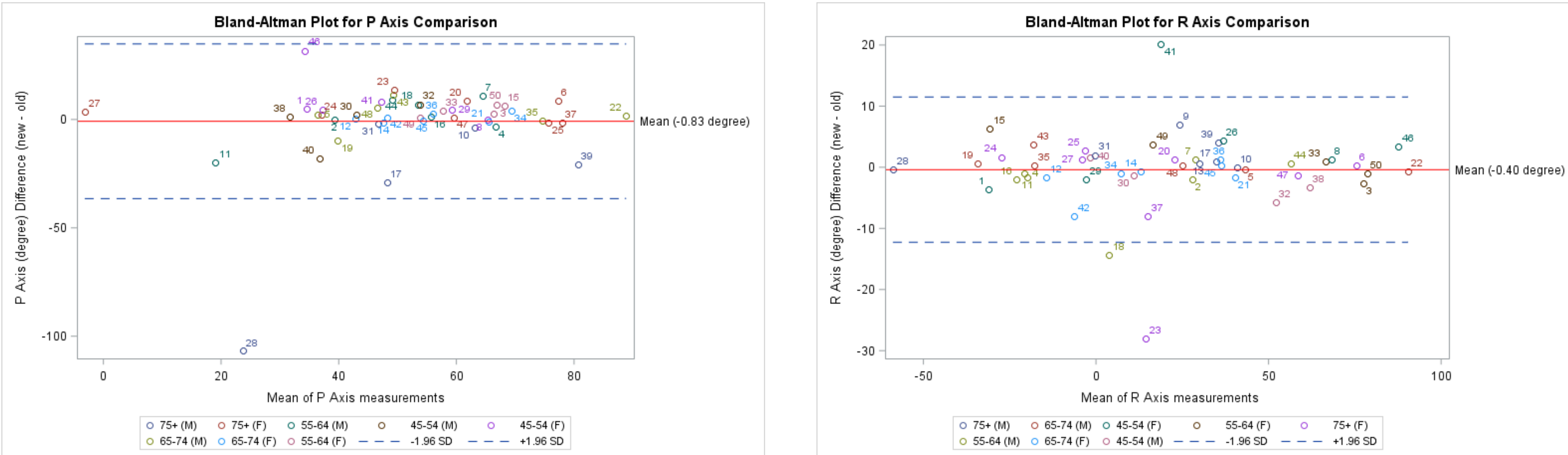

Figure S2E-F: Bland-Altman plot comparing P and R axis

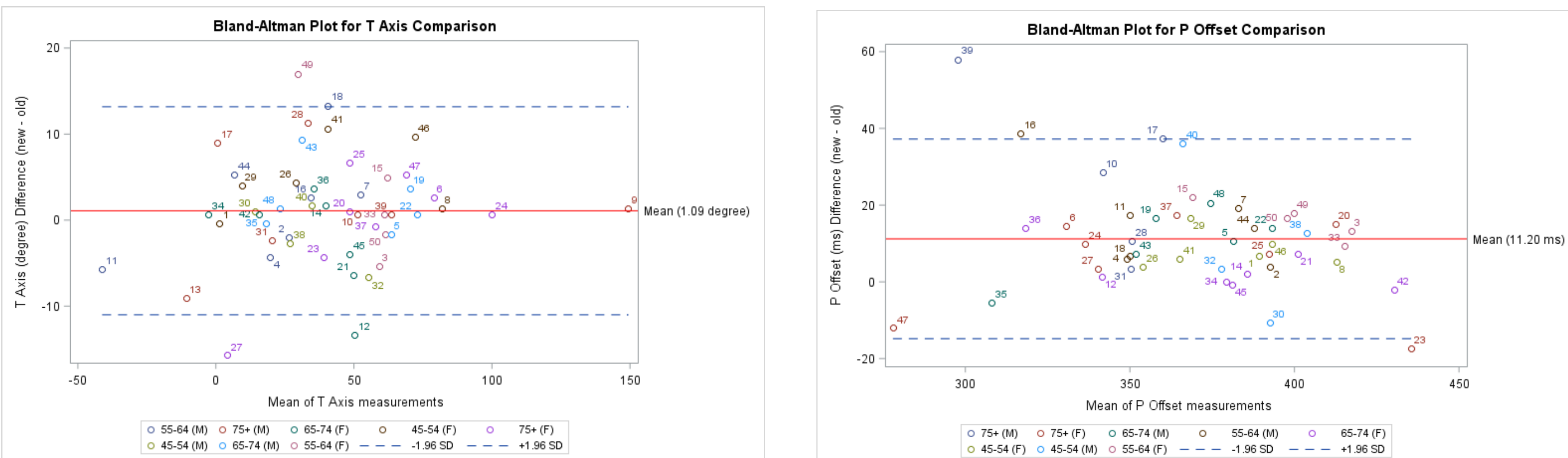

Figure S2G-H: Bland-Altman plot comparing T axis and P offset

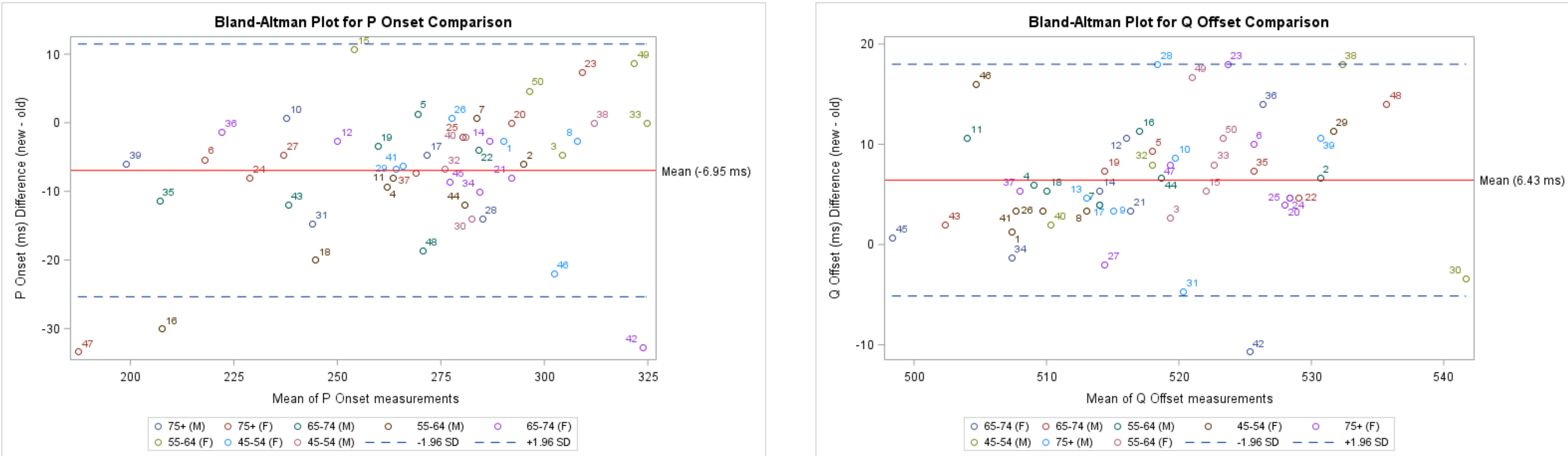

Figure S2I-J: Bland-Altman plot comparing P onset and Q offset

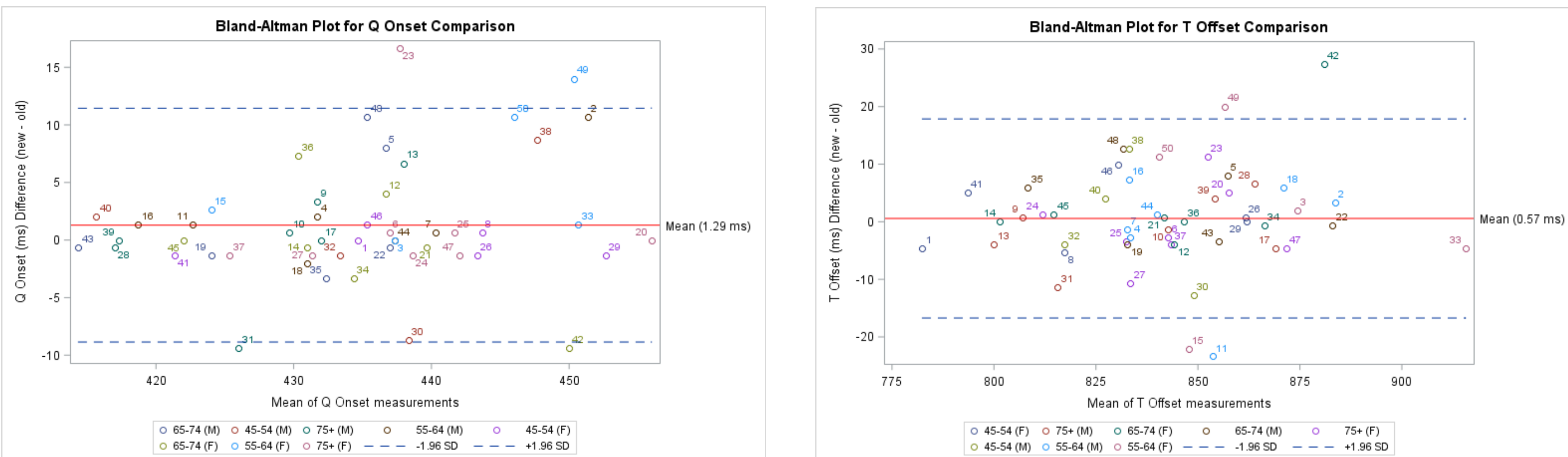

Figure S2K-L: Bland-Altman plot comparing Q onset and T offset

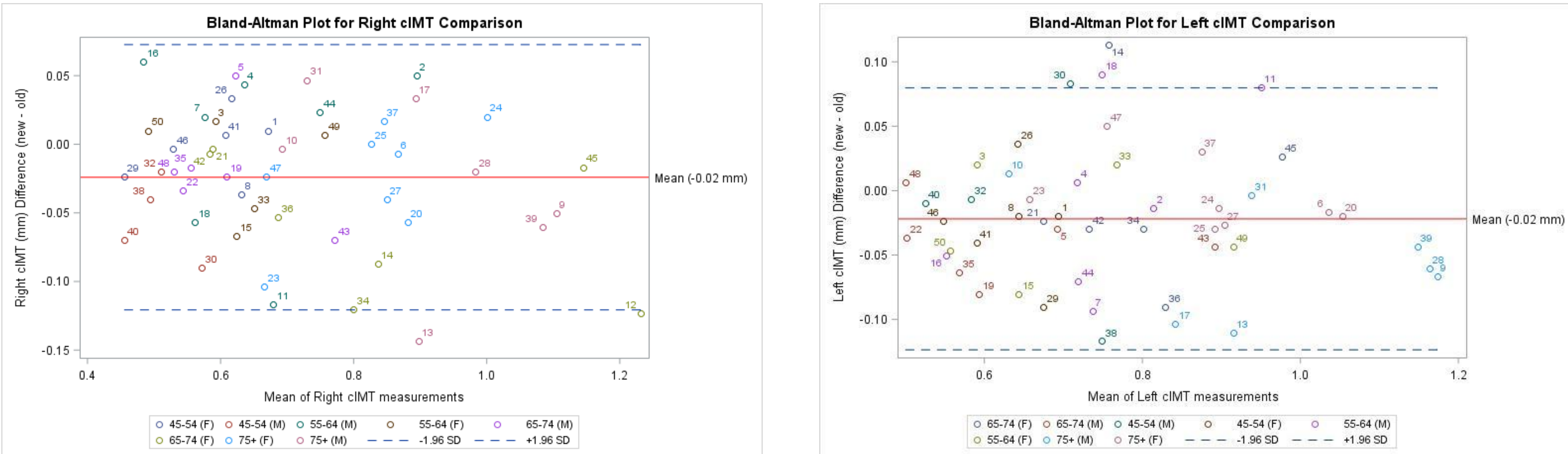

Figure S3A-B: Bland-Altman plot comparing cIMT (right and left)

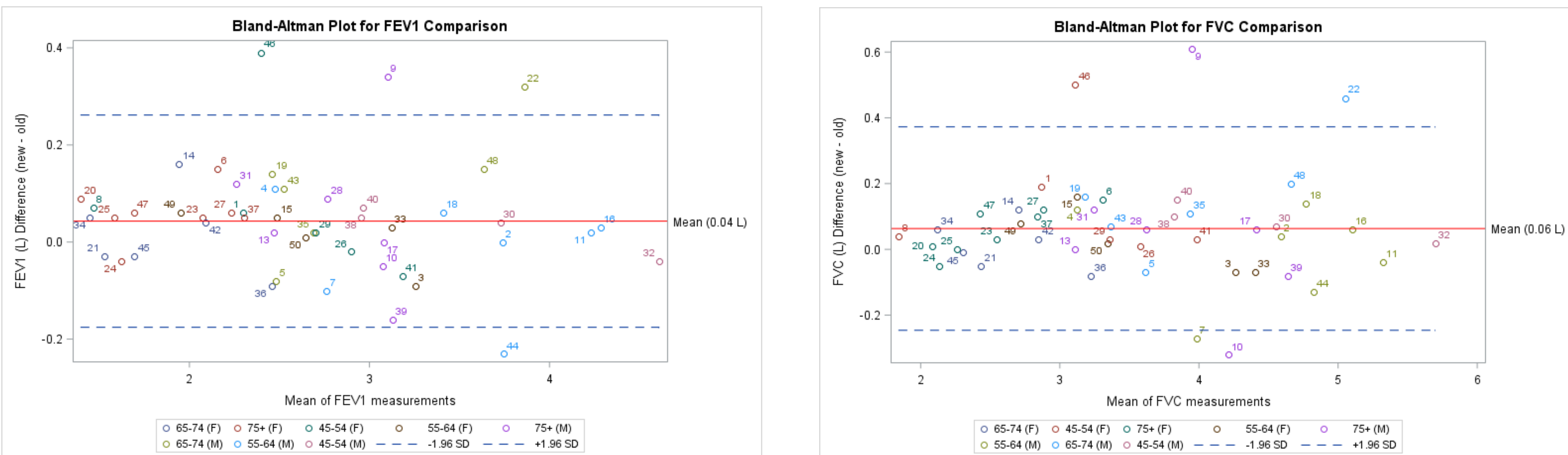

Figure S4A-B: Bland-Altman plot comparing FEV1 and FVC (using highest value)

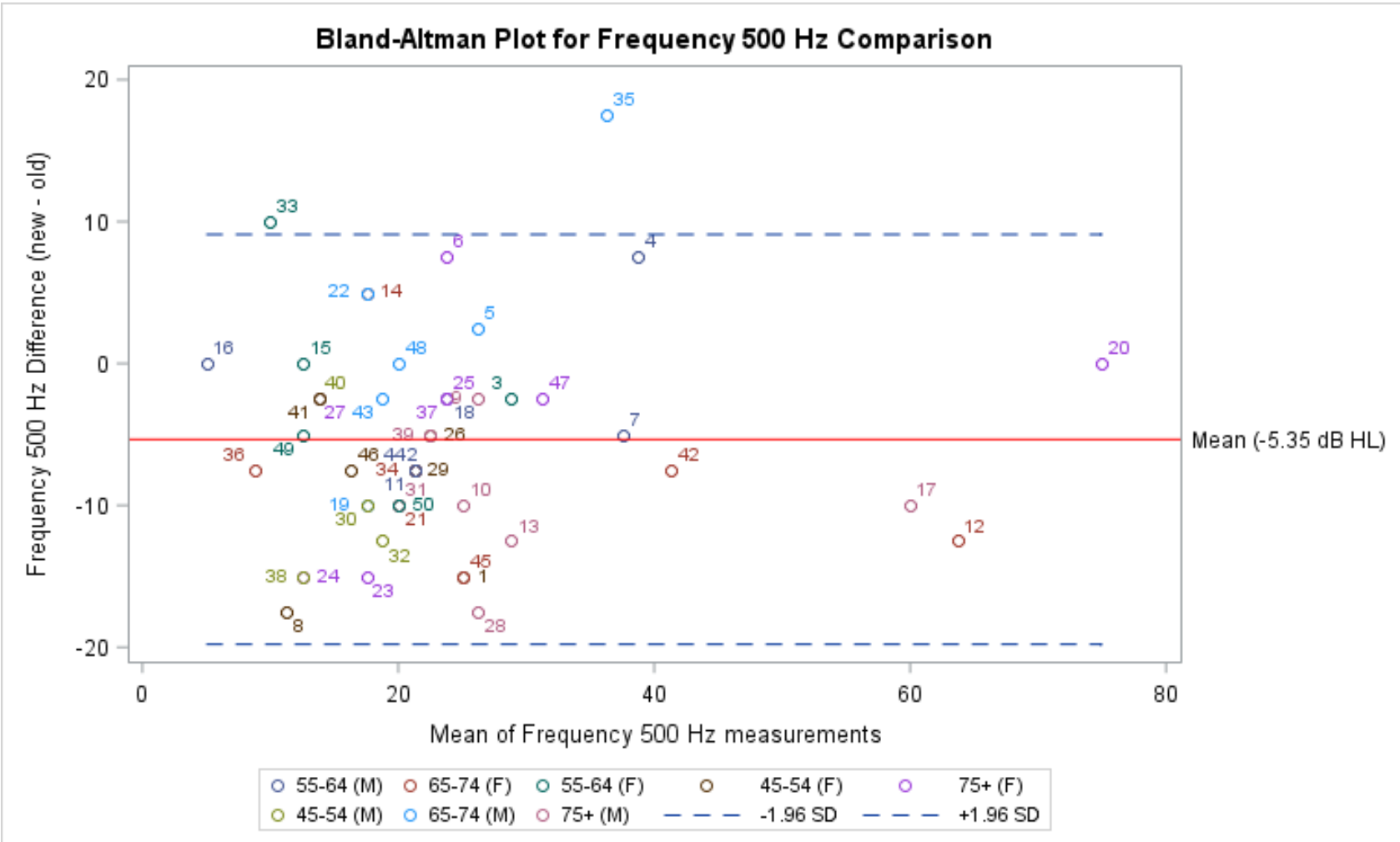

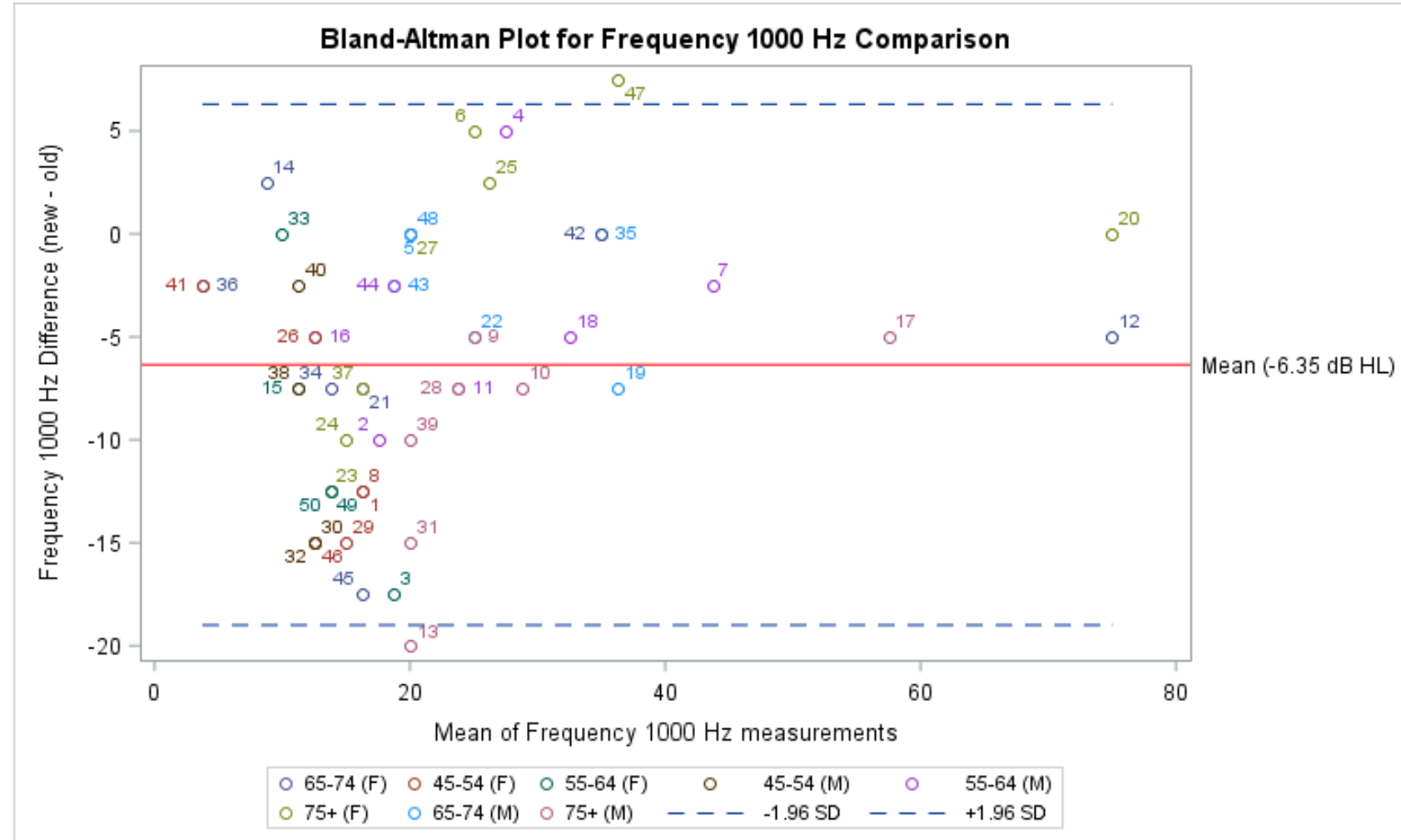

Figure S5A-B: Bland-Altman plot comparing at decibels hearing level (dB HL) at Frequency 500 and 1000 Hz

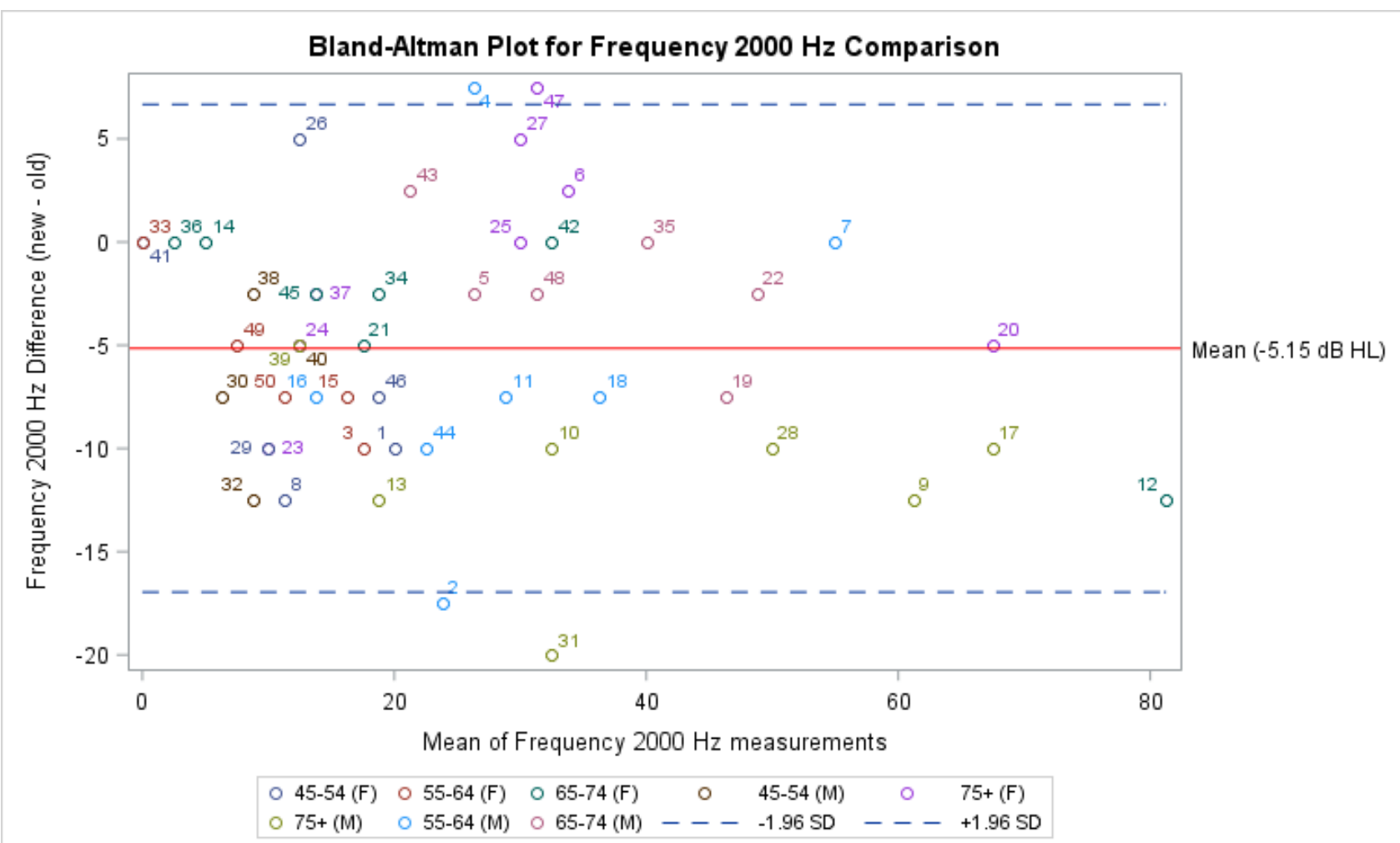

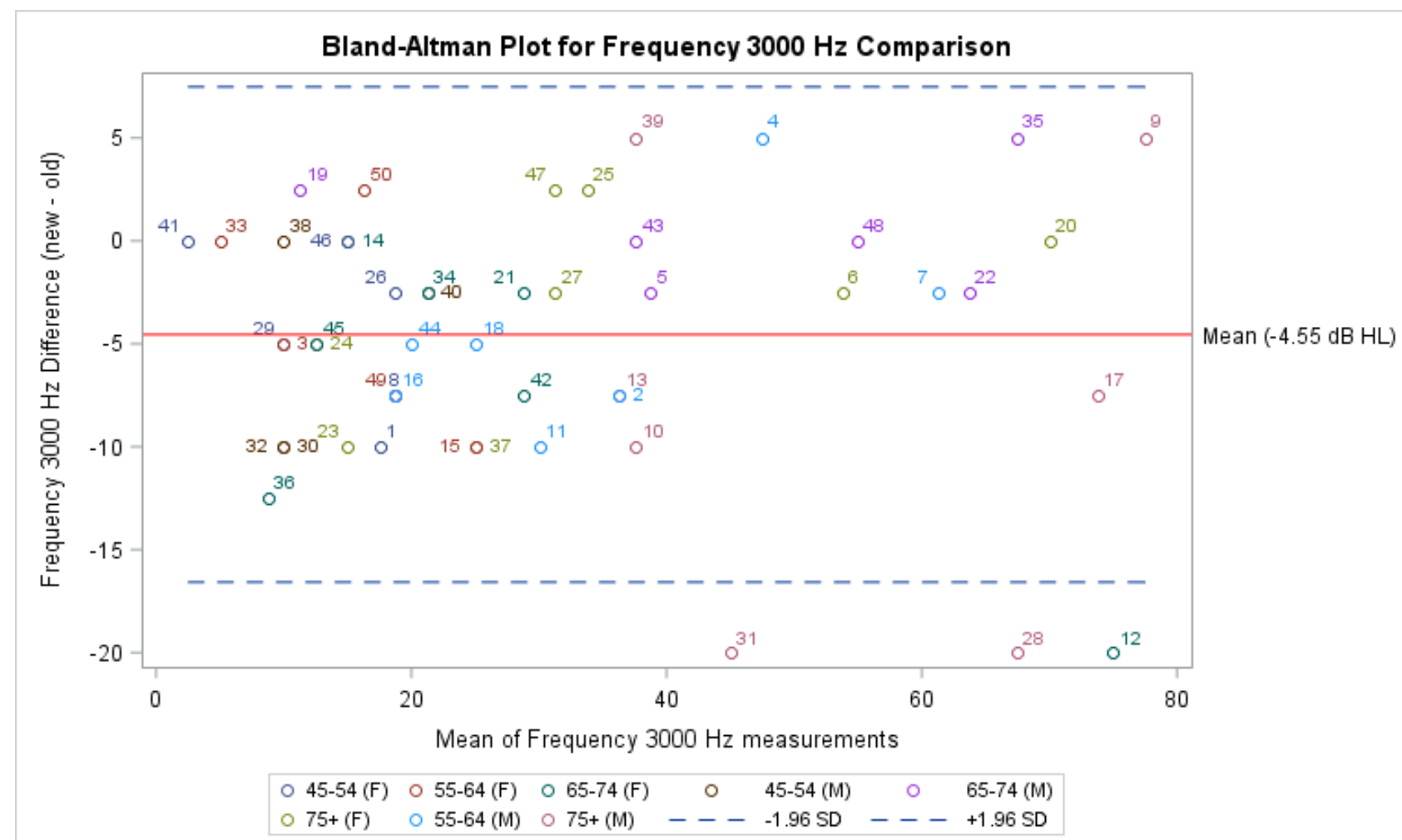

Figure S5C-D: Bland-Altman plot comparing at decibels hearing level (dB HL) at Frequency 2000 and 3000 Hz

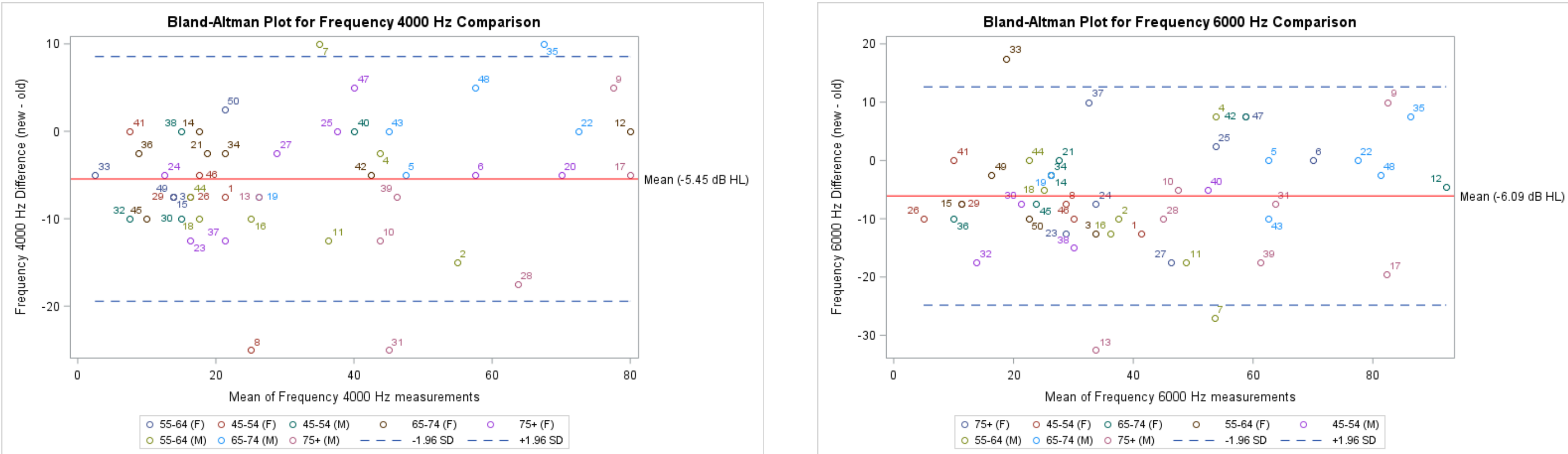

Figure S5E-F: Bland-Altman plot comparing at decibels hearing level (dB HL) at Frequency 4000 and 6000 Hz

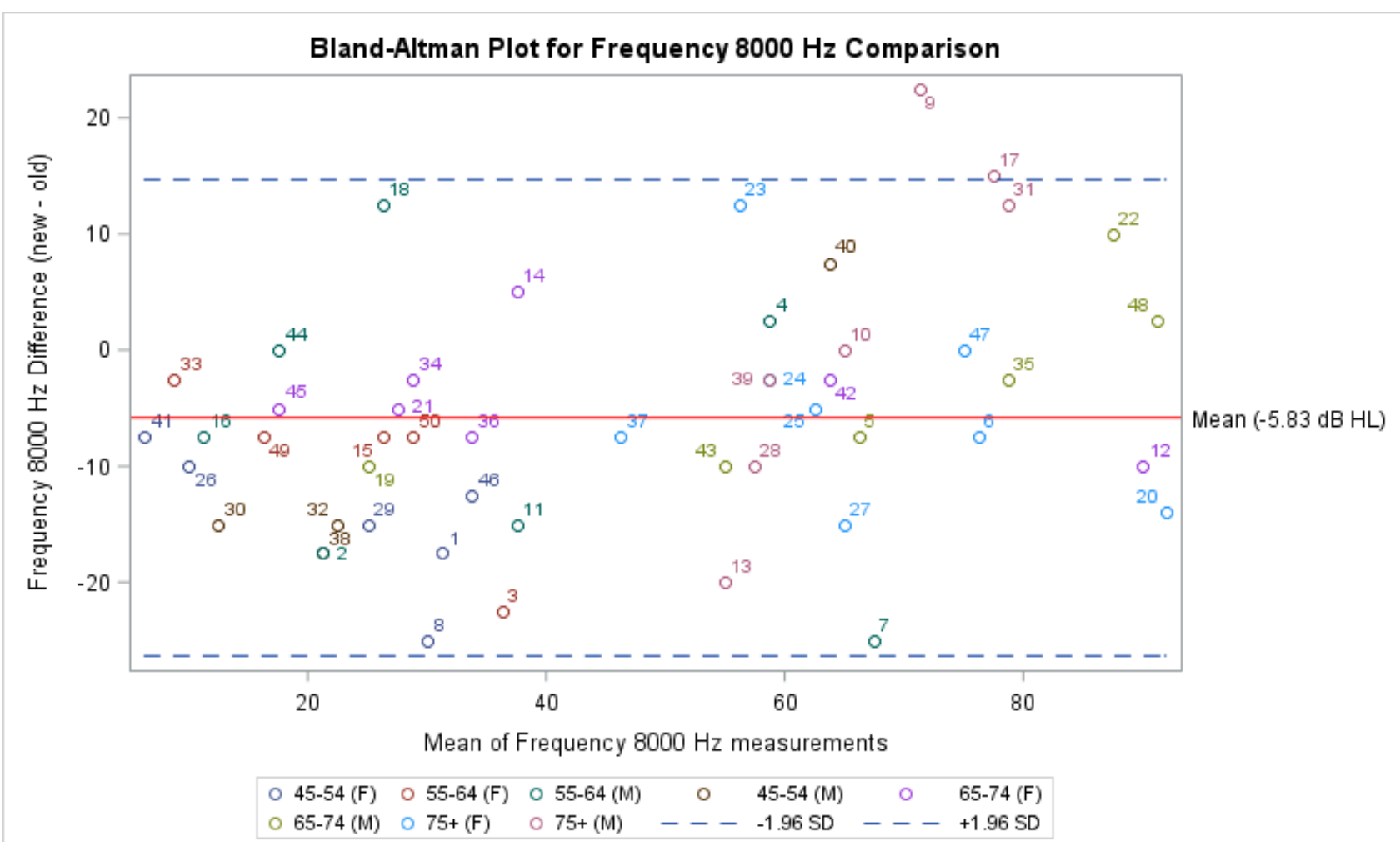

Figure S5G: Bland-Altman plot comparing at decibels hearing level (dB HL) at Frequency 8000 Hz

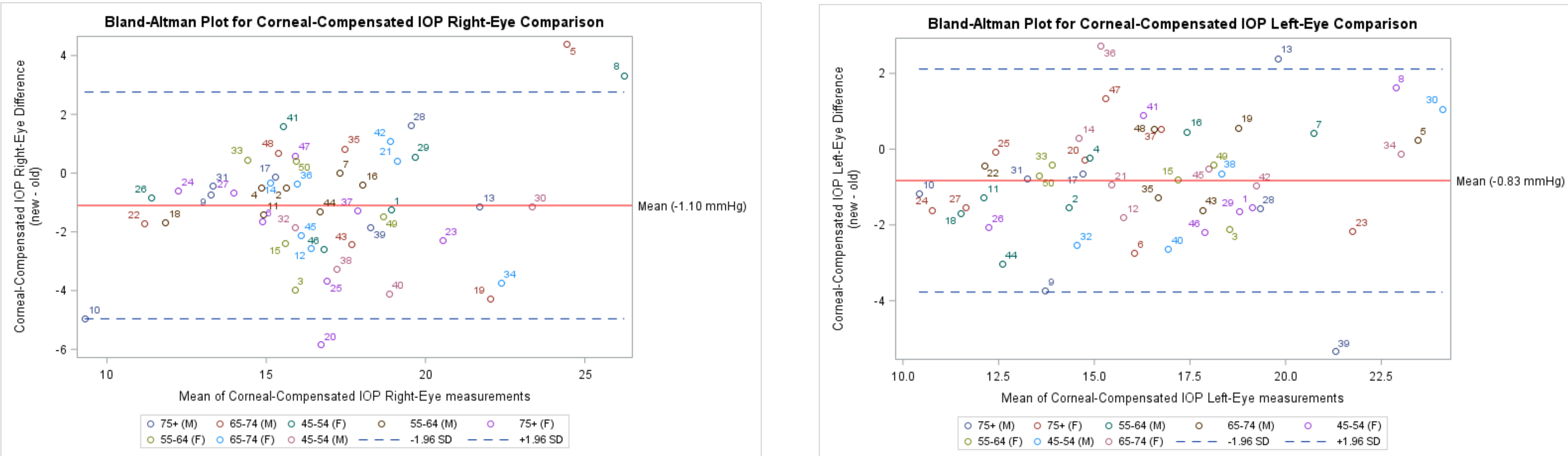

Figure S6A-B: Bland-Altman plot comparing corneal compensated IOP (right and left eye)

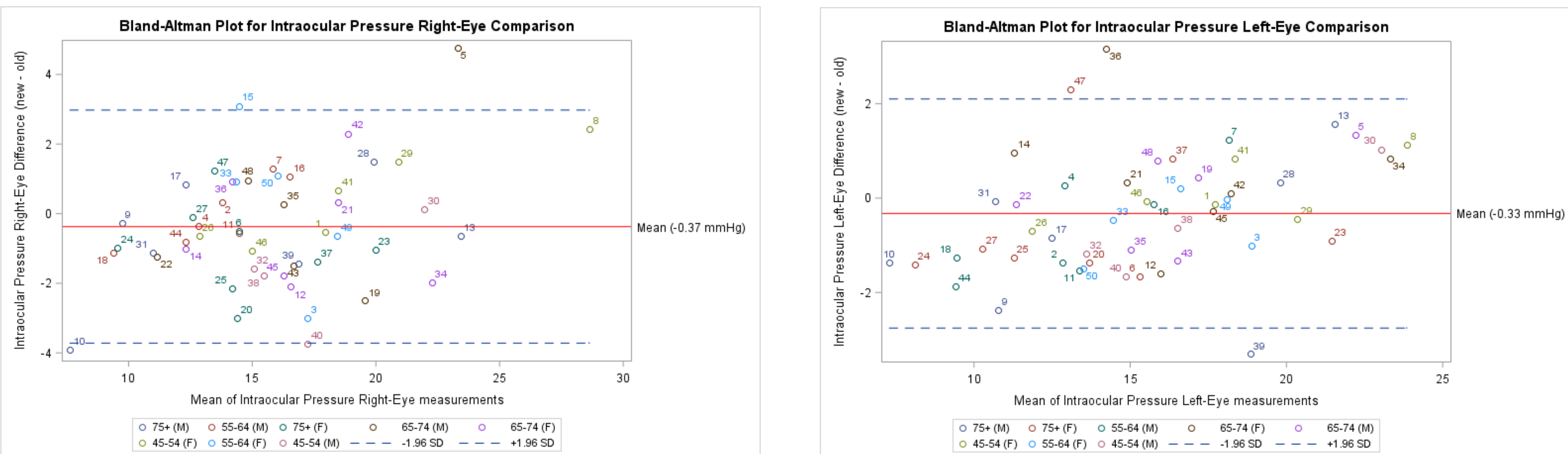

Figure S6C-D: Bland-Altman plot comparing intraocular pressure (right and left eye)

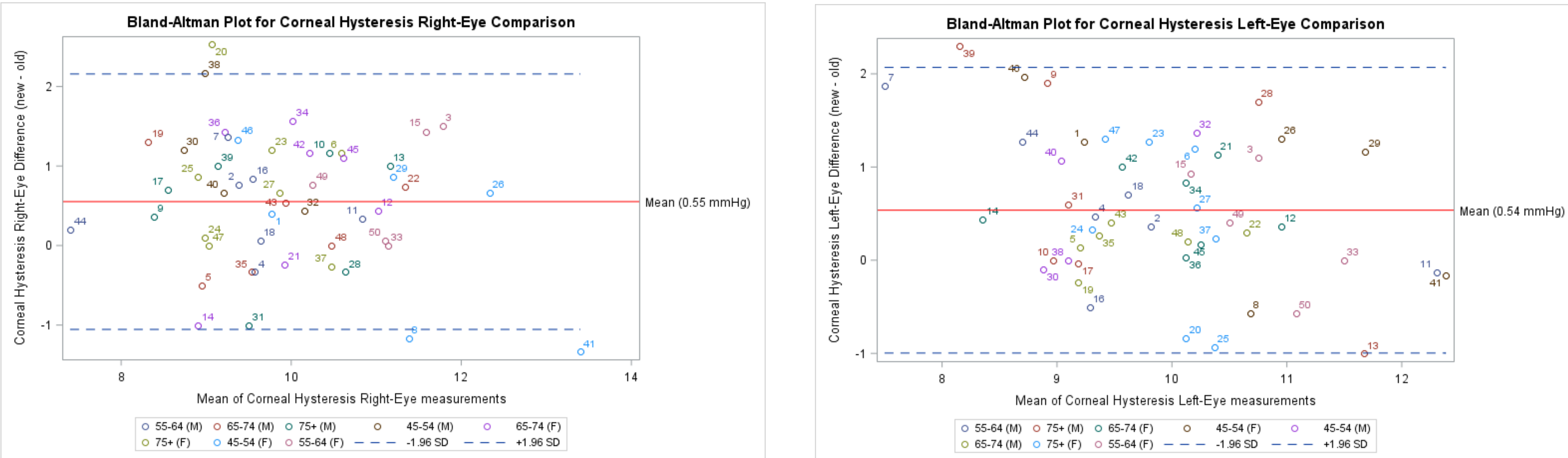

Figure S6E-F: Bland-Altman plot comparing corneal hysteresis (right and left eye)

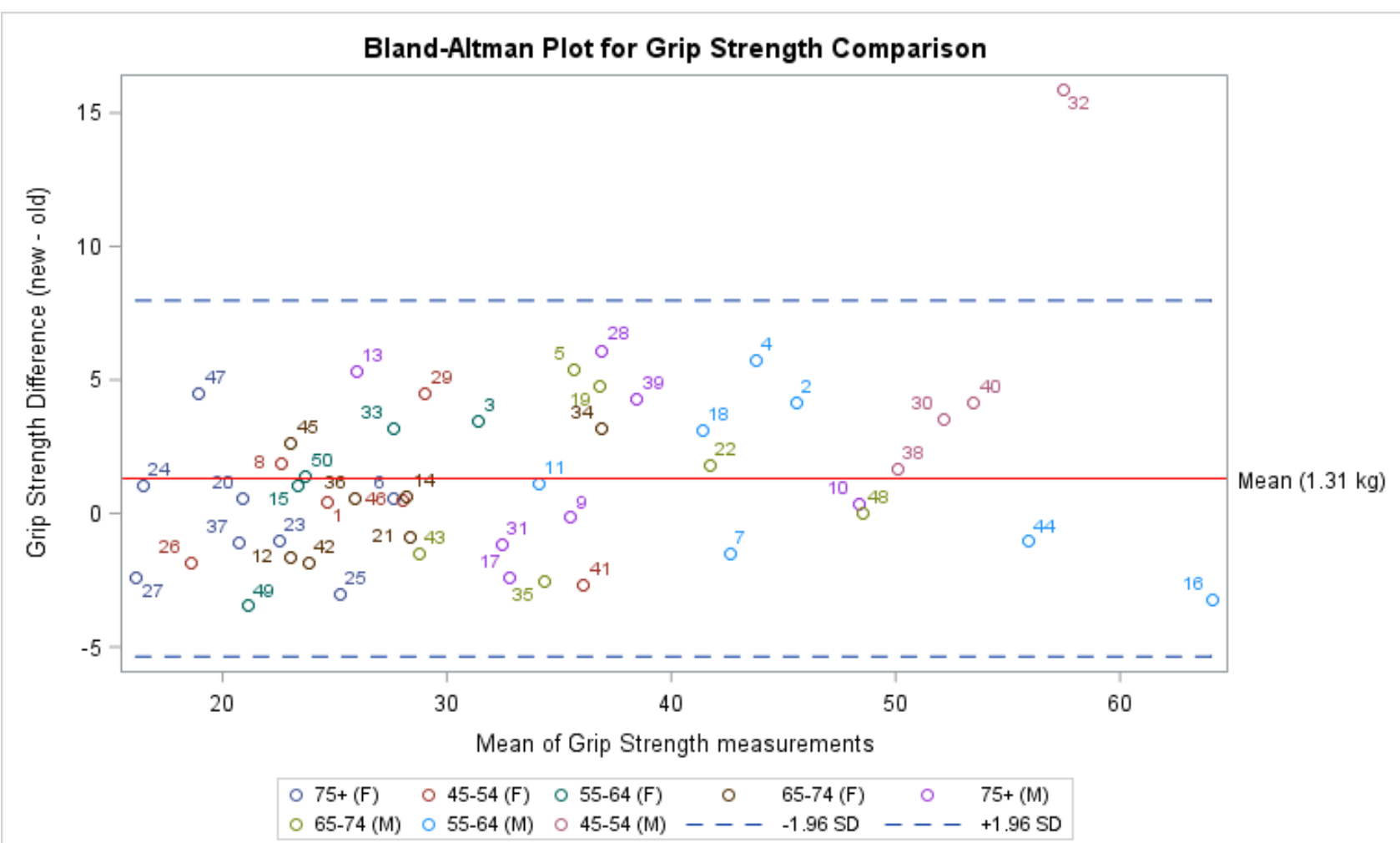

Figure S7: Bland-Altman plot comparing grip strength

Table S1: Bland-Altman agreement for key variables between old and new instruments

	Agreement
**Blood pressure monitor**	
Systolic blood pressure (mmHg)	5.48 (-13.43, 24.40)
Diastolic blood pressure (mmHg)	-0.08 (-9.21, 9.06)
Pulse rate (bpm)	-0.60 (-10.70, 9.50)
**Spirometer (L)**	
FEV1	0.04 (-0.18, 0.26)
FVC	0.06 (-0.25, 0.37)
**Carotid ultrasound (mm)**	
cIMT right	-0.02 (-0.12, 0.07)
cIMT left	-0.02 (-0.12, 0.08)
**Grip strength (kg)**	1.31 (-5.36, 7.98)
**Audiometer (dB HL)**	
Frequency 500 Hz	-5.35 (-19.80, 9.10)
Frequency 1000 Hz	-6.35 (-19.00, 6.30)
Frequency 2000 Hz	-5.15 (-16.96, 6.66)
Frequency 3000 Hz	-4.55 (-16.58, 7.48)
Frequency 4000 Hz	-5.45 (-19.44, 8.54)
Frequency 6000 Hz	-6.09 (-24.81, 12.63)
Frequency 8000 Hz	-5.83 (-26.34, 14.68)
**Tonometer (mmHg)**	
Corneal-compensated IOP - right-eye	-1.10 (-4.96, 2.76)
Corneal-compensated IOP - left-eye	-0.83 (-3.77, 2.11)
Intraocular pressure - right-eye	-0.37 (-3.72, 2.98)
Intraocular pressure - left-eye	-0.33 (-2.75, 2.10)
Corneal hysteresis - right-eye	0.55 (-1.05, 2.16)
Corneal hysteresis - left-eye	0.54 (-1.00, 2.07)
**Electrocardiogram (ECG)**	
Ventricular rate (bpm)	-0.03 (-4.60, 4.55)
QRS duration (ms)	5.28 (-2.11, 12.67)
QT interval (ms)	-1.03 (-14.45, 12.40)
QTC interval (ms)	-1.62 (-15.80, 12.57)
P axis (degree)	-0.83 (-36.51, 34.85)
R axis (degree)	-0.40 (-12.27, 11.48)
T axis (degree)	1.09 (-11.00, 13.18)
P offset (ms)	11.20 (-14.82, 37.22)
P onset (ms)	-6.95 (-25.37, 11.47)
Q offset (ms)	6.65 (-4.01, 17.30)
Q onset (ms)	1.29 (-8.86, 11.44)
T offset (ms)	0.57 (-16.72, 17.87)

Table S2A: Covariance Parameter Estimates for Variability Analysis in SBP, DBP, and Pulse Rate

**Covariance Parameter Estimates**					
			**Estimate**		
**Cov Parm**	Subject	Group	**Systolic Blood Pressure (SBP)**	**Diastolic Blood Pressure (DBP)**	**Pulse Rate**
**-Var(1)**	ID		324.42	99.982	145.92
**Var(2)**	ID		327.57	122.31	184.45
**Corr(2,1)**	ID		0.9027	0.9581	0.9541
**Residual**	ID	GROUP NEW	44.3190	17.6981	10.0419
**Residual**	ID	GROUP OLD	39.4739	14.2639	5.8224

Table S2B: Mixed effects models comparing SBP, DBP, and Pulse Rate between the old and new instruments

	**SBP**		**DBP**		**Pulse Rate**	
	**Estimate (95% CI)**	**p-value**	**Estimate (95% CI)**	**p-value**	**Estimate (95% CI)**	**p-value**
**Intercept**	120.94 (108.50, 133.38)	<0.0001	67.89 (60.68, 75.10)	<0.0001	63.15 (54.58, 71.72)	<0.0001
**45-54**	-0.56 (-15.86, 14.74)	0.9413	10.08 (1.31, 18.85)	0.0276	9.22 (-1.15, 19.59)	0.0800
**55-64**	-0.26 (-14.32, 13.80)	0.9701	8.23 (0.17, 16.29)	0.0463	4.75 (-4.78, 14.29)	0.3206
**65-74**	4.34 (-9.86, 18.33)	0.5480	8.59 (0.51, 16.67)	0.0405	10.45 (0.90, 20.01)	0.0328
**Sex (Male)**	7.46 (-2.96, 17.61)	0.1458	5.87 (0.05, 11.69)	0.0481	0.42 (-6.46, 7.30)	0.9030
**Test order (new first)**	-0.53 (-10.87, 9.82)	0.9188	3.36 (-2.57, 9.29)	0.2464	2.15 (-4.87, 9.16)	0.5408
**Instrument (New)**	5.19 (2.64, 7.74)	0.0002	-0.10 (-1.28, 1.07)	0.8609	-1.16 (-2.45, 0.13)	0.0766

Reference: Age = 75+, Sex = Female; Test order = Old instrument; Instrument = Old.

Table S2C: Mixed effects models comparing log transformed SBP, DBP, and Pulse Rate between old and new instruments

	SBP		DBP		Pulse Rate	
	**Estimate (95% CI)**	**p-value**	**Estimate (95% CI)**	**p-value**	**Estimate (95% CI)**	**p-value**
**Intercept**	4.78<br>4.68, 4.88	<0.0001	4.21<br>4.11, 4.30	<0.0001	4.13<br>4.01, 4.25	<0.0001
**45-54**	0.00<br>-0.12, 0.12	0.9730	0.13<br>0.02, 0.25	0.0205	0.15<br>0.01, 0.30	0.0411
**55-64**	-0.00<br>-0.11, 0.11	0.9451	0.11<br>0.00, 0.21	0.0412	0.07<br>-0.06, 0.21	0.2666
**65-74**	0.04<br>-0.07, 0.15	0.4627	0.12<br>0.02, 0.22	0.0252	0.16<br>0.02, 0.29	0.0220
**Sex (Male)**	0.06<br>-0.02, 0.14	0.1107	0.08<br>0.00, 0.15	0.0430	-0.00<br>-0.10, 0.09	0.9263
**Test order (new first)**	-0.00<br>-0.08, 0.08	0.9274	0.05<br>-0.03, 0.12	0.2245	0.03<br>-0.06, 0.13	0.4835
**Instrument (New)**	0.04<br>0.02, 0.06	<0.0001	0.00<br>-0.02, 0.02	0.9613	-0.01<br>-0.03, 0.00	0.1273

Reference: Age = 75+, Sex = Female; Test order = Old instrument; Instrument = Old.

Percentage change: SBP = 4.18%; DBP= 0.04%; Pulse Rate = 1.44%

Table S3A: Covariance Parameter Estimates for Variability Analysis in ECG measures

Covariance Parameter Estimates								
			Estimate					
**Cov Parm**	Subject	Group	**Ventricular Rate**	**QRS Duration**	**QT Interval**	**QTC Interval**	**P Axis**	**R Axis**
**Var(1)**	ID		35.7174	93.7876	303.17	339.32	461.04	1371.83
**Var(2)**	ID		31.1953	71.3404	327.65	290.01	280.35	1355.57
**Corr(2,1)**	ID		1	0.9442	0.9533	1	0.6759	0.9903
**Residual**	ID	GROUP NEW	5.264	6.8267	20.7483	48.5586	26.7014	10.04
**Residual**	ID	GROUP OLD	6.4644	3.942	29.1147	102.1	121.37	17.4662
			**Estimate**					
**Cov Parm**	Subject	Group	**T Axis**	**P offset**	**P onset**	**Q offset**	**Q onset**	**T offset**
**Var(1)**	ID		1063.01	4971.32	2802.9	109.21	101.79	394.27
**Var(2)**	ID		967.82	4956.78	2397.26	71.2263	79.3525	368.44
**Corr(2,1)**	ID		0.9939	0.989	0.9924	1	1	0.9545
**Residual**	ID	GROUP NEW	14.6846	62.2133	56.3733	29.9353	40.5219	65.0396
**Residual**	ID	GROUP OLD	54.7693	144.53	72.6959	54.4834	46.0092	63.3418

Table S3B: Mixed effects models comparing ECG measures between the old and new instruments

	Ventricular Rate		QRS Duration		QT Interval		QTC Interval		P Axis		R Axis	
	**Estimate (95% CI)**	**p-value**	**Estimate (95% CI)**	**p-value**	**Estimate (95% CI)**	**p-value**	**Estimate (95% CI)**	**p-value**	**Estimate (95% CI)**	**p-value**	**Estimate (95% CI)**	**p-value**
**Intercept**	65.39<br>61.50, 69.27	<0.0001	78.02<br>72.34, 83.70	<0.0001	405.50<br>393.62, 417.39	<0.0001	419.24<br>407.18, 431.31	<0.0001	54.61<br>42.22, 67.00	<.0001	24.11<br>-0.65, 48.87	0.0560
**45-54**	-0.04<br>-5.01, 4.92	0.9857	-4.37<br>-11.63, 2.88	0.2309	-1.28<br>-16.37, 13.81	0.8646	-0.54<br>-15.98, 14.90	0.9445	-12.95<br>-28.81, 2.91	0.1067	15.51<br>-16.18, 47.19	0.3292
**55-64**	0.82<br>-3.75, 5.39	0.7189	-5.15<br>-11.82, 1.52	0.1267	8.03<br>-5.84, 21.91	0.2493	12.37<br>-1.46, 26.92	0.0774	-0.48<br>-14.72, 13.77	0.9465	4.08<br>-25.05, 33.21	0.7788
**65-74**	1.98<br>-2.45, 6.40	0.3724	-0.13<br>-6.60, 6.33	0.9672	5.96<br>-7.49, 19.41	0.3766	10.69<br>-3.06, 24.45	0.1242	-0.35<br>-14.34, 13.65	0.9605	-1.60<br>-29.84, 26.63	0.9093
**Sex (Male)**	-2.51<br>-5.86, 0.84	0.1376	7.56<br>2.68, 12.45	0.0032	1.83<br>-8.33, 12.00	0.7181	-7.37<br>-17.76, 3.03	0.1603	-1.36<br>-11.77, 9.05	0.7933	-10.31<br>-31.66, 11.03	0.3353
**Heart rate**	0.78<br>0.64, 0.93	<0.0001	0.07<br>-0.14, 0.29	0.4859	-1.36<br>-1.80, -0.92	<0.0001	1.06<br>0.61, 1.51	<0.0001	0.06<br>-0.39, 0.52	0.7788	-0.38<br>-1.31, 0.54	0.4070
**Test order (new first)**	3.01<br>-0.33, 6.35	0.0762	3.73<br>-1.15, 8.61	0.1308	-3.11<br>-13.26, 7.04	0.5398	7.90<br>-2.48, 18.28	0.1320	3.41<br>-6.83, 13.64	0.5052	-4.43<br>-25.75, 16.88	0.6769
**Instrument (New)**	0.04<br>-0.53, 0.60	0.8997	5.28<br>4.21, 6.35	<0.0001	-1.07<br>-3.01, 0.87	0.2744	-1.68<br>-3.70, 0.35	0.1048	-0.72<br>-5.81, 4.37	0.7771	-0.43<br>-2.13, 1.27	0.6120

Reference: Age = 75+, Sex = Female; Test order = Old instrument; Instrument = Old.

Table S3B (continued): Mixed effects models comparing ECG measures between the old and new instruments

	T Axis		P offset		P onset		Q offset		Q onset		T offset	
	**Estimate (95% CI)**	**p-value**	**Estimate (95% CI)**	**p-value**	**Estimate (95% CI)**	**p-value**	**Estimate (95% CI)**	**p-value**	**Estimate (95% CI)**	**p-value**	**Estimate (95% CI)**	**p-value**
**Intercept**	61.56<br>40.58, 82.55	<.0001	308.05<br>260.66, 355.43	<.0001	231.93<br>199.74, 264.13	<.0001	514.37<br>508.01, 520.73	<.0001	436.33<br>429.81, 442.85	<.0001	842.45<br>829.31, 855.59	<.0001
**45-54**	-18.04<br>-44.88, 8.80	0.1824	90.41<br>29.84, 150.98	0.0044	73.06<br>31.95, 114.16	0.0010	-3.43<br>-11.57, 4.72	0.4011	3.16<br>-5.19, 11.51	0.4495	0.07<br>-16.72, 16.86	0.9931
**55-64**	-16.34<br>-41.00, 8.33	0.1887	71.67<br>15.99, 127.35	0.0129	49.26<br>11.60, 86.91	0.0120	-3.01<br>-10.48, 4.47	0.4218	3.37<br>-4.29, 11.04	0.3796	11.99<br>-3.44, 27.43	0.1244
**65-74**	-10.76<br>-34.67, 13.14	0.3690	67.83<br>13.86, 121.80	0.0150	49.25<br>12.69, 85.81	0.0099	-1.99<br>-9.23, 5.26	0.5828	-0.76<br>-8.19, 6.67	0.8380	4.68<br>-10.28, 19.64	0.5313
**Sex (Male)**	-10.82<br>-28.90, 7.25	0.2337	-49.02<br>-89.82, -8.23	0.0197	-27.51<br>-55.09, 0.07	0.0505	1.74<br>-3.74, 7.22	0.5251	-7.43<br>-13.05, -1.82	0.0107	-6.42<br>-17.72, 4.89	0.2588
**Heart rate**	0.16<br>-0.63, 0.94	0.6881	-1.64<br>-3.41, 0.12	0.0673	-1.38<br>-2.57, -0.18	0.0256	0.10<br>-0.14, 0.34	0.3939	0.00<br>-0.25, 0.24	0.9852	-1.34<br>-1.83, -0.85	<.0001
**Test order (new first)**	-11.26<br>-29.30, 6.79	0.2151	23.54<br>-17.19, 64.28	0.2500	9.60<br>-17.88, 37.08	0.4813	3.89<br>-1.57, 9.36	0.1583	-0.04<br>-5.65, 5.56	0.9874	-2.78<br>-14.07, 8.51	0.6224
**Instrument (New)**	1.05<br>-0.70, 2.80	0.2349	11.03<br>7.15, 14.92	<.0001	-7.62<br>-10.52, -4.72	<.0001	6.69<br>5.09, 8.29	<.0001	1.31<br>-0.23, 2.85	0.0951	0.54<br>-1.98, 3.05	0.6705

Reference: Age = 75+, Sex = Female; Test order = Old instrument; Instrument = Old

Table S3C: Mixed effects models comparing log transformed ECG measures between the old and new instruments

	Ventricular Rate		QRS Duration		QT Interval		QTC Interval		P Axis		R Axis	
	**Estimate (95% CI)**	**p-value**	**Estimate (95% CI)**	**p-value**	**Estimate (95% CI)**	**p-value**	**Estimate (95% CI)**	**p-value**	**Estimate (95% CI)**	**p-value**	**Estimate (95% CI)**	**p-value**
**Intercept**	4.16<br>4.10, 4.22	<.0001	4.36<br>4.29, 4.43	<.0001	6.00<br>5.97, 6.03	<.0001	6.04<br>6.01, 6.07	<.0001	4.56<br>4.41, 4.71	<.0001	4.15<br>3.67, 4.63	<.0001
**45-54**	0.00<br>-0.07, 0.07	0.9596	-0.06<br>-0.15, 0.03	0.1752	0.00<br>-0.04, 0.03	0.8610	0.00<br>-0.04, 0.03	0.9340	-0.12<br>-0.31, 0.07	0.2077	0.28<br>-0.33, 0.89	0.3570
**55-64**	0.02<br>-0.05, 0.08	0.6485	-0.07<br>-0.15, 0.01	0.0925	0.02<br>-0.01, 0.05	0.2472	0.03<br>-0.00, 0.06	0.0689	0.02<br>-0.15, 0.19	0.8082	0.19<br>-0.37, 0.76	0.4905
**65-74**	0.03<br>-0.03, 0.10	0.3462	-0.01<br>-0.09, 0.07	0.8200	0.02<br>-0.02, 0.05	0.3574	0.03<br>-0.01, 0.06	0.1156	0.02<br>-0.14, 0.19	0.7795	0.12<br>-0.43, 0.66	0.6659
**Sex (Male)**	-0.04<br>-0.09, 0.01	0.0811	0.10<br>0.04, 0.16	0.0014	0.00<br>-0.02, 0.03	0.7247	-0.02<br>-0.04, 0.01	0.1385	0.00<br>-0.12, 0.12	0.9839	-0.20<br>-0.61, 0.21	0.3292
**Heart rate**	0.01<br>0.01, 0.01	<.0001	0.00<br>-0.00, 0.00	0.5528	0.00<br>-0.00, -0.00	<.0001	0.00<br>0.00, 0.00	<.0001	0.00<br>-0.00, 0.01	0.6913	0.00<br>-0.02, 0.02	0.9140
**Test order (new first)**	0.06<br>0.01, 0.11	0.0225	0.04<br>-0.02, 0.10	0.2274	-0.01<br>-0.03, 0.02	0.6034	0.02<br>-0.01, 0.04	0.1360	0.04<br>-0.08, 0.16	0.4776	0.14<br>-0.27, 0.55	0.4985
**Instrument (New)**	0.00<br>-0.01, 0.01	0.7371	0.06<br>0.05, 0.07	<.0001	-0.00<br>-0.01, 0.00	0.2809	-0.00<br>-0.01, 0.00	0.1096	-0.04<br>-0.15, 0.08	0.5226	-0.01<br>-0.03, 0.02	0.5786

Reference: Age = 75+, Sex = Female; Test order = Old instrument; Instrument = Old.

Percentage change: ventricular rate = 0.17%; QRS duration = 6.37%; QT interval = 0.25%, QTC interval = 0.39%, P axis = 3.67%, R axis = 0.67%

Table S3C (continued): Mixed effects models comparing log transformed ECG measures between the old and new instruments

	T Axis		P offset		P onset		Q offset		Q onset		T offset	
	Estimate (95% CI)	p-value	Estimate (95% CI)	p-value	Estimate (95% CI)	p-value	Estimate (95% CI)	p-value	Estimate (95% CI)	p-value	Estimate (95% CI)	p-value
**Intercept**	4.63<br>4.29, 4.98	<.0001	5.86<br>5.79, 5.92	<.0001	4.94<br>4.37, 5.51	<.0001	6.24<br>6.23, 6.26	<.0001	6.08<br>6.06, 6.09	<.0001	6.74<br>6.72, 6.75	<.0001
**45-54**	-0.18<br>-0.61, 0.26	0.4270	0.08<br>-0.00, 0.16	0.0629	0.93<br>0.20, 1.66	0.0139	-0.01<br>-0.02, 0.01	0.3832	0.01<br>-0.01, 0.03	0.4629	0.00<br>-0.02, 0.02	0.9937
**55-64**	-0.28<br>-0.68, 0.13	0.1747	0.08<br>0.01, 0.15	0.0356	0.61<br>-0.06, 1.28	0.0731	-0.01<br>-0.02, 0.01	0.4239	0.01<br>-0.01, 0.03	0.3743	0.01<br>-0.00, 0.03	0.1298
**65-74**	-0.08<br>-0.47, 0.32	0.6995	0.04<br>-0.03, 0.11	0.2519	0.73<br>0.08, 1.38	0.0290	-0.00<br>-0.02, 0.01	0.5715	0.00<br>-0.02, 0.02	0.8390	0.01<br>-0.01, 0.02	0.5306
**Sex (Male)**	-0.20<br>-0.49, 0.10	0.1824	-0.04<br>-0.09, 0.02	0.1891	-0.39<br>-0.88, 0.10	0.1150	0.00<br>-0.01, 0.01	0.5286	-0.02<br>-0.03, -0.00	0.0105	-0.01<br>-0.02, 0.01	0.2688
**Heart rate**	0.00<br>-0.01, 0.01	0.7977	0.00<br>-0.00, 0.00	0.9469	-0.03<br>-0.05, -0.01	0.0171	0.00<br>-0.00, 0.00	0.4095	0.00<br>-0.00, 0.00	0.9972	-0.00<br>-0.00, -0.00	<.0001
**Test order (new first)**	-0.05<br>-0.34, 0.25	0.7533	0.01<br>-0.04, 0.06	0.7070	0.26<br>-0.23, 0.75	0.2861	0.01<br>-0.00, 0.02	0.1649	0.00<br>-0.01, 0.01	0.9687	0.00<br>-0.02, 0.01	0.6324
**Instrument (New)**	0.00<br>-0.04, 0.05	0.8339	0.03<br>0.02, 0.04	<.0001	-0.03<br>-0.04, -0.02	<.0001	0.01<br>0.01, 0.02	<.0001	0.00<br>-0.00, 0.01	0.0979	0.00<br>-0.00, 0.00	0.6841

Reference: Age = 75+, Sex = Female; Test order = Old instrument; Instrument = Old.

Percentage change: T Axis = 0.46%, P offset = 3.21%, P onset = 2.95%, Q offset = 1.30%, Q onset = 0.30%, T offset = 0.06%

Table S4A: Covariance Parameter Estimates for Variability Analysis in carotid intima-media thickness

Covariance Parameter Estimates				
			Estimate	
**Cov Parm**	Subject	Group	**Right cIMT**	**Left cIMT**
**Var(1)**	ID		0.02203	0.02043
**Var(2)**	ID		0.02413	0.02082
**Corr(2,1)**	ID		0.9542	0.9421
**Residual**	ID	GROUP NEW	0.000329	0.000467
**Residual**	ID	GROUP OLD	0.000488	0.000428

Table S4B: Mixed effects models comparing carotid intima-media thickness between the old and new instruments

	**Right-cIMT (n=50)**			**Left-cIMT (n=49)***		
	**Estimate**	**95% CI**	**p-value**	**Estimate**	**95% CI**	**p-value**
**Intercept**	0.93	0.83, 1.04	<.0001	0.96	0.86, 1.05	<.0001
**45-54**	-0.32	-0.44, -0.19	<.0001	-0.29	-0.41, -0.17	<.0001
**55-64**	-0.21	-0.33, -0.10	0.0007	-0.20	-0.31, -0.09	0.0008
**65-74**	-0.15	-0.26, -0.03	0.0128	-0.22	-0.33, -0.11	0.0003
**Sex (Male)**	-0.05	-0.13, 0.04	0.2779	-0.004	-0.08, 0.08	0.9143
**Test order (new first)**	-0.07	-0.15, 0.02	0.1171	-0.03	-0.11, 0.05	0.4252
**Instrument (New)**	-0.02	-0.04, -0.01	0.0012	-0.02	-0.04, -0.01	0.0048

Reference: Age = 75+, Sex = Female; Test order = Old instrument; Instrument = Old

*One individual (ID: 12) dropped from analysis as the residual was >4 units.

Table S4C: Mixed effects models comparing log transformed carotid intima-media thickness between the old and new instruments

	Right-cIMT (n=50)			Left-cIMT (n=49)*		
	**Estimate**	**95% CI**	**p-value**	**Estimate**	**95% CI**	**p-value**
**Intercept**	-0.06	-0.19, 0.07	0.3352	-0.06	-0.18, 0.07	0.3737
**45-54**	-0.45	-0.61, -0.29	<0.0001	-0.37	-0.52, -0.21	<0.0001
**55-64**	-0.30	-0.45, -0.14	0.0003	-0.24	-0.39, -0.10	0.0016
**65-74**	-0.20	-0.35, -0.05	0.0087	-0.27	-0.41, -0.13	0.0005
**Sex (Male)**	-0.07	-0.19, 0.04	0.1941	-0.02	-0.12, 0.09	0.7679
**Test order (new first)**	-0.08	-0.19, 0.03	0.1562	-0.03	-0.14, 0.07	0.5409
**Instrument (New)**	-0.03	-0.05, -0.01	0.0016	-0.03	-0.05, -0.01	0.0041

Reference: Age = 75+, Sex = Female; Test order = Old instrument; Instrument = Old.
Percentage change: FEV1 = 3.23% (1.29%, 5.12%); FVC = 2.95% (0.99%, 4.87%)
*One individual (ID: 12) dropped from analysis as the residual was >4 units.

Table S5A: Covariance Parameter Estimates for Variability Analysis in spirometer measures

**Covariance Parameter Estimates**				
			**Estimate**	
**Cov Parm**	Subject	Group	**FEV1**	**FVC**
**Var(1)**	ID		0.2523	0.3286
**Var(2)**	ID		0.2667	0.3557
**Corr(2,1)**	ID		0.9983	0.991
**Residual**	ID	GROUP NEW	0.008136	0.01413
**Residual**	ID	GROUP OLD	0.01082	0.0113

Table S5B: Mixed effects models comparing FEV1 and FVC between the old and new instruments

	FEV1			FVC		
	**Estimate**	**95% CI**	**p-value**	**Estimate**	**95% CI**	**p-value**
**Intercept**	1.97	1.63, 2.31	<.0001	2.80	2.41, 3.19	<.0001
**45-54**	0.69	0.27, 1.10	0.0017	0.68	0.21, 1.16	0.0059
**55-64**	0.59	0.18, 1.01	0.0060	0.61	0.13, 1.08	0.0137
**65-74**	0.06	-0.33, 0.45	0.7705	0.06	-0.39, 0.50	0.8017
**Sex (Male)**	0.66	0.27, 1.06	0.0013	0.70	0.26, 1.15	0.0028
**Height (cm)**	0.02	0.00, 0.04	0.0778	0.04	0.01, 0.06	0.0049
**Instrument (New)**	0.05	0.03, 0.08	0.0001	0.05	0.02, 0.09	0.0059

Reference: Age = 75+, Sex = Female; Instrument = Old.

Table S5C: Mixed effects models comparing log transformed FEV1 and FVC between the old and new instruments

	FEV1			FVC		
	**OR**	**95% CI**	**p-value**	**OR**	**95% CI**	**p-value**
**Intercept**	-0.64	-2.06, 0.79	0.3707	-0.76	-2.02, 0.50	0.2290
**45-54**	0.25	0.09, 0.42	0.0028	0.20	0.05, 0.34	0.0082
**55-64**	0.21	0.05, 0.37	0.0114	0.18	0.04, 0.32	0.0157
**65-74**	0.01	-0.14, 0.16	0.9173	0.02	-0.11, 0.15	0.7624
**Sex (Male)**	0.25	0.10, 0.41	0.0016	0.21	0.08, 0.35	0.0028
**Height (cm)**	0.01	-0.00, 0.02	0.0809	0.01	0.00, 0.02	0.0095
**Instrument (New)**	0.02	0.01, 0.03	<0.0001	0.02	0.01, 0.03	0.0016

Reference: Age = 75+, Sex = Female; Instrument = Old.
Percentage change: FEV1 = 2.37% (1.35%, 3.40%); FVC = 1.74% (0.69%, 2.80%)

Table S6A: Covariance Parameter Estimates for Variability Analysis in audiometer measures

**Covariance Parameter Estimates**									
			**Estimate**						
**Cov Parm**	Subject	Group	**Frequency 500 Hz**	**Frequency 1000 Hz**	**Frequency 2000 Hz**	**Frequency 3000 Hz**	**Frequency 4000 Hz**	**Frequency 6000 Hz**	**Frequency 8000 Hz**
**Var(1)**	ID		158.40	203.19	243.60	268.40	316.25	383.25	344.69
**Var(2)**	ID		153.41	169.51	273.57	262.62	258.70	294.21	299.67
**Corr(2,1)**	ID		1	1	1	1	1	1	1
**Residual**	ID	GROUP NEW	83.4909	74.9724	71.8615	77.0371	57.2249	96.1679	107.02
**Residual**	ID	GROUP OLD	76.5303	70.6989	60.4587	91.1711	67.3299	139.35	125.74

Table S6B: Mixed effects models comparing audiometer measures between the old and new instruments

	Frequency 500 Hz		Frequency 1000 Hz		Frequency 2000 Hz		Frequency 3000 Hz		Frequency 4000 Hz		Frequency 6000 Hz		Frequency 8000 Hz	
	**Estimate (95% CI)**	**p-value**	**Estimate (95% CI)**	**p-value**	**Estimate (95% CI)**	**p-value**	**Estimate (95% CI)**	**p-value**	**Estimate (95% CI)**	**p-value**	**Estimate (95% CI)**	**p-value**	**Estimate (95% CI)**	**p-value**
**Intercept**	31.71<br>22.75, 40.67	<.0001	30.01<br>20.51, 39.51	<.0001	31.77<br>20.58, 42.95	<.0001	36.17<br>24.74, 47.59	<.0001	38.63<br>27.17, 50.10	<.0001	48.60<br>35.82, 61.38	<.0001	62.15<br>49.56, 74.74	<.0001
**45-54**	-11.27<br>-22.19, -0.35	0.0434	-14.51<br>-26.15, -2.86	0.0107	-22.16<br>-35.77, -8.55	0.0020	-28.63<br>-42.58, -14.68	0.0002	-25.27<br>-39.34, -11.19	0.0008	-28.76<br>-44.41, -13.12	0.0006	-37.97<br>-53.37, -22.57	<.0001
**55-64**	-7.91<br>-18.30, 2.47	0.1318	-8.01<br>-19.09, 3.06	0.1481	-13.18<br>-26.13, -0.23	0.0462	-18.68<br>-31.95, -5.42	0.0068	-22.12<br>-35.52, -8.72	0.0018	-25.33<br>-40.21, -10.45	0.0013	-36.35<br>-51.00, -21.70	<.0001
**65-74**	-3.23<br>-13.37, 6.90	0.5237	-3.67<br>-14.48, 7.15	0.558	-3.40<br>-16.05, 9.24	0.5901	-7.03<br>-19.98, 5.93	0.2799	-6.85<br>-19.92, 6.21	0.2961	-5.25<br>-19.78, 9.27	0.4697	-11.50<br>-25.80, 2.80	0.1121
**Sex (Male)**	0.34<br>-7.31, 7.99	0.9292	2.91<br>-5.25, 11.06	0.4757	9.53<br>-0.01, 19.06	0.0503	15.41<br>5.64, 25.18	0.0027	17.79<br>7.93, 27.65	0.0007	17.59<br>6.65, 28.53	0.0023	10.25<br>-0.54, 21.03	0.0620
**Test order (new first)**	-0.86<br>-8.45, 6.73	0.8208	-0.35<br>-8.45, 7.75	0.8721	-0.32<br>-9.79, 9.15	0.9461	3.84<br>-5.85, 13.54	0.4284	1.72<br>-8.07, 11.51	0.7255	2.69<br>-8.17, 13.55	0.6201	2.55<br>-8.16, 13.25	0.6338
**Instrument (New)**	-5.35<br>-7.85, -2.85	<.0001	-6.35<br>-8.76, -3.94	<.0001	-5.16<br>-7.46, -2.86	<.0001	-4.50<br>-7.07, -1.93	0.0007	-5.60<br>-7.87, -3.33	<.0001	-5.81<br>-8.98, -2.64	0.0004	-5.72<br>-8.81, -2.62	0.0004

Reference: Age = 75+, Sex = Female; Test order = Old instrument; Instrument = Old.

Table S6C: Mixed effects models comparing log transformed audiometer measures between the old and new instruments

	Frequency 500 Hz		Frequency 1000 Hz		Frequency 2000 Hz		Frequency 3000 Hz		Frequency 4000 Hz	
	**Estimate (95% CI)**	**p-value**	**Estimate (95% CI)**	**p-value**	**Estimate (95% CI)**	**p-value**	**Estimate (95% CI)**	**p-value**	**Estimate (95% CI)**	**p-value**
**Intercept**	3.43 (3.17, 3.70)	<0.0001	3.29 (2.96, 3.62)	<0.0001	3.41 (2.02, 4.79)	<0.0001	3.40 (3.03, 3.76)	<0.0001	3.46 (3.13, 3.80)	<0.0001
**45-54**	-0.28 (-0.61, 0.05)	0.0913	-0.60 (-1.01, -0.19)	0.0050	-1.93 (-3.63, -0.23)	0.0272	-1.01 (-1.46, -0.56)	<0.0001	-0.61 (-1.02, -0.20)	0.0046
**55-64**	-0.36 (-0.67, -0.05)	0.0251	-0.36 (-0.75, 0.03)	0.0710	-1.32 (-2.94, 0.30)	0.1069	-0.65 (-1.07, -0.22)	0.0038	-0.64 (-1.03, -0.25)	0.0018
**65-74**	-0.20 (-0.50, 0.10)	0.1916	-0.24 (-0.62, 0.14)	0.2108	-0.63 (-2.21, 0.95)	0.4276	-0.29 (-0.70, 0.13)	0.1743	-0.29 (-0.66, 0.09)	0.1364
**Sex (Male)**	0.03 (-0.20, 0.26)	0.7730	0.21 (-0.07, 0.50)	0.1411	1.18 (-0.02, 2.37)	0.0528	0.48 (0.17, 0.80)	0.0033	0.53 (0.24, 0.82)	0.0005
**Test order (new first)**	-0.17 (-0.40, 0.06)	0.1412	-0.09 (-0.28, 0.19)	0.5177	-0.90 (-2.08, 0.28)	0.1321	0.17 (-0.15, 0.48)	0.2887	0.04 (-0.24, 0.33)	0.7766
**Instrument (New)**	-0.33 (-0.44, -0.22)	<0.0001	-0.44 (-0.58, -0.30)	<0.0001	-0.48 (-0.82, -0.15)	0.0047	-0.21 (-0.30, -0.11)	<0.0001	-0.30 (-0.40, -0.19)	<0.0001

Reference: Age = 75+, Sex = Female; Test order = Old instrument; Instrument = Old.

Percentage change: 500 Hz = 28.05%; 1000 Hz = 35.75%; 2000 Hz = 38.6%, 3000 Hz = 18.67%, 4000 Hz = 25.66%

Table S6C (continued): Mixed effects models comparing log transformed audiometer measures between the old and new instruments

	Frequency 6000 Hz		Frequency 8000 Hz	
	**Estimate (95% CI)**	**p-value**	**Estimate (95% CI)**	**p-value**
**Intercept**	3.80 (3.47, 4.13)	<0.0001	4.09 (3.78, 4.41)	<0.0001
**45-54**	-0.78 (-1.18, -0.37)	0.0004	-0.74 (-1.13, -0.35)	0.0004
**55-64**	-0.71 (-1.10, -0.33)	0.0005	-0.86 (-1.23, -0.49)	<0.0001
**65-74**	-0.21 (-0.59, 0.17)	0.2648	-0.28 (-0.64, 0.08)	0.1241
**Sex (Male)**	0.54 (0.26, 0.83)	0.0004	0.21 (-0.06, 0.48)	0.1315
**Test order (new first)**	-0.12 (-0.40, 0.17)	0.4120	-0.09 (-0.36, 0.18)	0.5183
**Instrument (New)**	-0.20 (-0.32, -0.08)	0.0012	-0.27 (-0.40, -0.15)	<0.0001

Reference: Age = 75+, Sex = Female; Test order = Old instrument; Instrument = Old.

Percentage change: 6000 Hz = 18.09%; 8000 Hz = 23.77%

Table S7A: Covariance Parameter Estimates for Variability Analysis in tonometer measures

**Covariance Parameter Estimates**								
			**Estimate**					
**Cov Parm**	Subject	Group	**Corneal-Compensated IOP Right-Eye**	**Corneal-Compensated IOP Left-Eye**	**Intraocular Pressure Right-Eye**	**Intraocular Pressure Left-Eye**	**Corneal Hysteresis Right-Eye**	**Corneal Hysteresis Left-Eye**
**Var(1)**	ID		12.8483	12.5469	17.4155	17.2678	1.0282	0.6372
**Var(2)**	ID		9.8151	10.3332	12.7516	13.818	1.2872	1.0453
**Corr(2,1)**	ID		0.9148	0.9739	0.9562	0.9897	0.9129	1
**Residual**	ID	GROUP NEW	2.2536	2.0382	1.701	1.5882	0.3105	0.4851
**Residual**	ID	GROUP OLD	3.0225	2.6253	2.0493	1.4879	0.9174	1.2826

Table S7B: Mixed effects models comparing tonometry measures between the old and new instruments

	Corneal-Compensated IOP Right-Eye		Corneal-Compensated IOP Left-Eye		Intraocular Pressure Right-Eye		Intraocular Pressure Left-Eye		Corneal Hysteresis Right-Eye		Corneal Hysteresis Left-Eye	
	Estimate (95% CI)	p-value	Estimate (95% CI)	p-value	Estimate (95% CI)	p-value	Estimate (95% CI)	p-value	Estimate (95% CI)	p-value	Estimate (95% CI)	p-value
**Intercept**	16.87<br>14.67, 19.07	<.0001	16.38<br>14.13, 18.62	<.0001	15.74<br>13.30, 18.18	<.0001	15.41<br>12.91, 17.90	<.0001	9.66<br>8.92, 10.39	<.0001	10.04<br>9.40, 10.68	<.0001
**45-54**	2.18<br>-0.52, 4.88	0.1106	2.55<br>-0.20, 5.31	0.0687	3.50<br>0.50, 6.49	0.0231	2.85<br>-0.20, 5.89	0.0666	0.74<br>-0.13, 1.61	0.0934	0.34<br>-0.40, 1.08	0.3576
**55-64**	-0.35<br>-2.92, 2.22	0.7842	-0.22<br>-2.84, 2.41	0.8692	-0.53<br>-3.38, 2.31	0.708	0.29<br>-2.61, 3.19	0.8393	0.56<br>-0.27, 1.39	0.1823	0.32<br>-0.38, 1.02	0.3677
**65-74**	1.58<br>-0.93, 4.09	0.2104	1.50<br>-1.06, 4.06	0.243	1.93<br>-0.85, 4.71	0.169	1.19<br>-1.65, 4.02	0.4033	0.19<br>-0.62, 1.00	0.6331	-0.07<br>-0.75, 0.62	0.8485
**Sex (Male)**	-0.02<br>-1.92, 1.87	0.9799	0.27<br>-1.66, 2.20	0.7776	-0.79<br>-2.89, 1.30	0.4502	-0.39<br>-2.52, 1.75	0.7180	-0.87<br>-1.48, -0.26	0.0064	-0.79<br>-1.30, -0.27	0.0035
**Test order (new first)**	-0.34<br>-2.22, 1.54	0.7168	-0.92<br>-2.84, 1.00	0.3394	-0.63<br>-2.71, 1.46	0.548	-1.07<br>-3.19, 1.05	0.3128	0.02<br>-0.58, 0.63	0.9367	-0.29<br>-0.80, 0.22	0.2544
**Instrument (New)**	-1.10<br>-1.66, -0.54	0.0002	-0.83<br>-1.26, -0.40	0.0003	-0.37<br>-0.86, 0.11	0.1294	-0.33<br>-0.68, 0.03	0.0688	0.67<br>0.44, 0.89	<.0001	0.54<br>0.31, 0.76	<.0001

Reference: Age = 75+, Sex = Female; Test order = Old instrument; Instrument = Old.

Table S7C: Mixed effects models comparing log transformed tonometry measures between the old and new instruments

	Corneal-Compensated IOP Right-Eye		Corneal-Compensated IOP Left-Eye		Intraocular Pressure Right-Eye		Intraocular Pressure Left-Eye		Corneal Hysteresis Right-Eye		Corneal Hysteresis Left-Eye	
	**Estimate (95% CI)**	**p-value**	**Estimate (95% CI)**	**p-value**	**Estimate (95% CI)**	**p-value**	**Estimate (95% CI)**	**p-value**	**Estimate (95% CI)**	**p-value**	**Estimate (95% CI)**	**p-value**
**Intercept**	2.81 (2.69, 2.94)	<0.0001	2.77 (2.64, 2.91)	<0.0001	2.72 (2.57, 2.87)	<0.0001	2.69 (2.53, 2.85)	<0.0001	2.26 (2.18, 2.33)	<0.0001	2.29 (2.23, 2.36)	<0.0001
**45-54**	0.11 (-0.05, 0.27)	0.1609	0.16 (-0.00, 0.32)	0.0529	0.22 (0.04, 0.40)	0.0207	0.19 (-0.01, 0.38)	0.0630	0.07 (-0.02, 0.16)	0.1122	0.03 (-0.04, 0.10)	0.3765
**55-64**	-0.02 (-0.17, 0.13)	0.8049	-0.01 (-0.16, 0.15)	0.9350	-0.03 (-0.21, 0.14)	0.7055	0.04 (-0.15, 0.22)	0.6859	0.05 (-0.03, 0.14)	0.2028	0.03 (-0.04, 0.10)	0.3883
**65-74**	0.08 (-0.07, 0.22)	0.2940	0.08 (-0.07, 0.23)	0.3074	0.13 (-0.04, 0.30)	0.1360	0.07 (-0.11, 0.25)	0.4553	0.02 (-0.06, 0.10)	0.6473	-0.01 (-0.07, 0.06)	0.8257
**Sex (Male)**	-0.01 (-0.12, 0.10)	0.9061	0.02 (-0.09, 0.14)	0.6647	-0.05 (-0.18, 0.08)	0.4592	-0.01 (-0.15, 0.12)	0.8467	-0.08 (-0.15, -0.02)	0.0072	-0.08 (-0.13, -0.03)	0.0030
**Test order (new first)**	-0.02 (-0.13, 0.09)	0.7199	-0.06 (-0.17, 0.05)	0.2677	-0.03 (-0.16, 0.09)	0.5941	-0.06 (-0.20, 0.07)	0.3679	0.00 (-0.06, 0.06)	0.9839	-0.03 (-0.08, 0.02)	0.2084
**Instrument (New)**	-0.07 (-0.11, -0.04)	0.0001	-0.06 (-0.08, -0.03)	<0.0001	-0.03 (-0.07, 0.00)	0.0551	-0.03 (-0.06, -0.01)	0.0148	0.07 (0.05, 0.10)	<0.0001	0.06 (0.04, 0.09)	<0.0001

Reference: Age = 75+, Sex = Female; Test order = Old instrument; Instrument = Old.

Percentage change: IOPcc (right-eye) = -6.76% (-3.92, -10.42); IOPcc (left-eye) = -5.82% (-2.96, -7.69); IOP (right-eye) = -2.96% (-6.76, -1.00); IOP (left-eye) = -2.96% (-5.82, -1.00); Corneal Hysteresis (right-eye) = 7.25% (5.13, 10.52); Corneal Hysteresis (left-eye) = 6.18% (4.08, 9.42)

Table S8A: Covariance Parameter Estimates for Variability Analysis

Covariance Parameter Estimates			
**Cov Parm**	Subject	Group	**Estimate**
**Var(1)**	ID		54.4197
**Var(2)**	ID		42.9637
**Corr(2,1)**	ID		0.923
**Residual**	ID	GROUP NEW	4.5039
**Residual**	ID	GROUP OLD	5.829

Table S8B: Mixed effects model comparing grip strength between the old and new instruments

	Grip Strength		
	**Estimate**	**95% CI**	**p-value**
**Intercept**	17.79	13.29, 22.28	<.0001
**45-54**	9.56	4.04, 15.09	0.0011
**55-64**	7.92	2.67, 13.17	0.0040
**65-74**	4.31	-0.82, 9.43	0.0975
**Sex (Male)**	16.91	13.05, 20.78	<.0001
**Test order (new first)**	3.39	-0.45, 7.23	0.0821
**Instrument (New)**	1.31	0.34, 2.27	0.0090

Reference: Age = 75+, Sex = Female; Test order = Old instrument; Instrument = Old

Table S8C: Mixed effects model comparing log transformed grip strength between the old and new instruments

	Grip Strength		
	**Estimate**	**95% CI**	**p-value**
**Intercept**	2.98	2.85, 3.11	<0.0001
**45-54**	0.29	0.13, 0.45	0.0006
**55-64**	0.24	0.09, 0.39	0.0027
**65-74**	0.17	0.02, 0.31	0.0263
**Sex (Male)**	0.52	0.41, 0.63	<0.0001
**Test order (new first)**	0.07	-0.04, 0.18	0.2259
**Instrument (New)**	0.03	0.00, 0.06	0.0220

Reference: Age = 75+, Sex = Female; Test order = Old instrument; Instrument = Old.

Percentage change: 3.35% (0.50, 6.28)

SBP

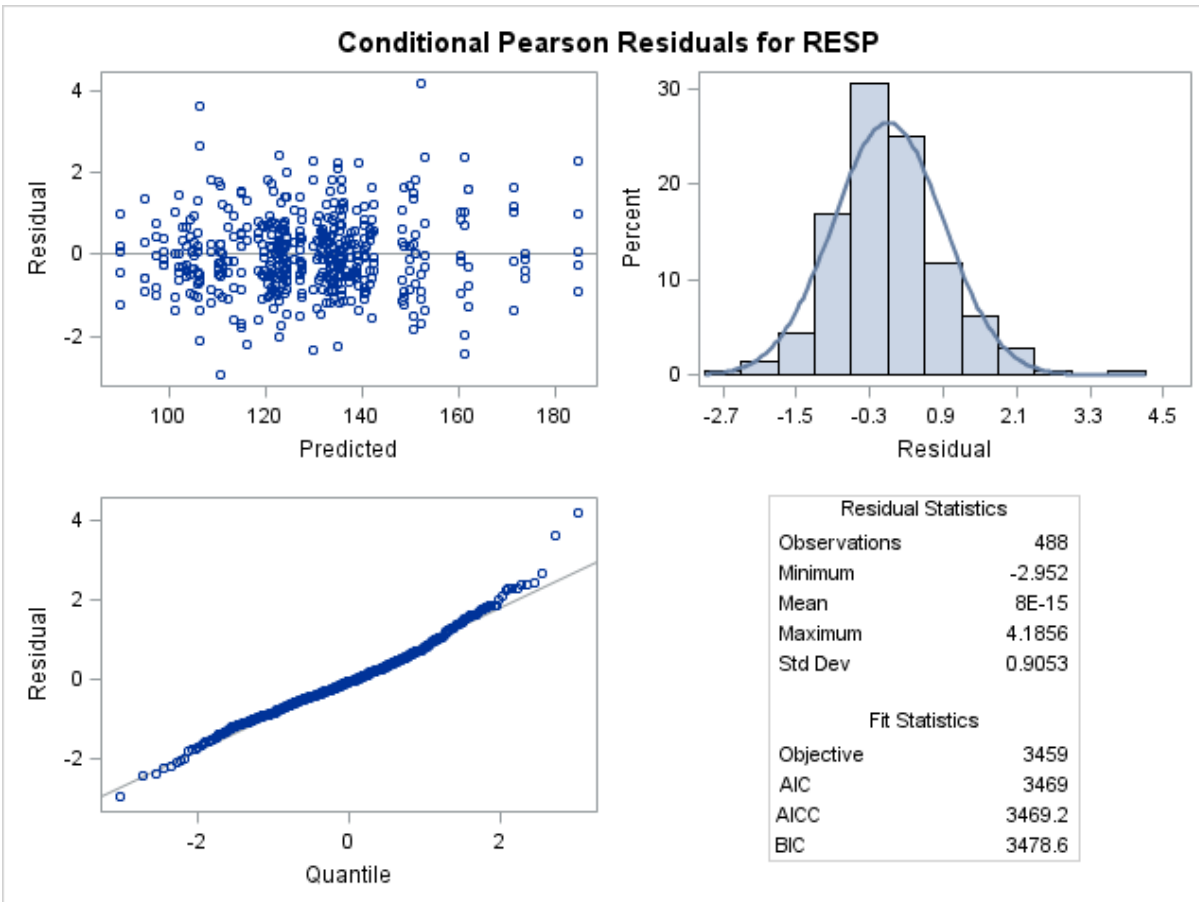

DBP

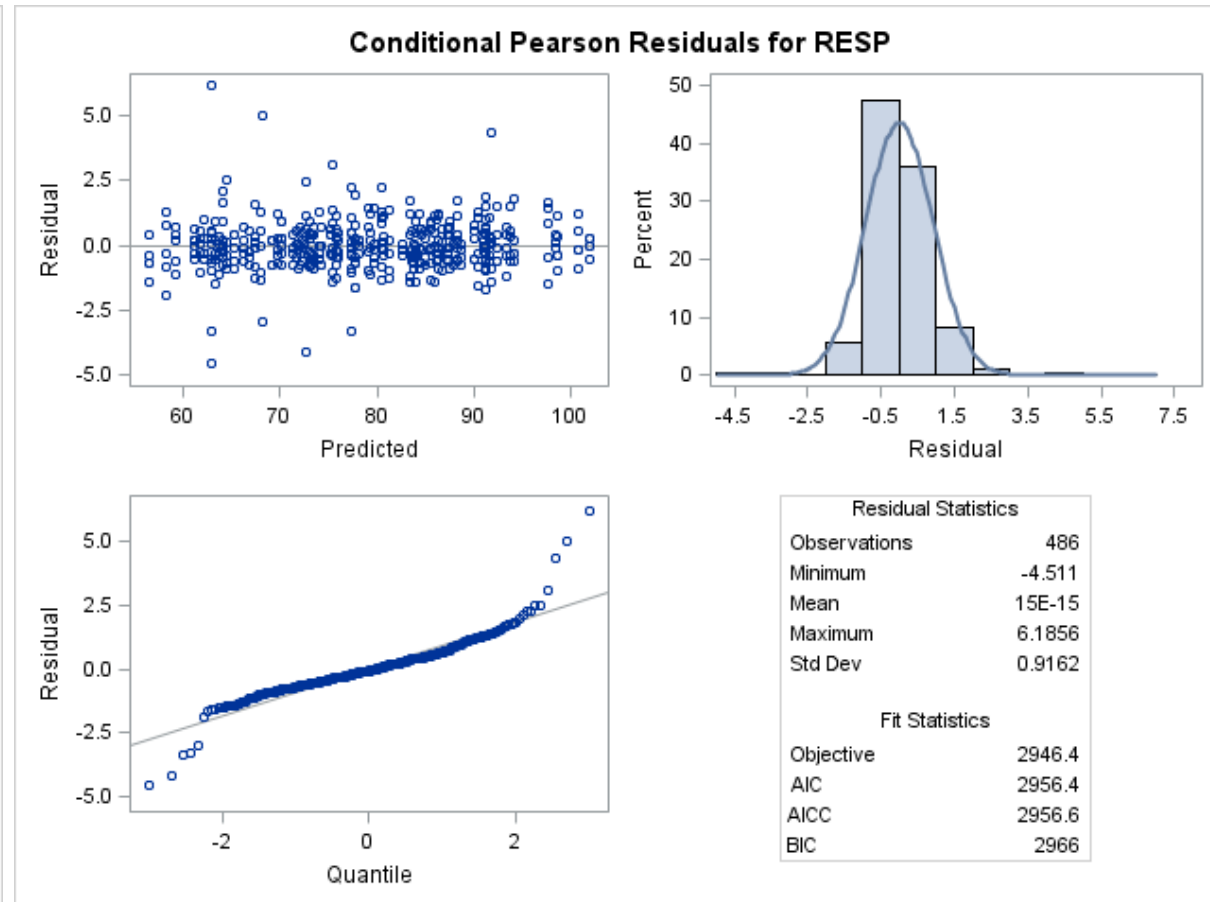

PP

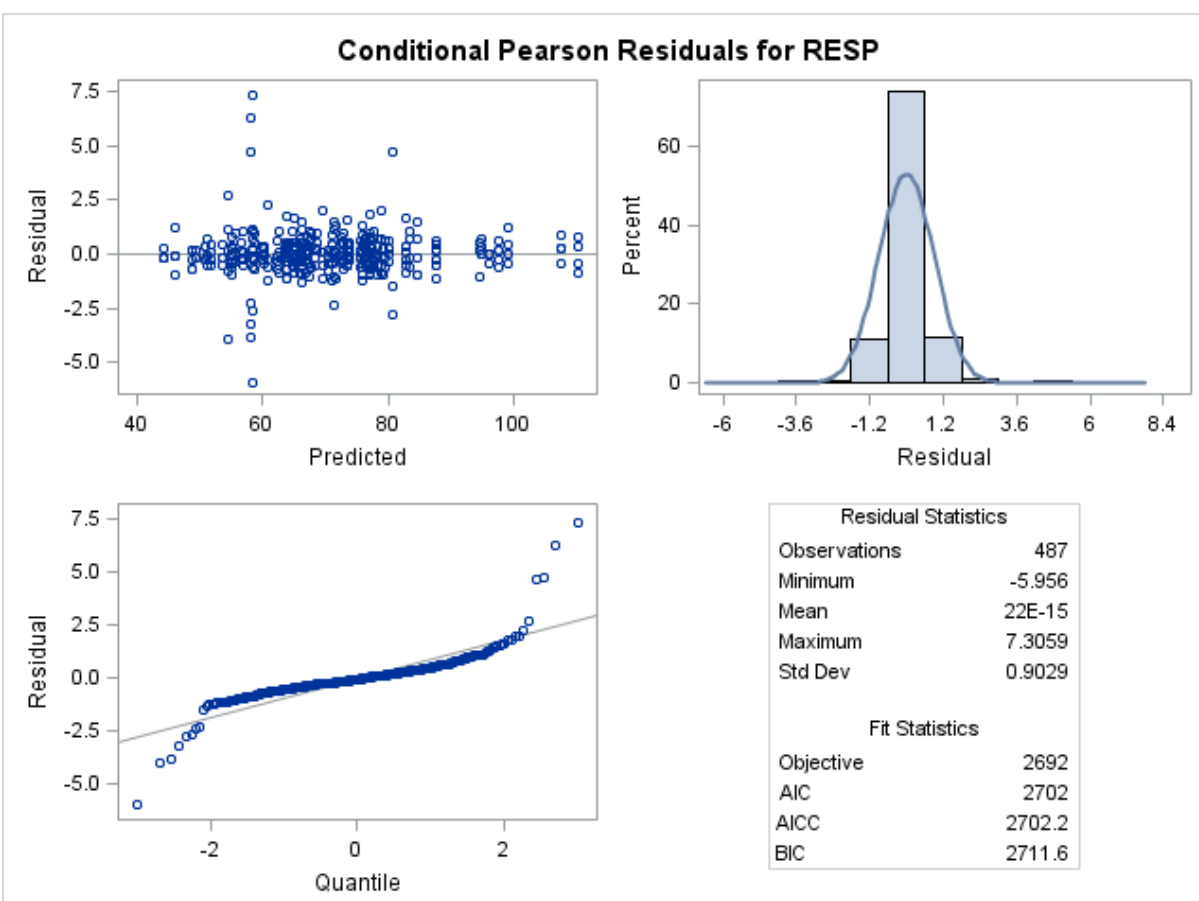

Figures S8: Diagnostic plots of Pearson residuals for the mixed model of SBP, DBP, and pulse rate

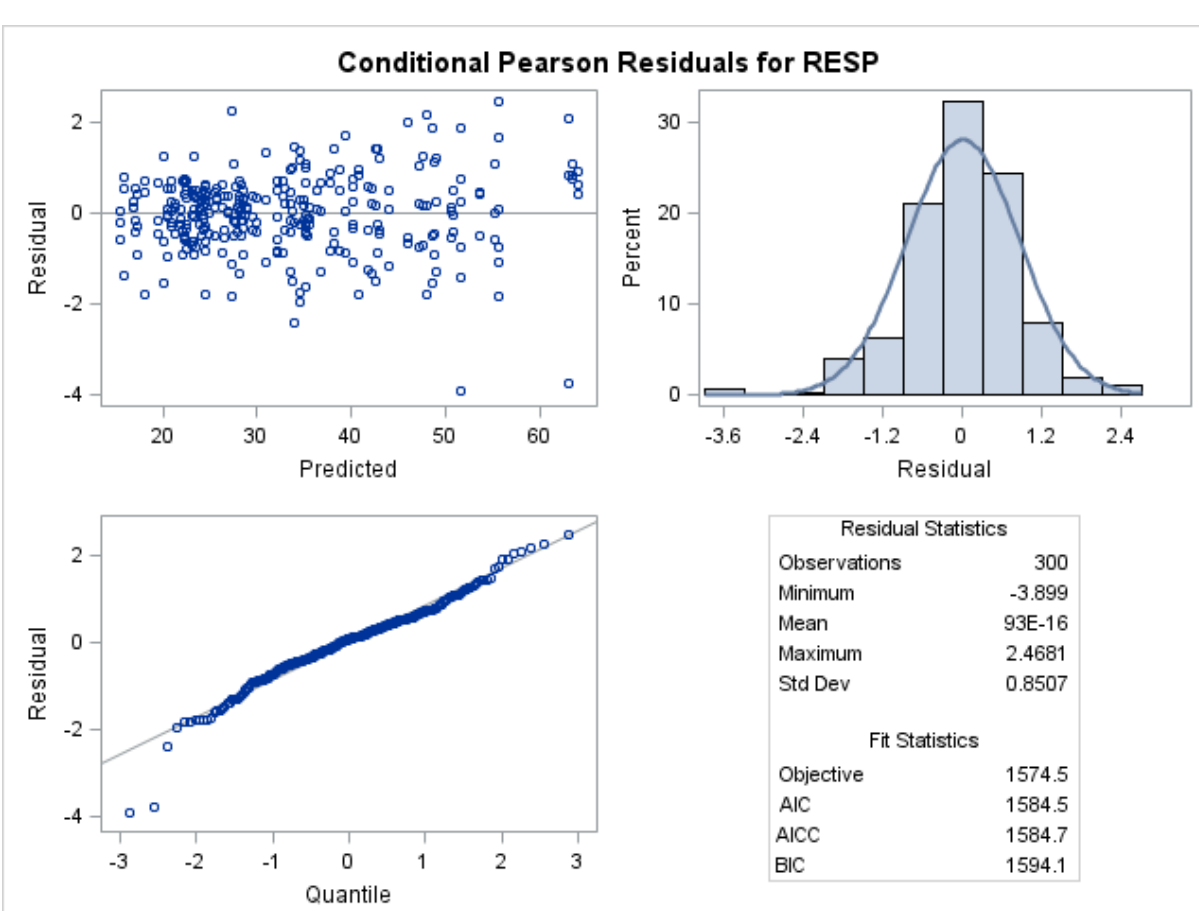

Figure S9: Diagnostic plots of Pearson residuals for the mixed model of grip strength

Frequency 500 Hz

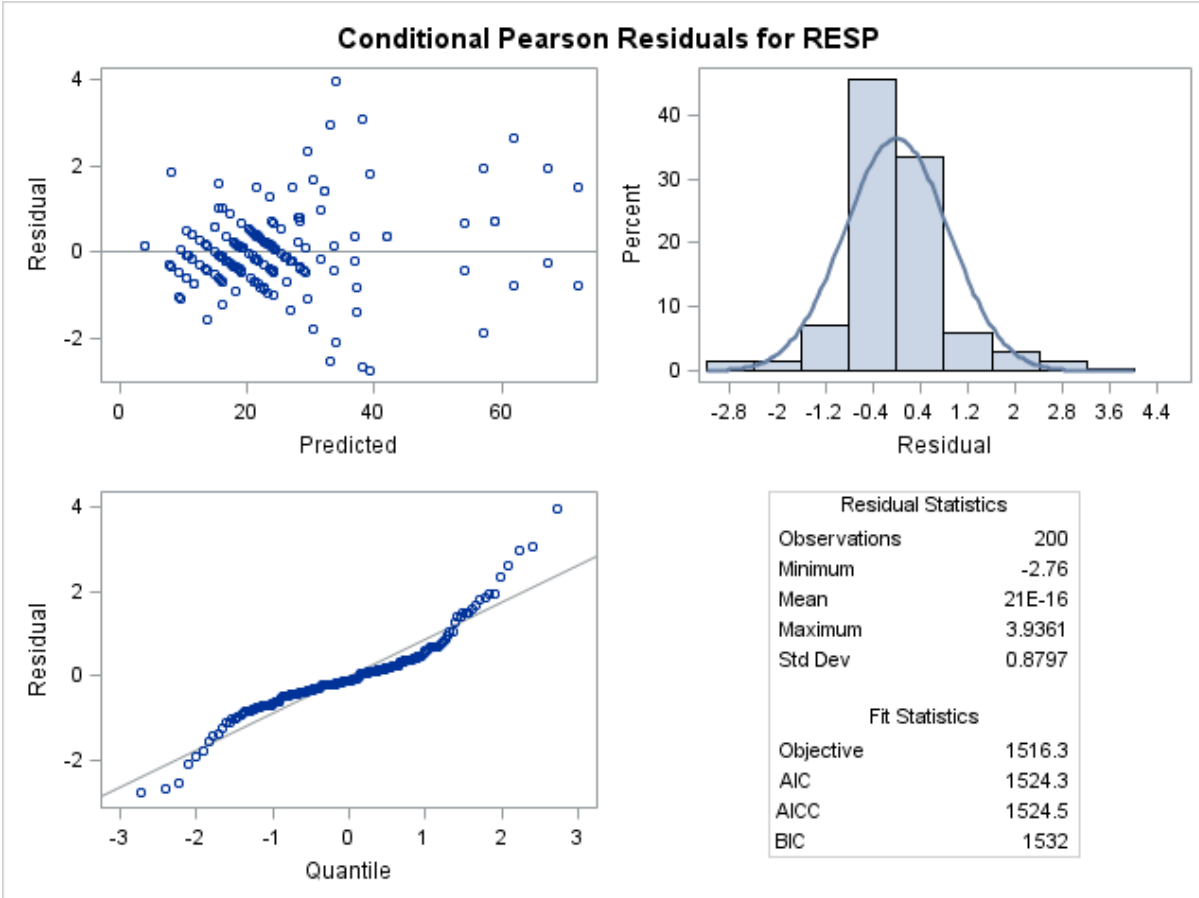

Frequency 1000 Hz

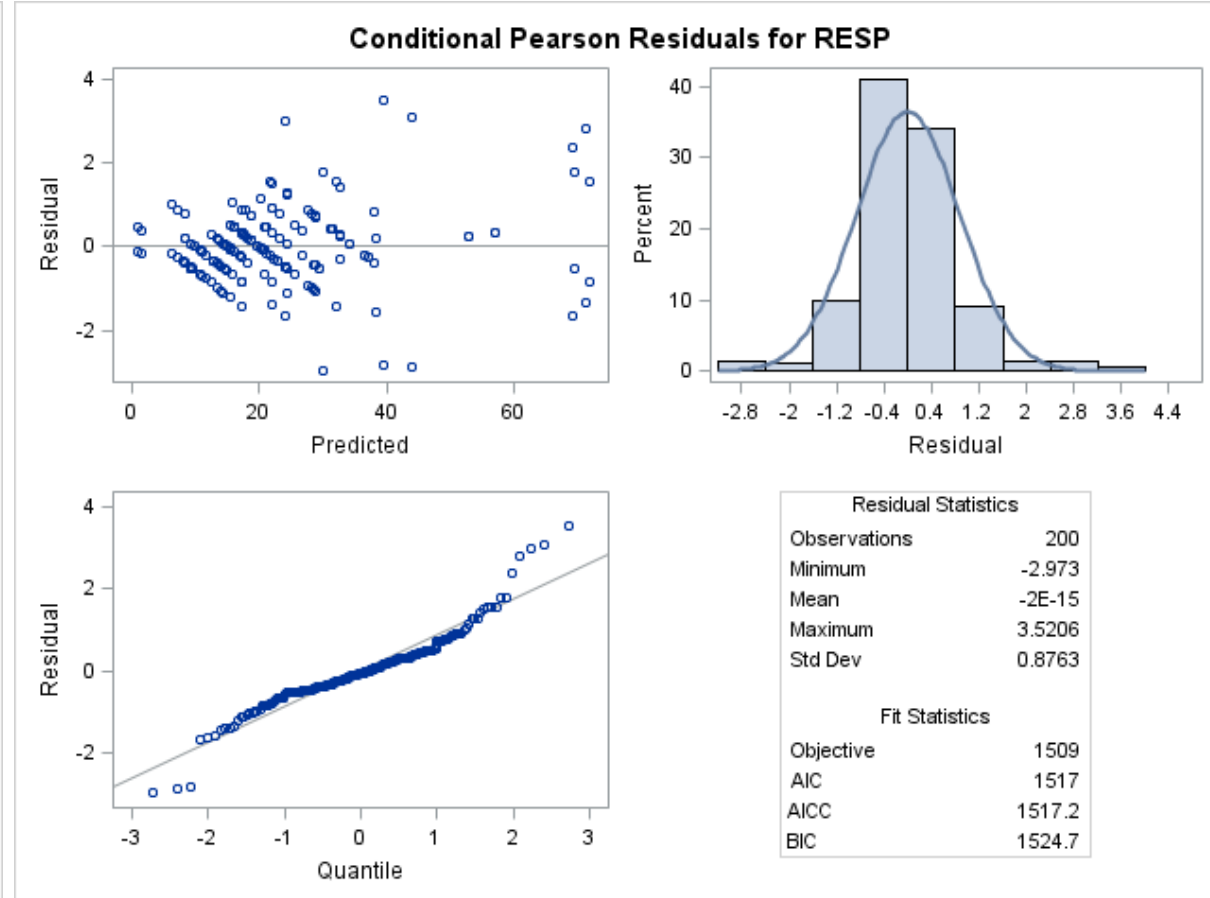

Frequency 2000 Hz

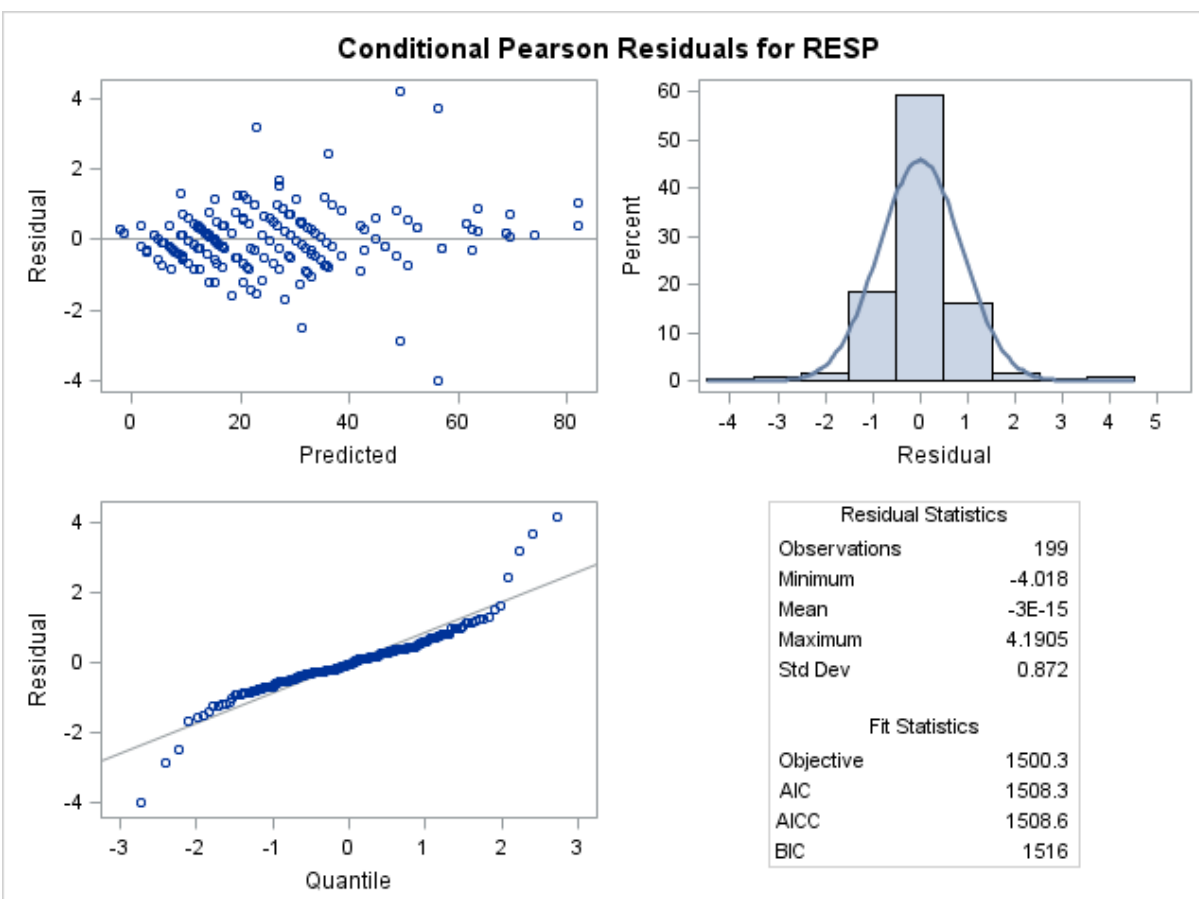

Frequency 3000 Hz

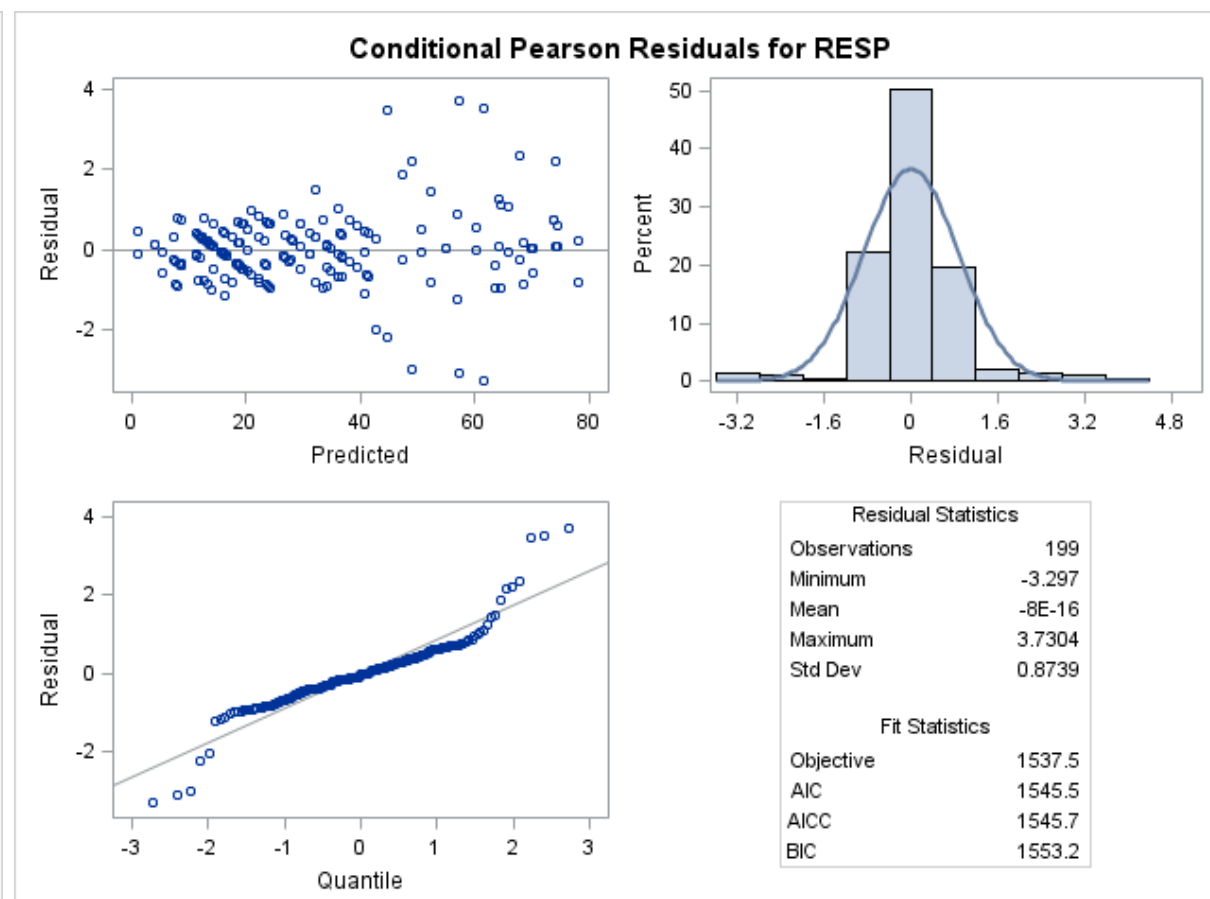

Frequency 4000 Hz

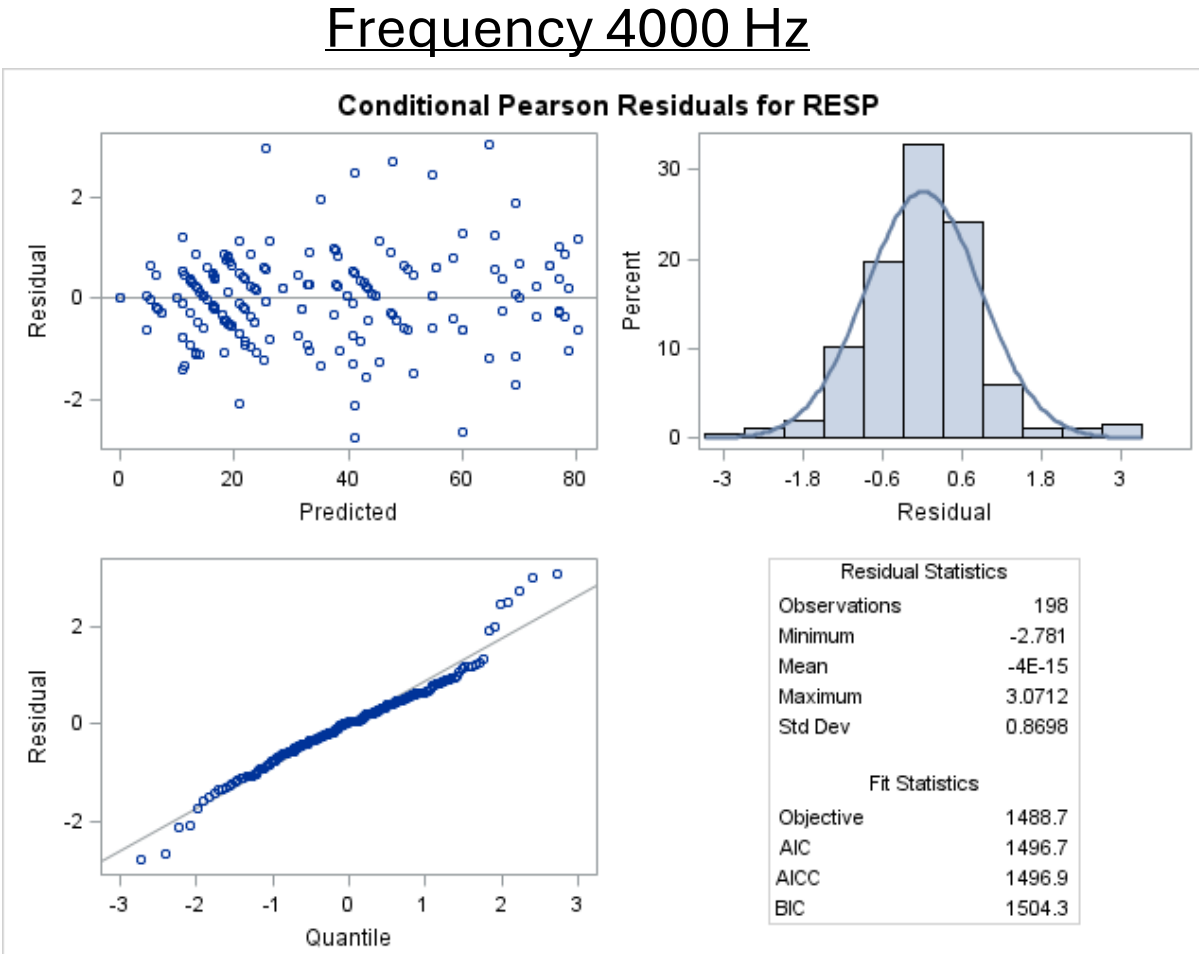

Frequency 6000 Hz

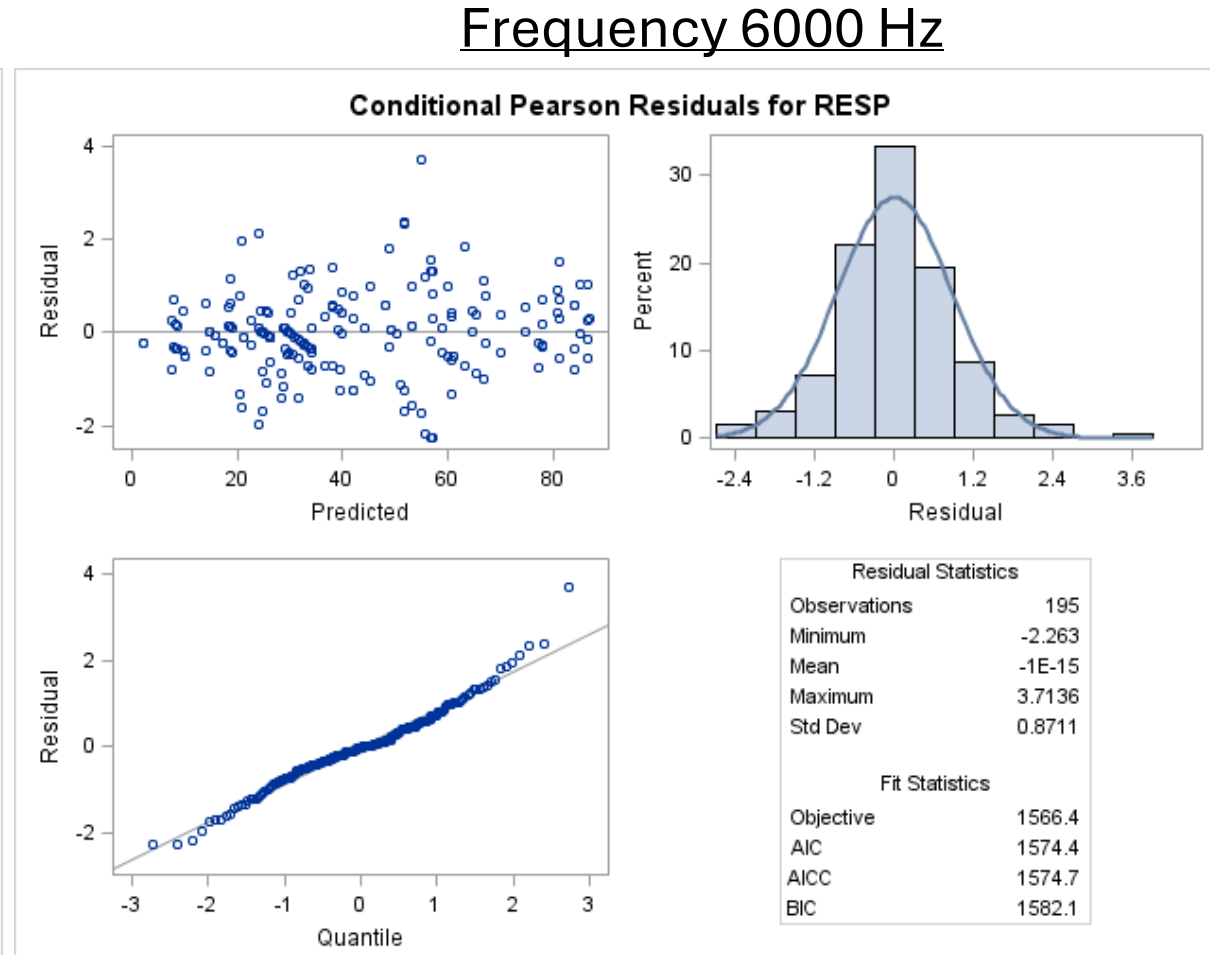

Frequency 8000 Hz

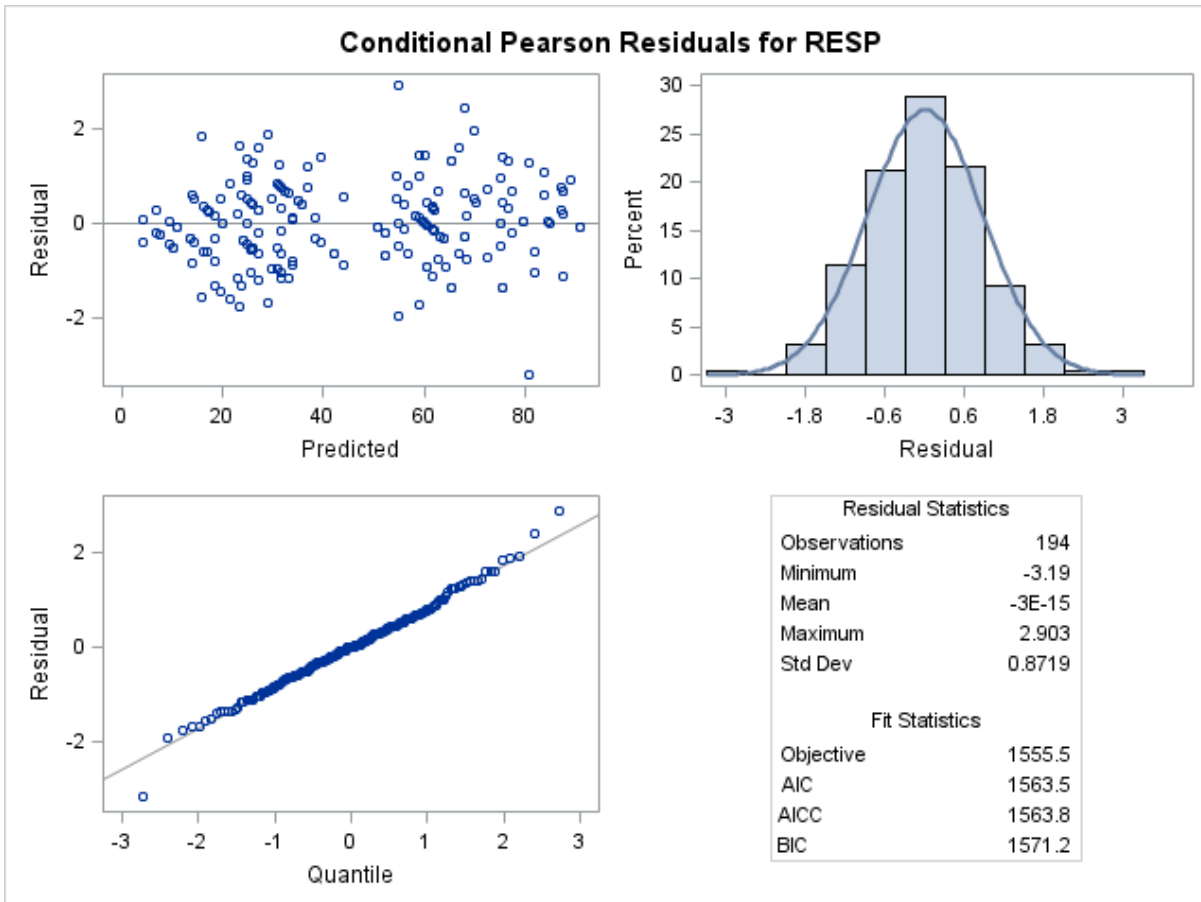

Figures S10: Diagnostic plots of Pearson residuals for the mixed model of audiometer measures

FVC FEV1

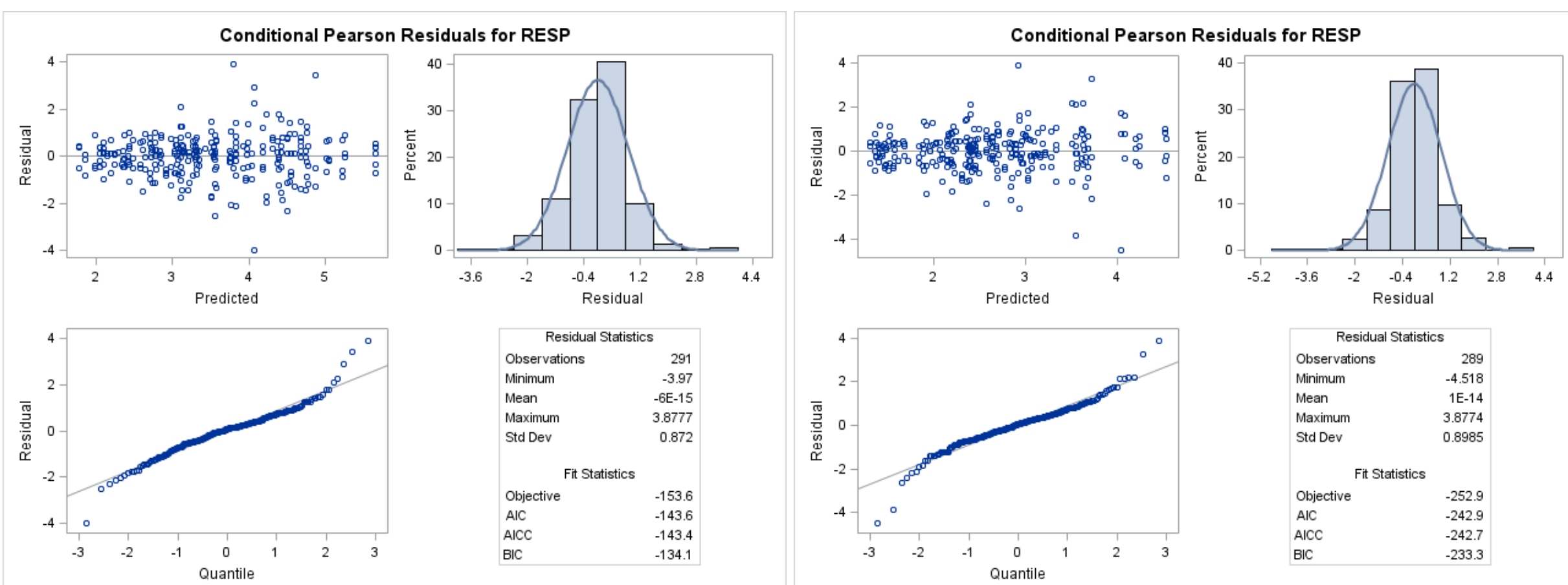

Figures S11: Diagnostic plots of Pearson residuals for the mixed model of spirometer measures

cIMT (right)

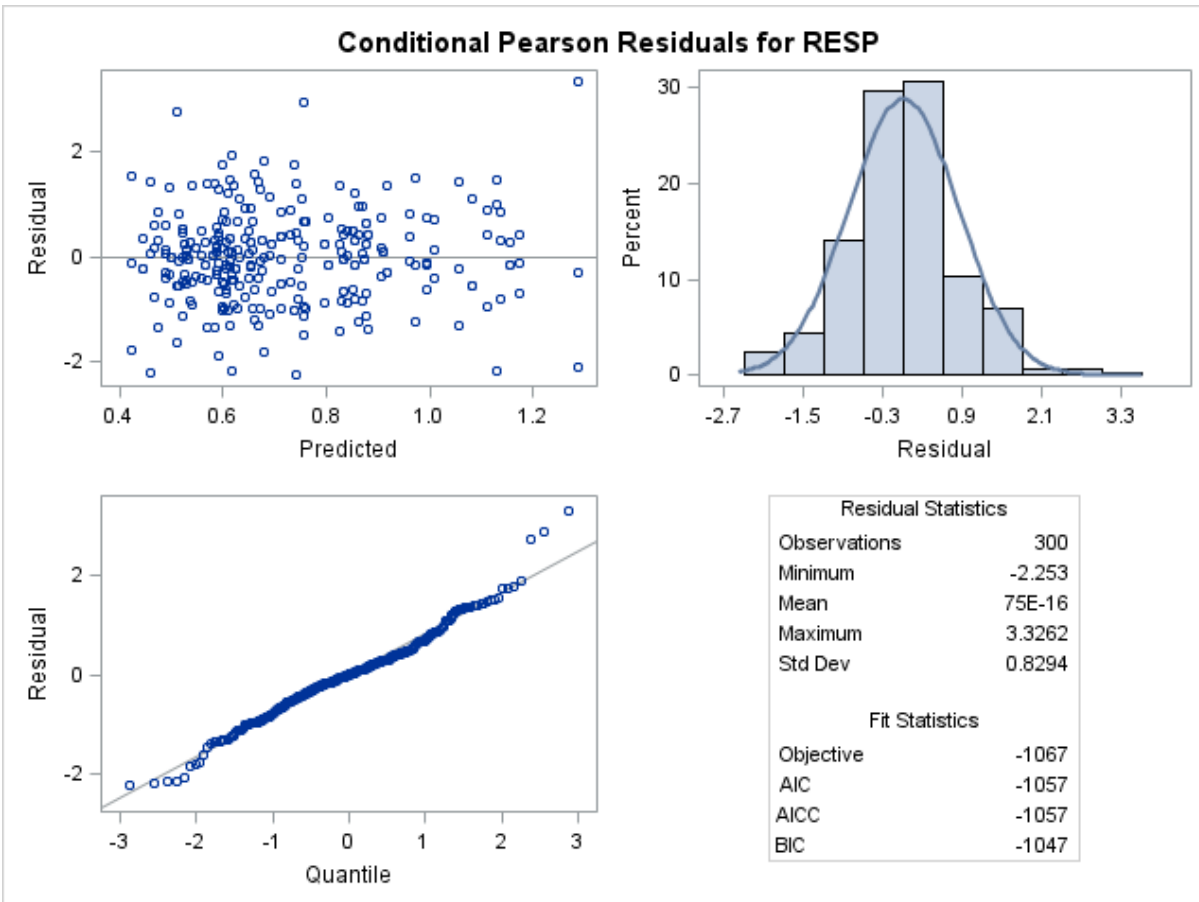

cIMT (left)

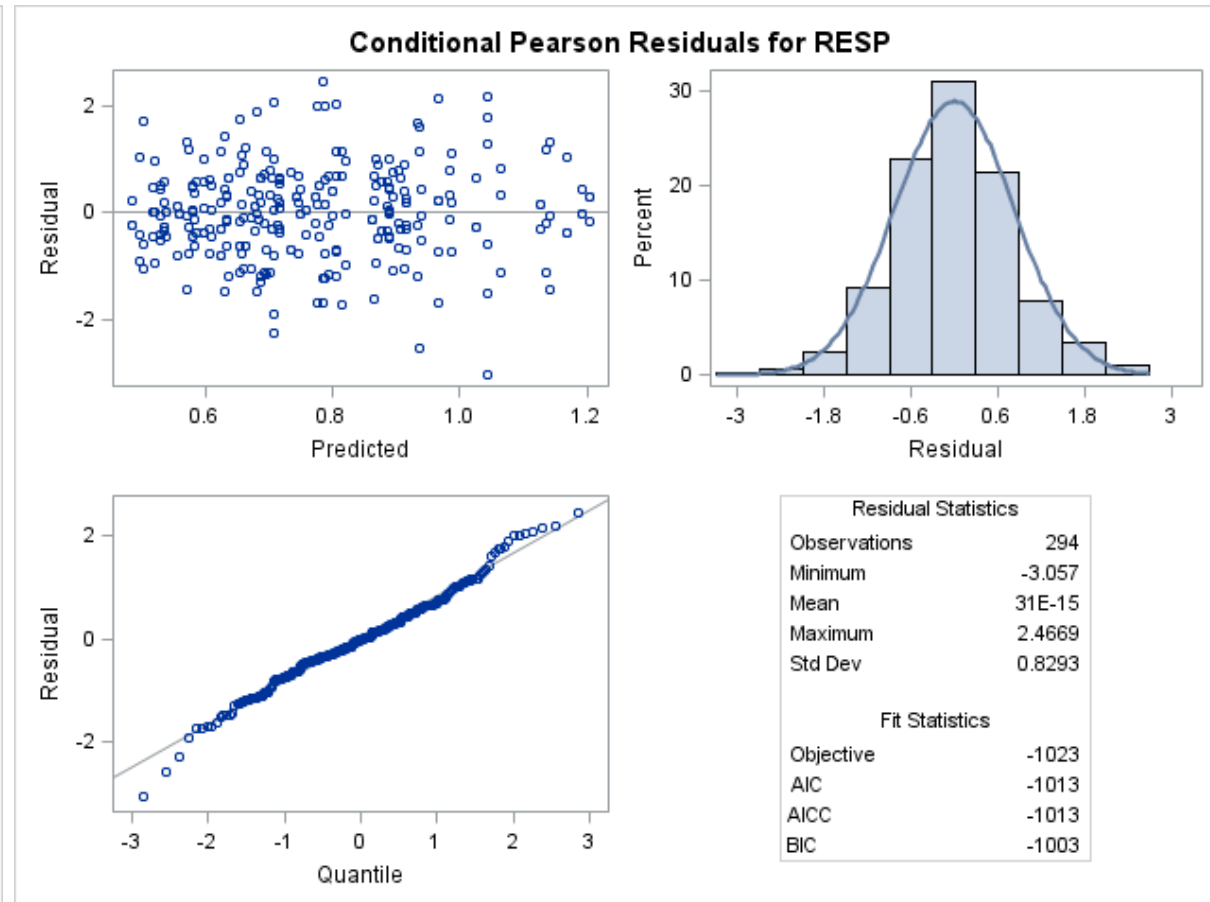

Figures S12: Diagnostic plots of Pearson residuals for the mixed model of cIMT measures

Corneal-Compensated IOP Right-Eye

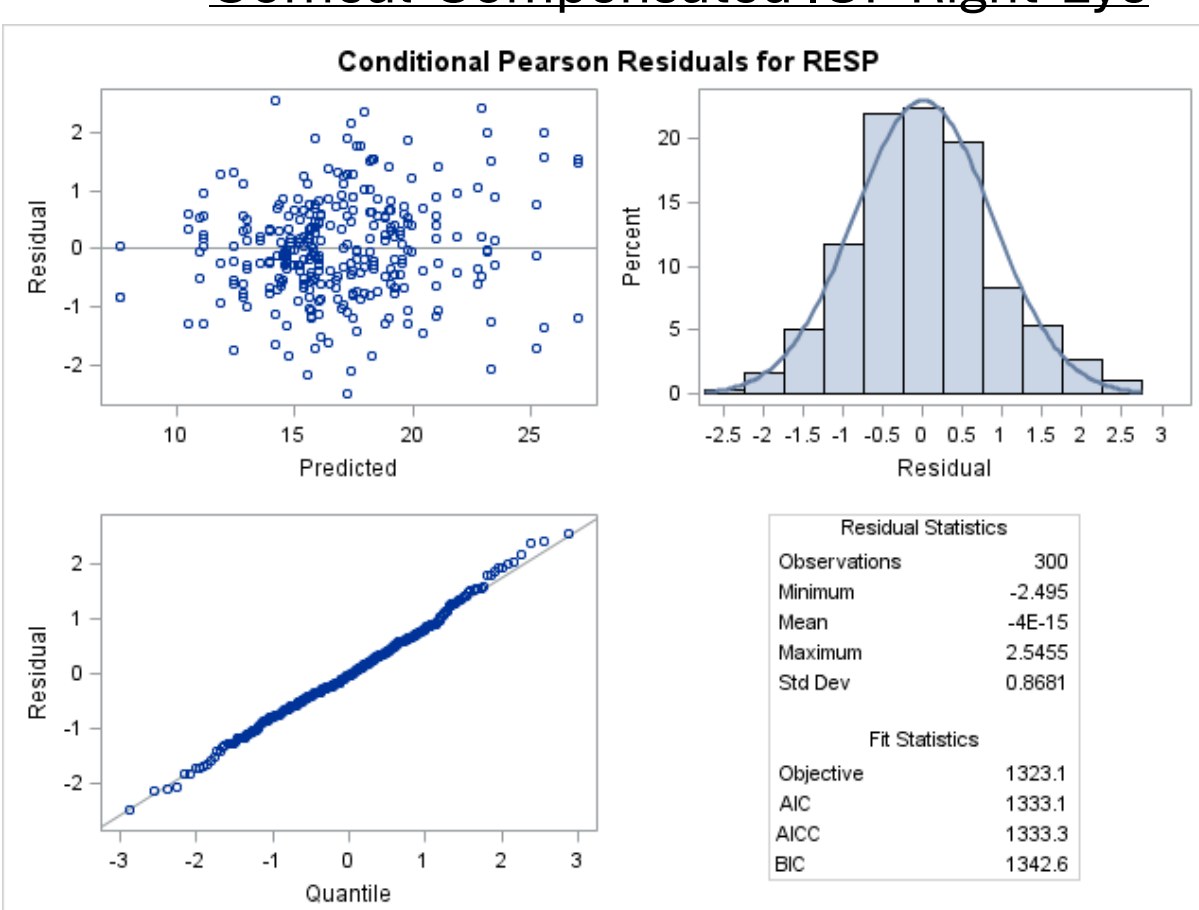

Corneal-Compensated IOP Left-Eye

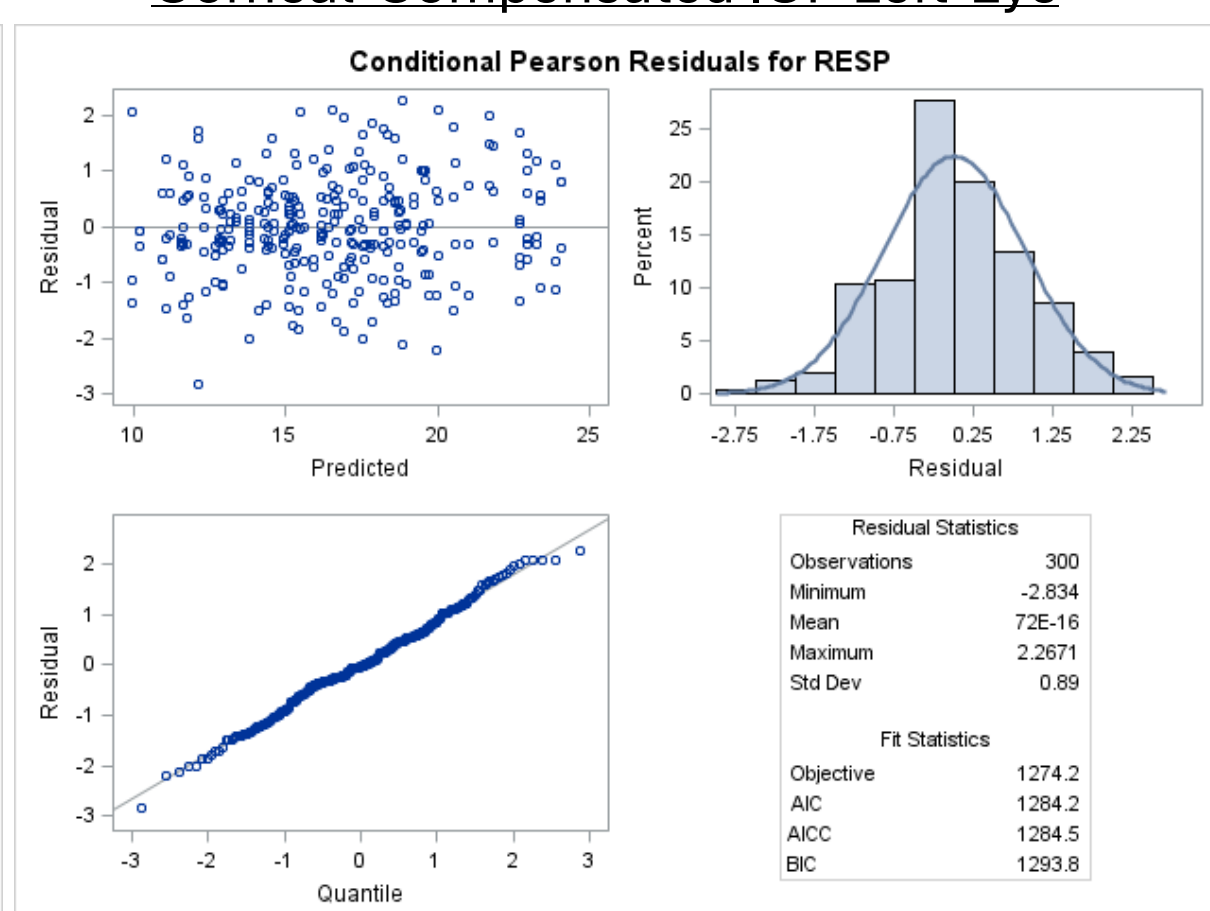

Intraocular Pressure Right-Eye

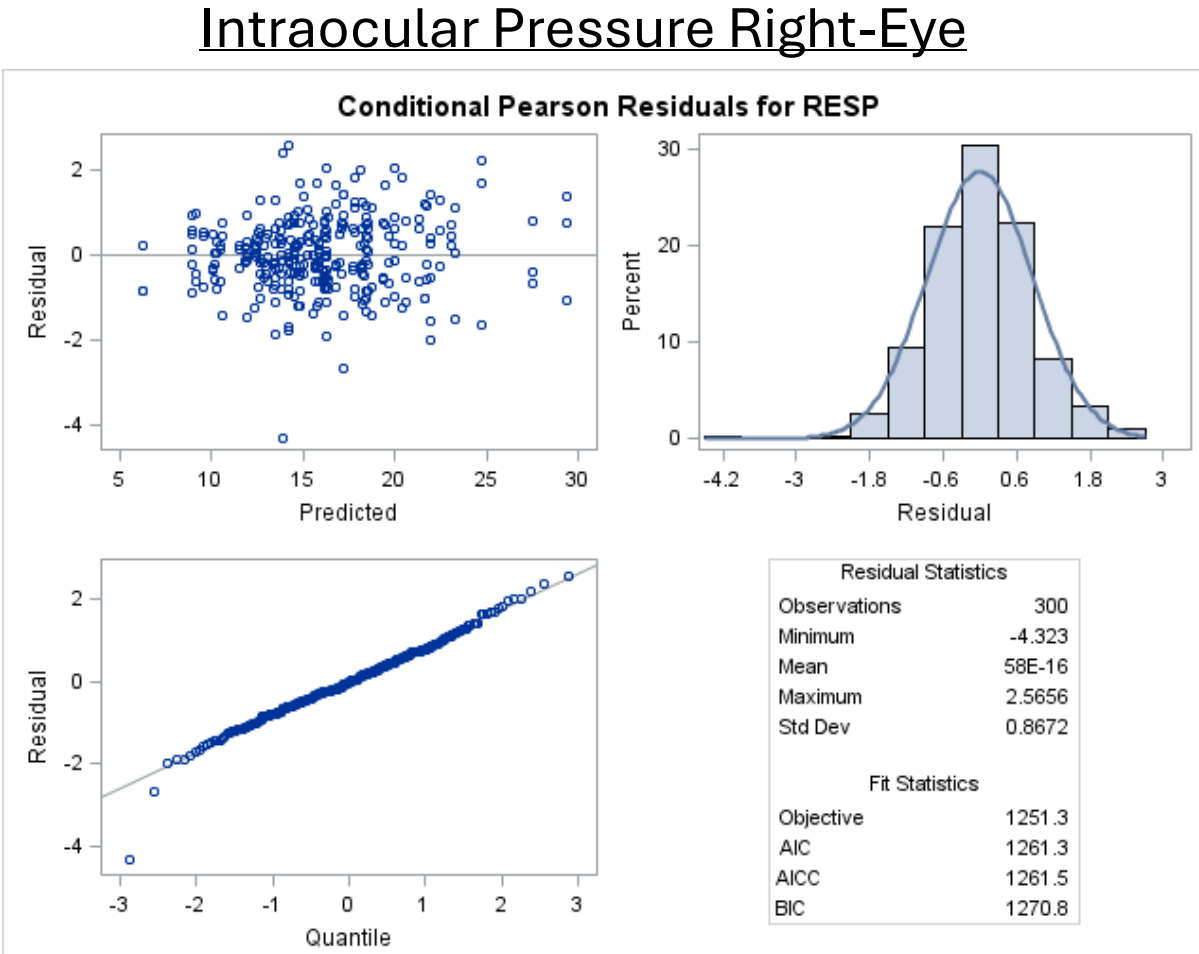

Intraocular Pressure Left-Eye

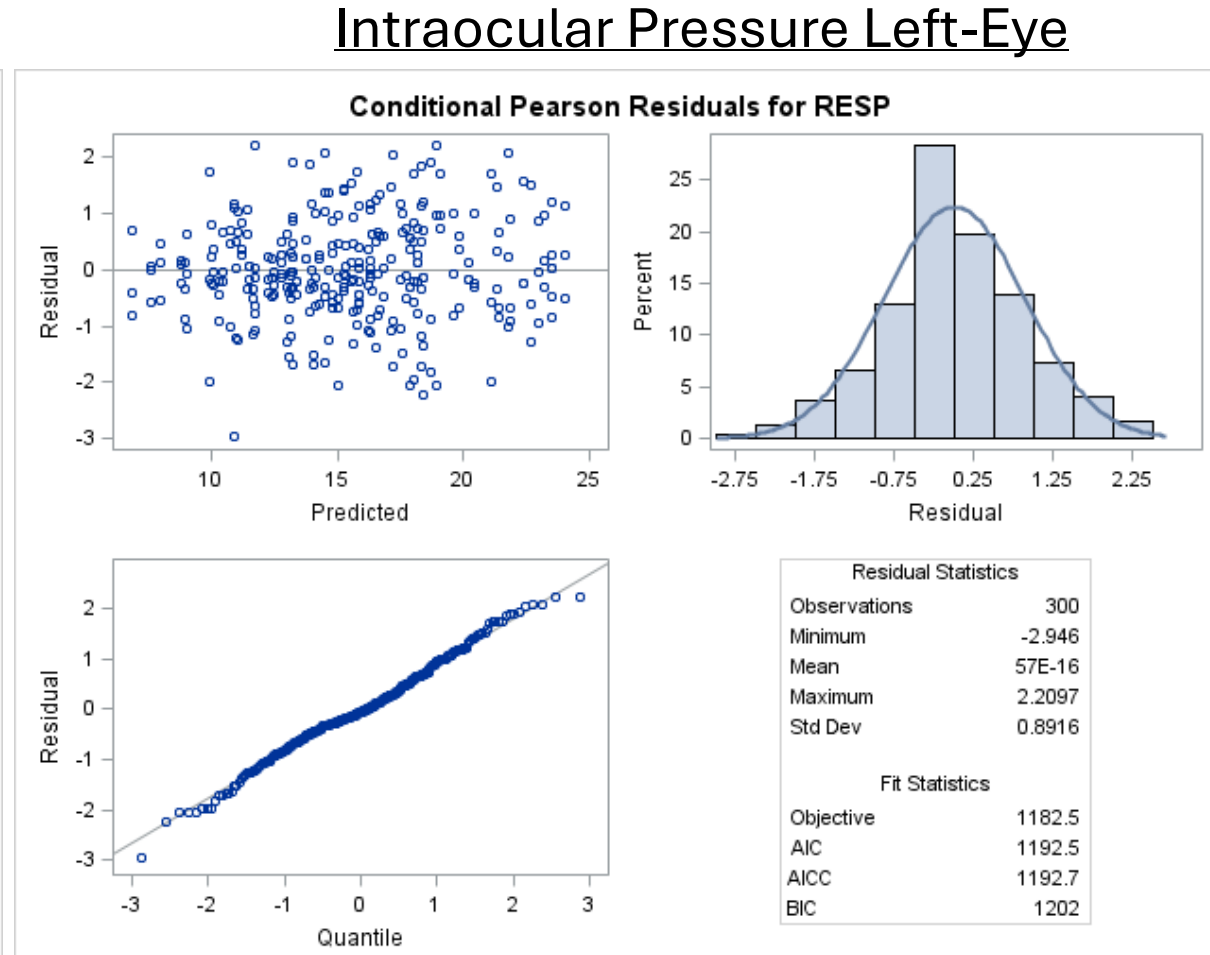

Corneal Hysteresis Right-Eye

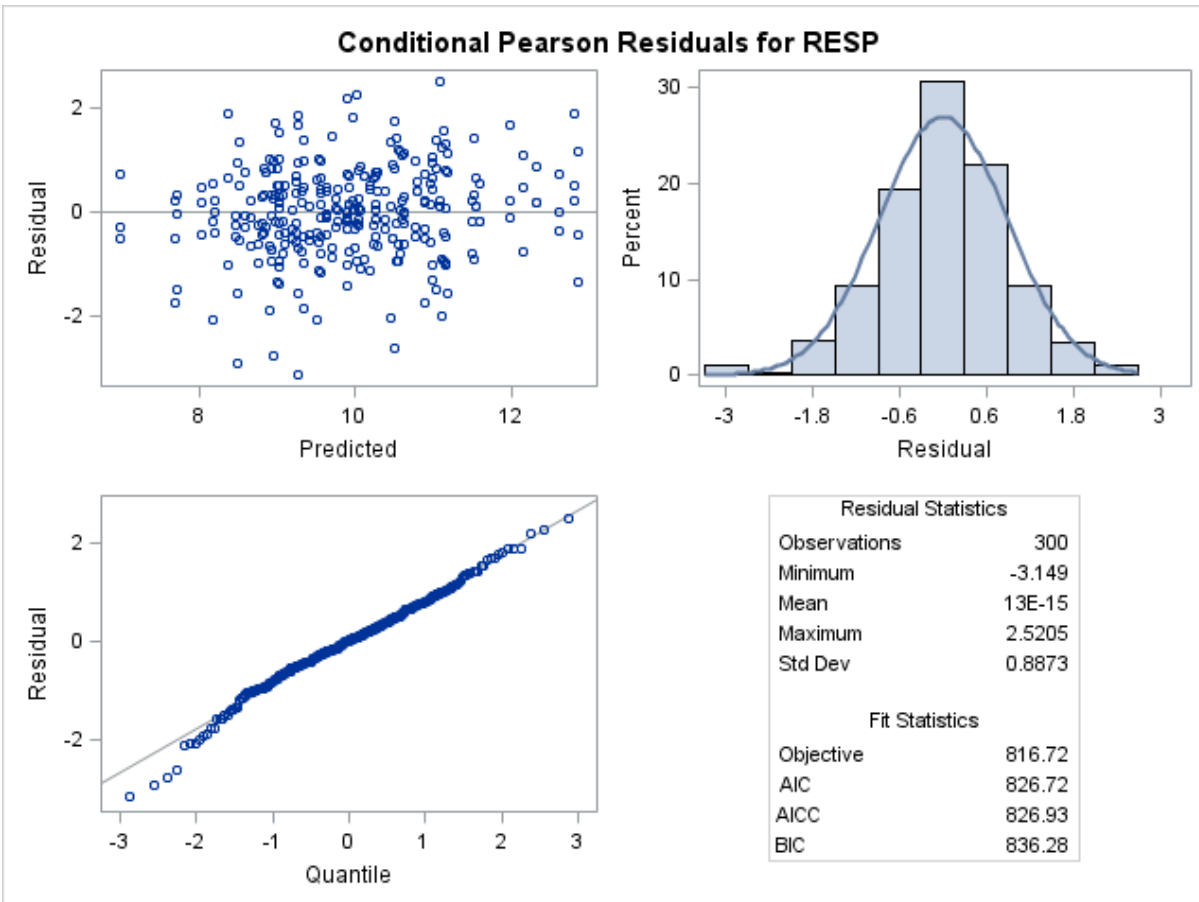

Corneal Hysteresis Left-Eye

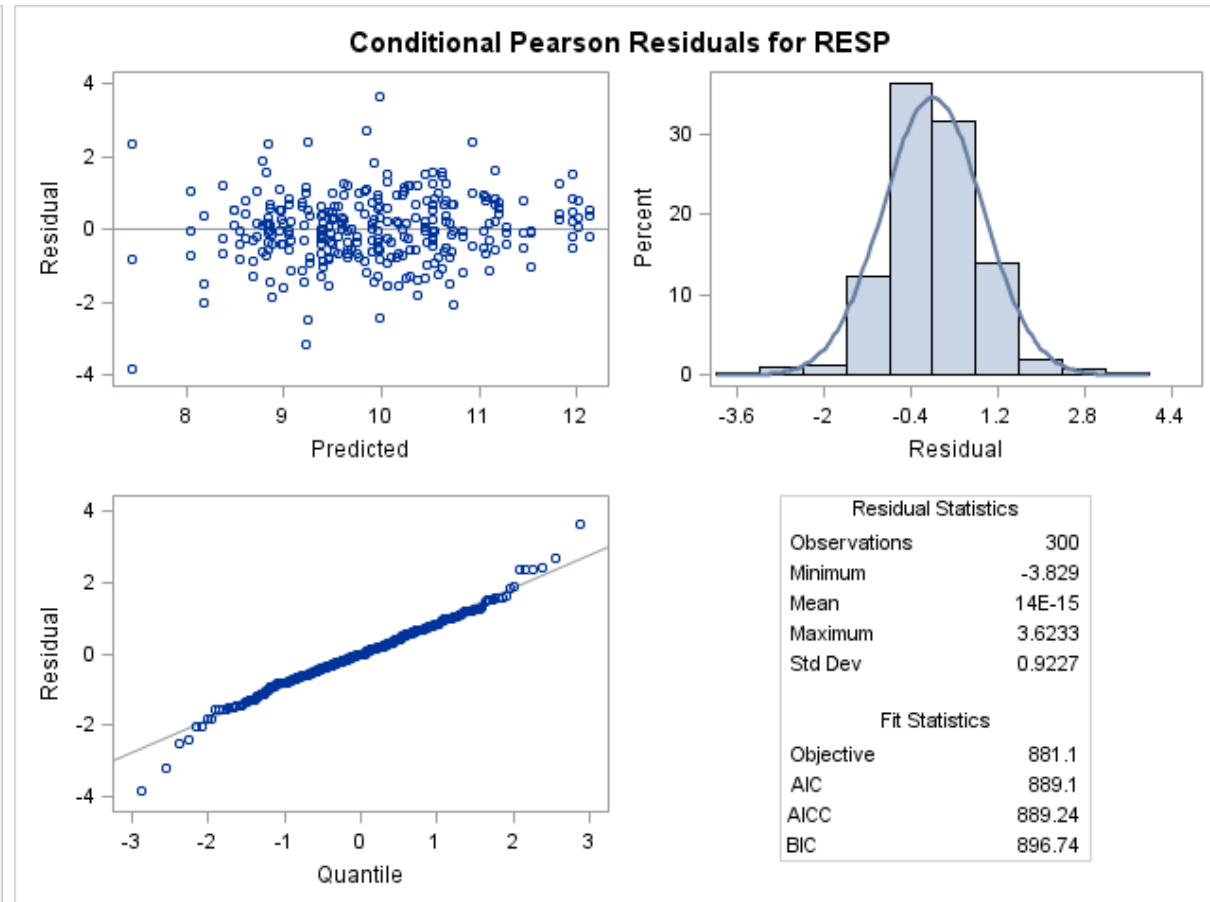

Figures S13: Diagnostic plots of Pearson residuals for the mixed model of tonometer measures

Ventricular Rate

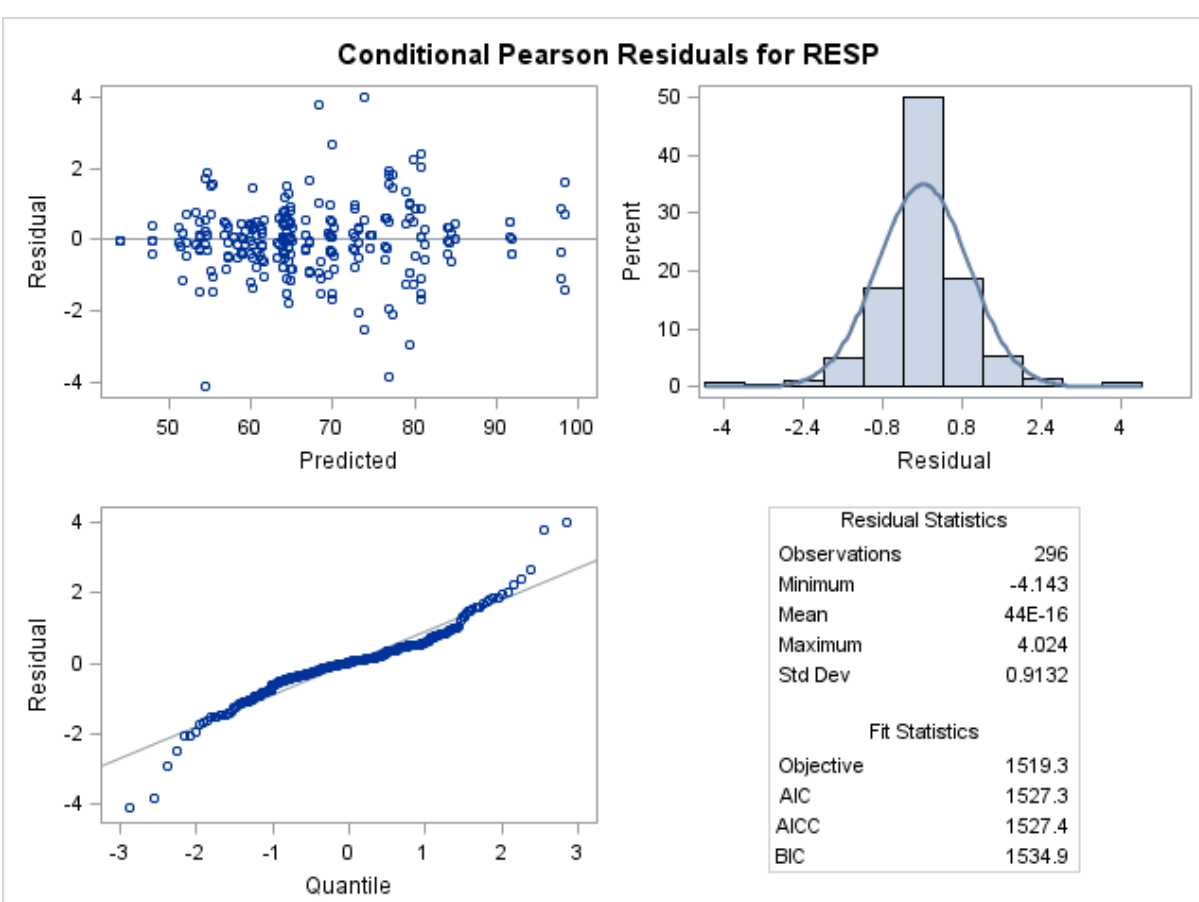

QRS Duration

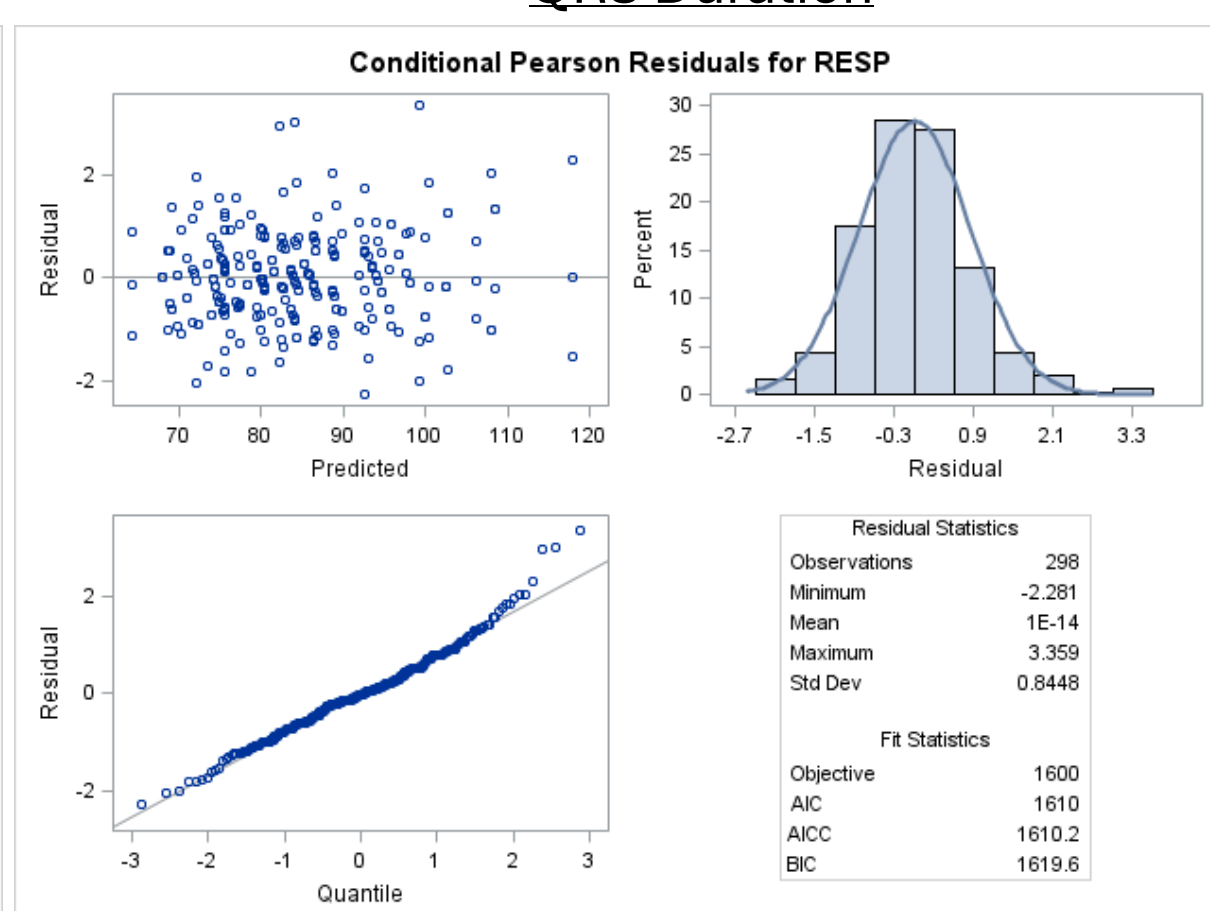

QT Interval

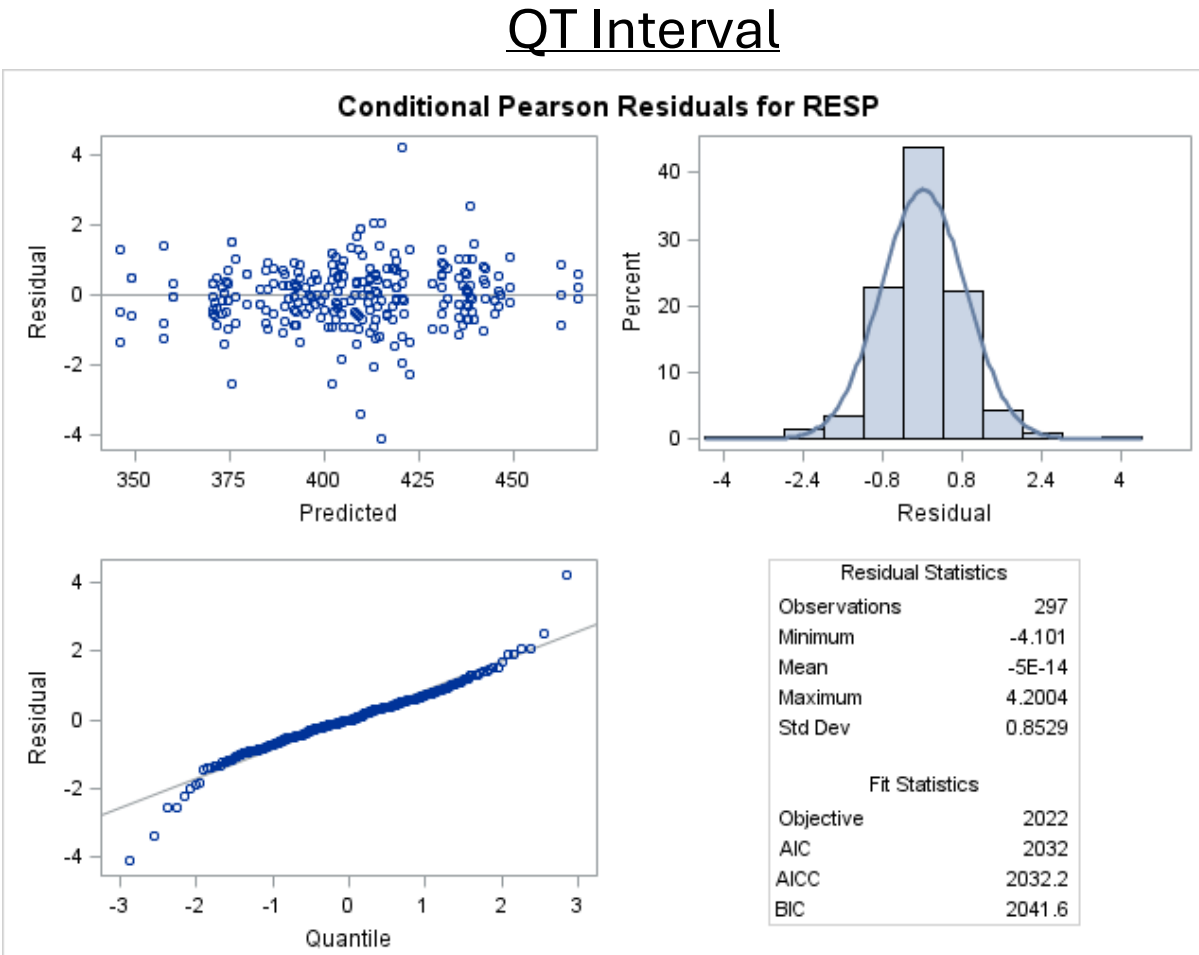

QTC Interval

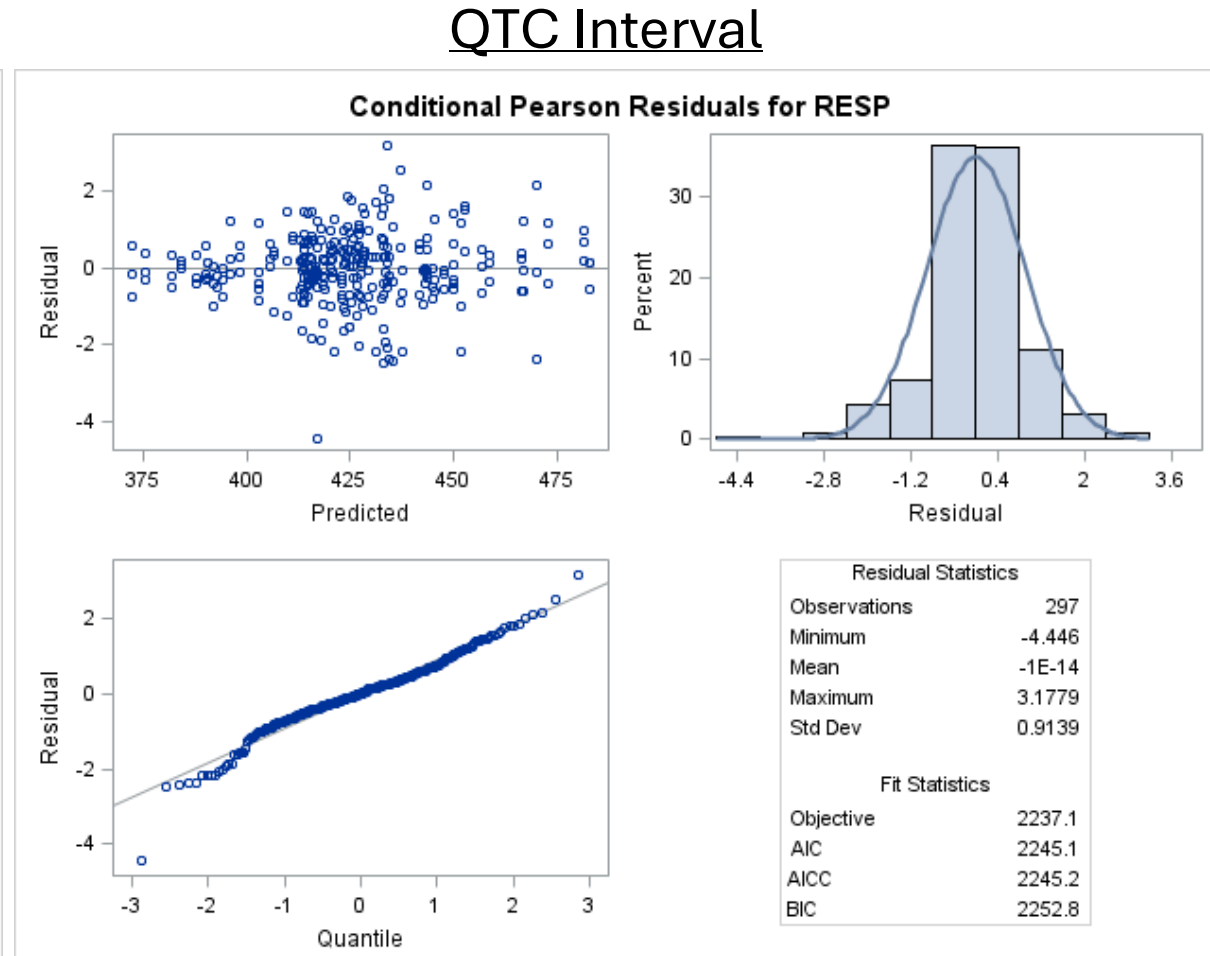

P Axis

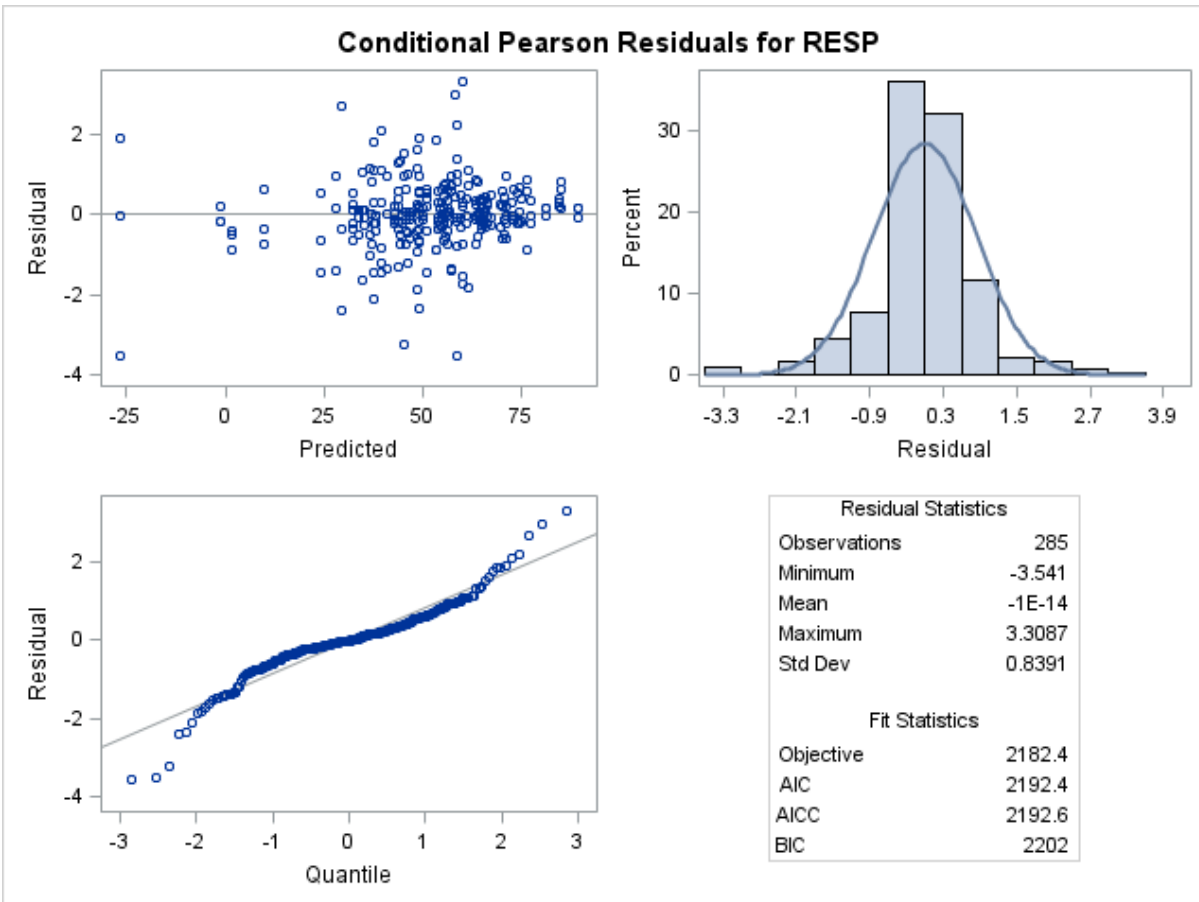

R Axis

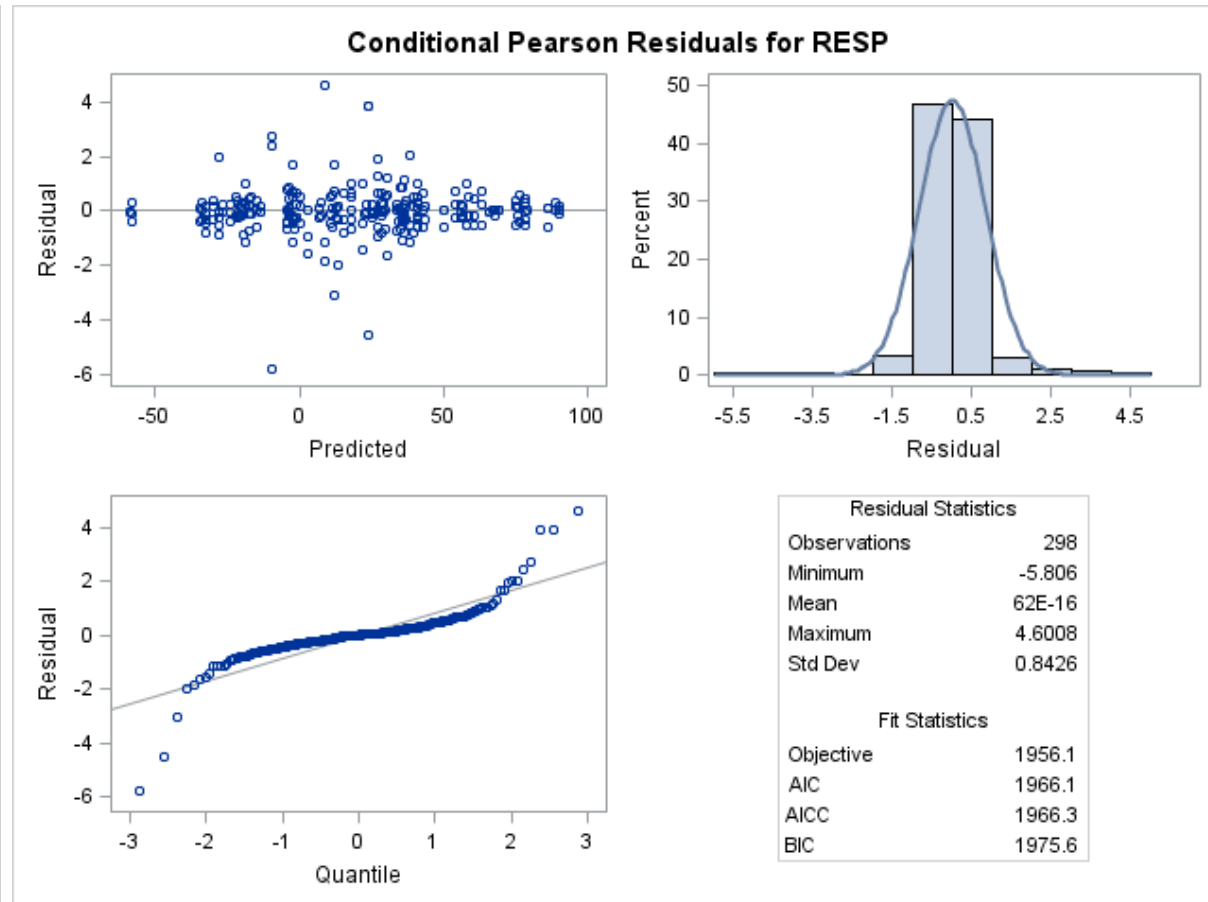

T Axis

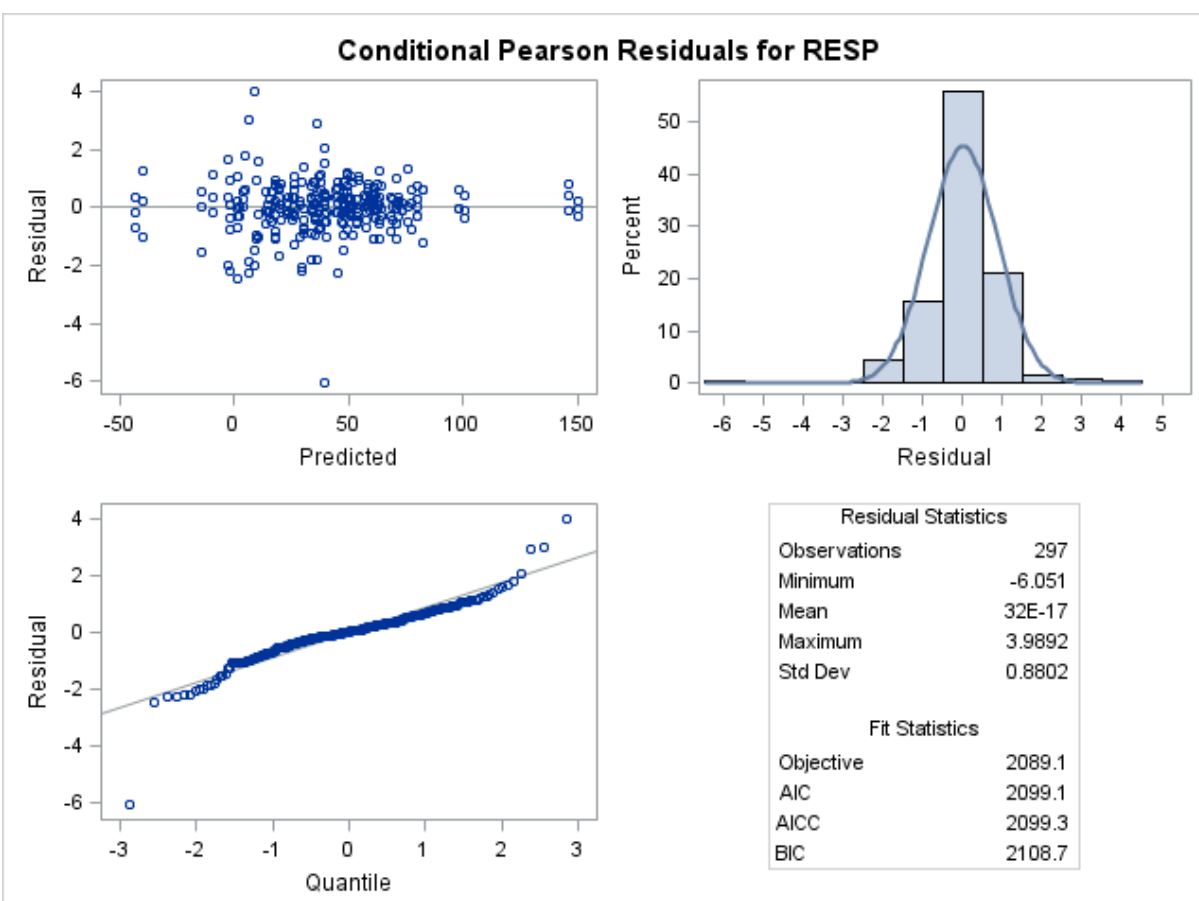

P offset

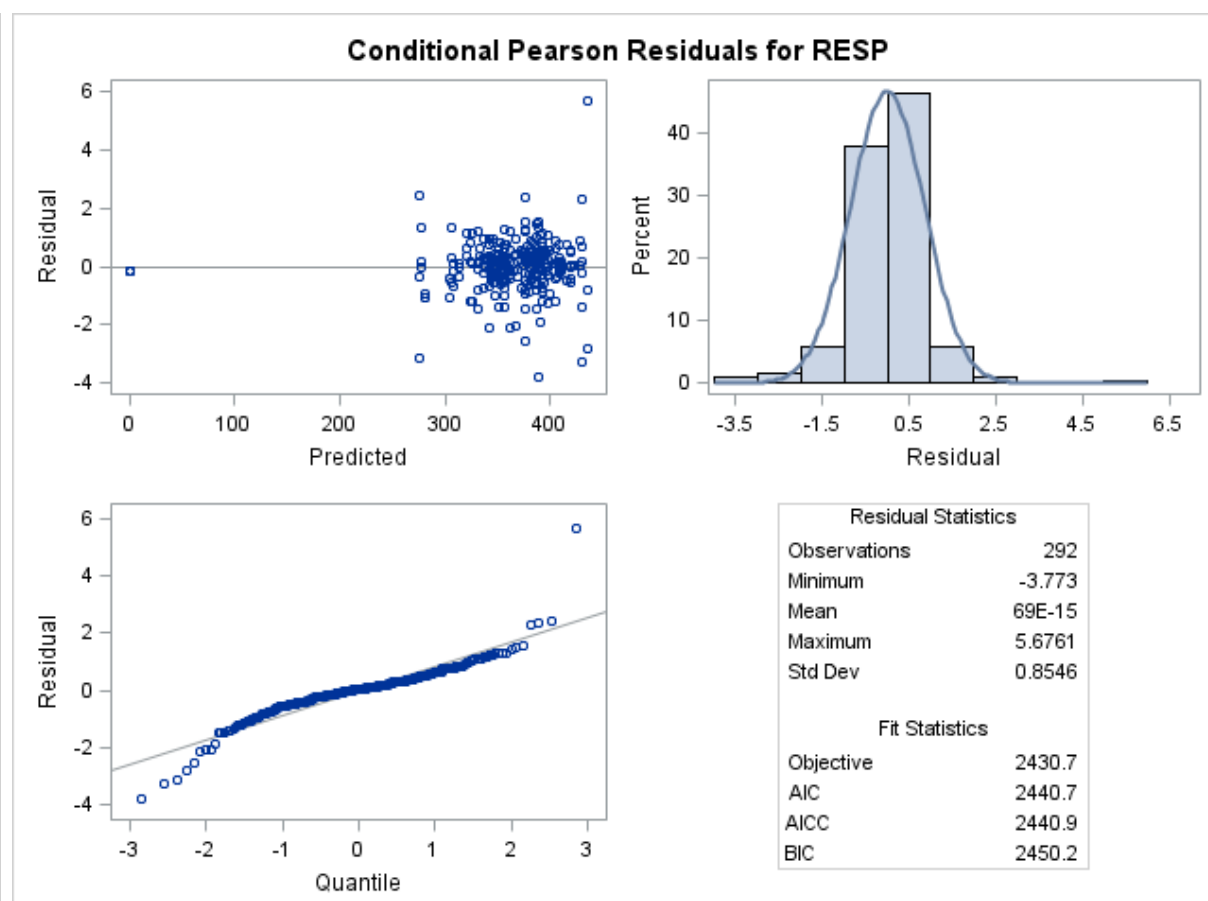

P onset

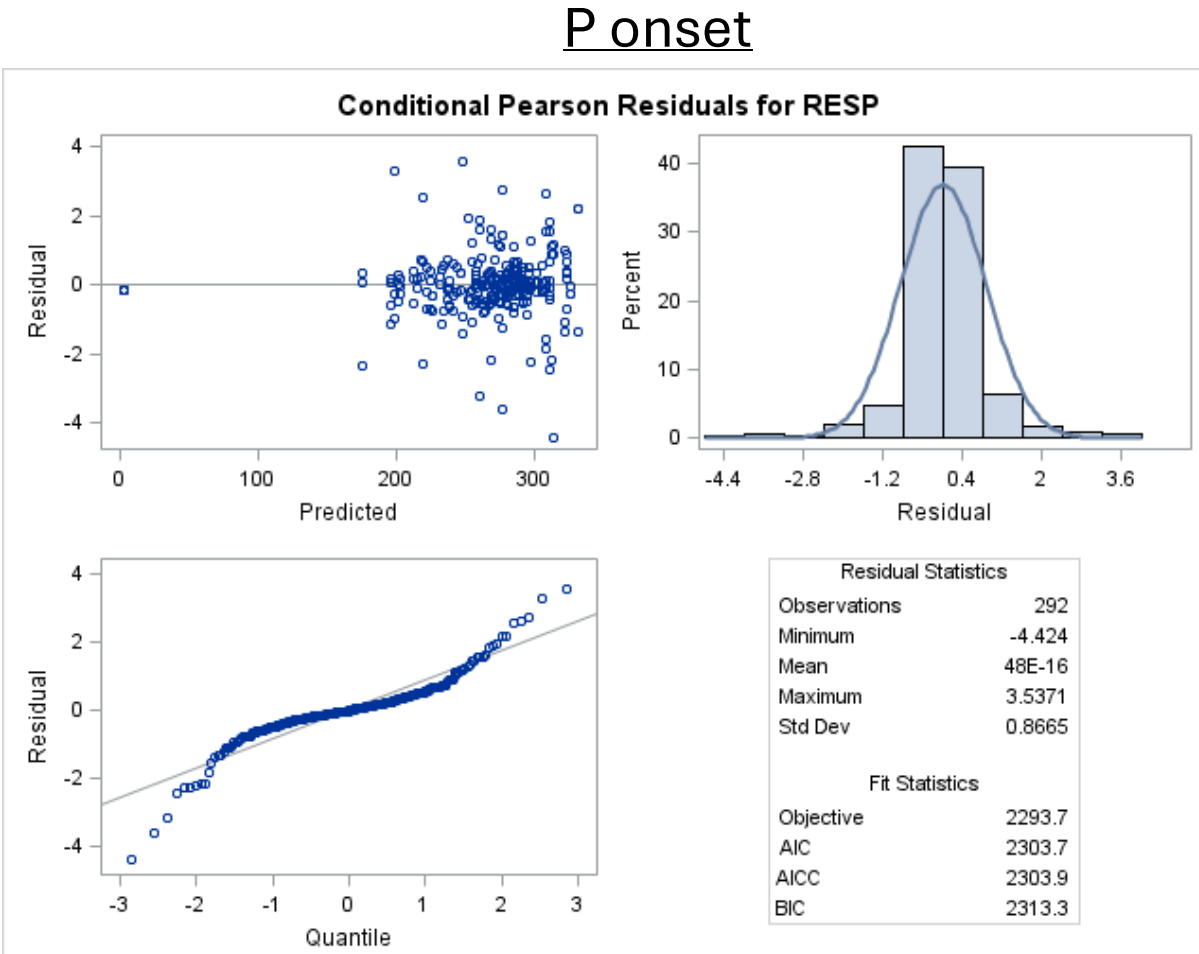

Q offset

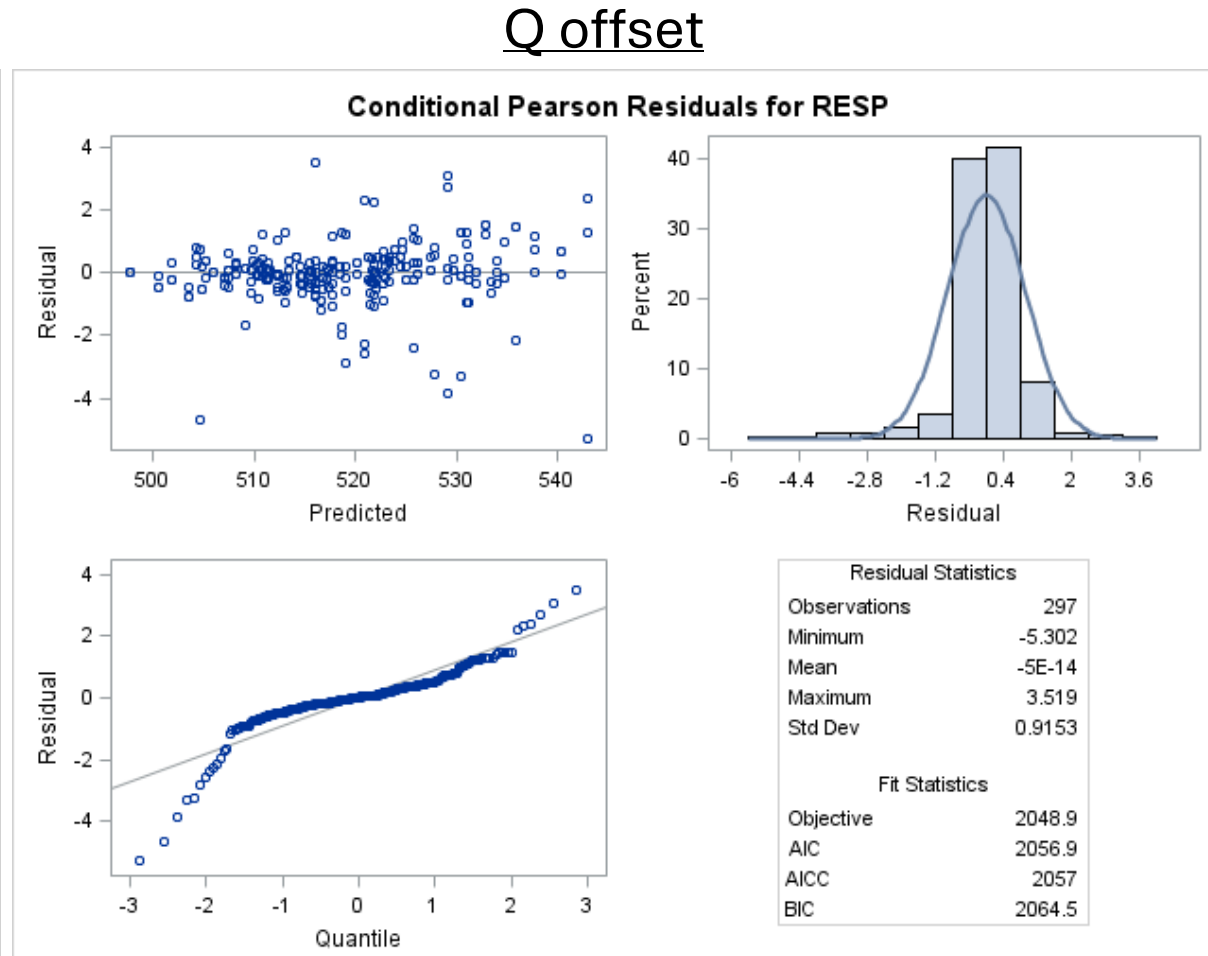

Q onset

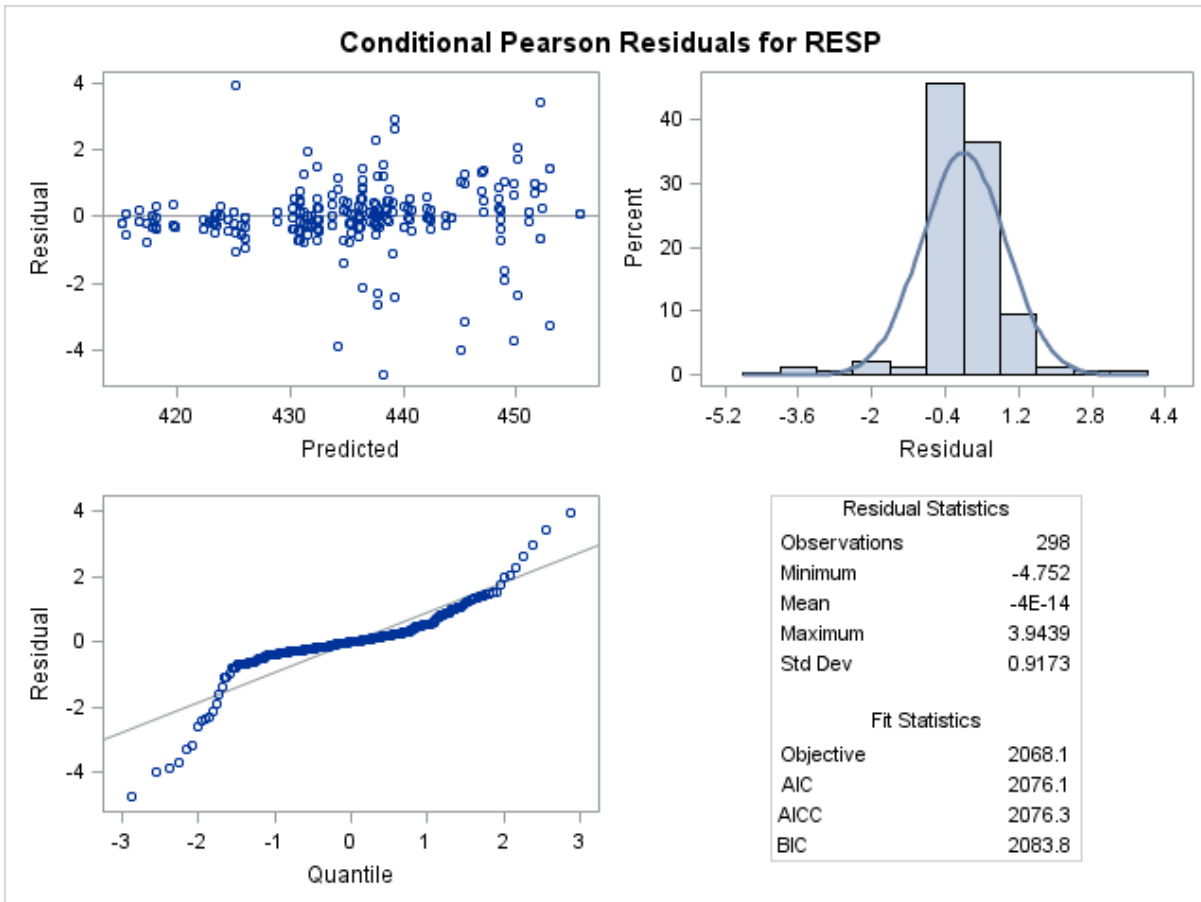

T offset

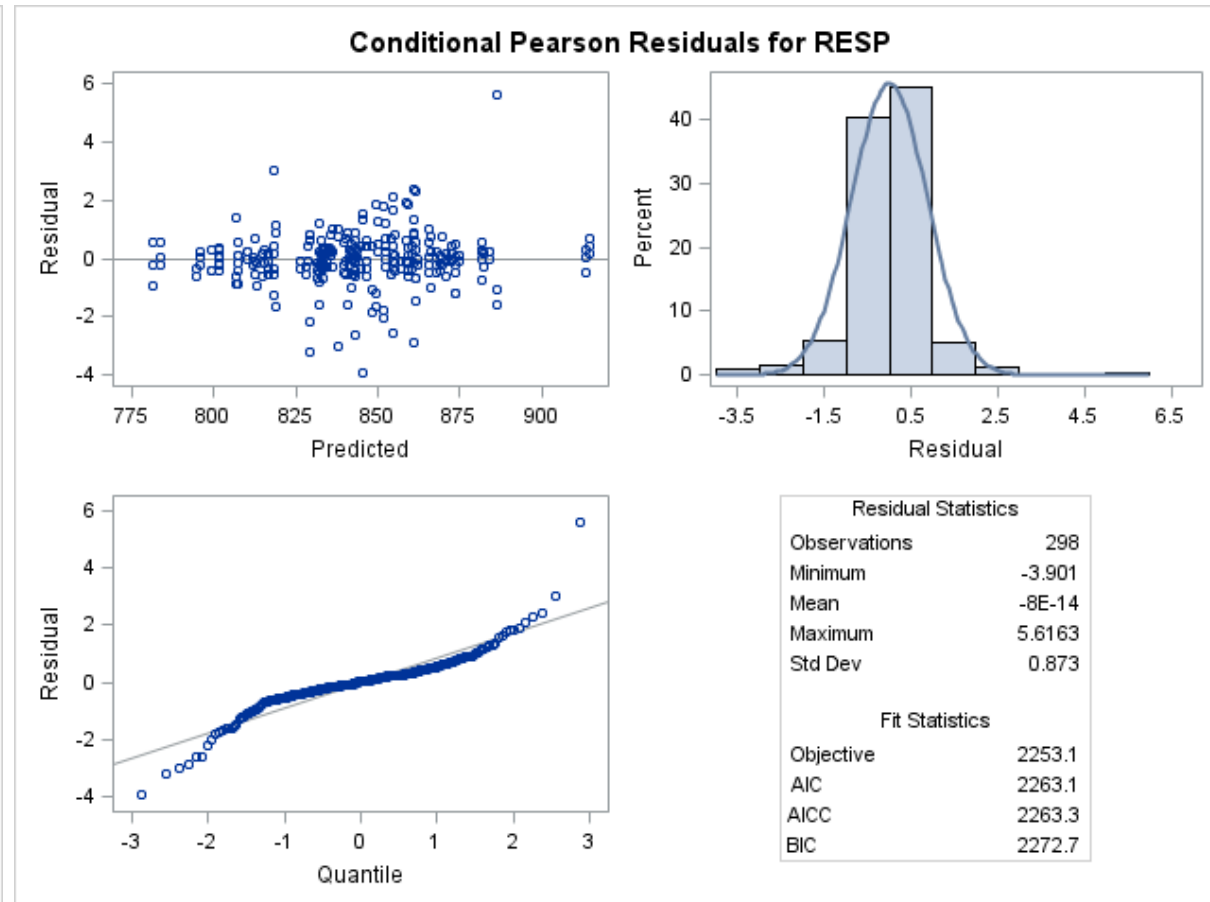

Figures S14: Diagnostic plots of Pearson residuals for the mixed model of ECG measures

Table S9A: Effect Size (Cohen's d) of the difference in SBP, DBP, and Pulse Rate measures using the old instrument between Calibration study and CLSA

	SBP	DBP	Pulse Rate
Mean (SD)<br>Calibration<br><br>CLSA	 124.99<br>(17.56)<br>121.08<br>(16.69)	 78.90<br>(11.67)<br>73.84<br>(9.99)	 70.20<br>(13.31)<br>71.55<br>(11.62)
**Cohen's *d* (95% CI)**	0.23<br>(-0.04, 0.51)	0.51<br>(0.23, 0.79)	0.12<br>(-0.16, 0.39)

Cohen's *d* effect size = .20 small, .50 medium, and .80 large.

Table S9B: Effect Size (Cohen's d) of the difference in SBP, DBP, and Pulse Rate measures using the old instrument between Calibration study and CLSA by age

	**45-54**	**55-64**	**65-74**	**75+**
**SBP**				
Mean (SD)<br>Calibration<br>CLSA	 123.60 (13.75)<br>115.22 (14.35)	 122.50 (19.82)<br>119.90 (15.88)	 127.37 (13.60)<br>124.37 (16.74)	 126.26 (22.75)<br>127.14 (18.06)
**Cohen's *d* (95% CI)**	0.58<br>(-0.04, 1.20)	0.16<br>(-0.40, 0.73)	0.18<br>(-0.36, 0.72)	0.05<br>(-0.46, 0.56)
**DBP**				
Mean (SD)<br>Calibration<br>CLSA	 83.30 (9.18)<br>75.45 (9.89)	 79.09 (12.21)<br>75.34 (9.80)	 81.13 (9.25)<br>73.07 (9.56)	 72.40 (13.82)<br>69.79 (9.81)
**Cohen's *d* (95% CI)**	0.79<br>(0.17, 1.41)	0.38<br>(-0.18, 0.95)	0.84<br>(0.30, 1.39)	0.27<br>(-0.24, 0.77)
**Pulse Rate**				
Mean (SD)<br>Calibration<br>CLSA	 74.66 (10.99)<br>72.66 (11.35)	 68.88 (14.94)<br>72.29 (11.65)	 72.54 (12.79)<br>70.94 (11.62)	 65.31 (13.43)<br>69.43 (11.59)
**Cohen's *d* (95% CI)**	0.18<br>(-0.44, 0.80)	0.29<br>(-0.27, 0.86)	0.14<br>(-0.41, 0.68)	0.36<br>(-0.15, 0.86)

Cohen's *d* effect size = .20 small, .50 medium, and .80 large.

Table S10A: Effect Size (Cohen's d) of the difference in ECG parameters using the old instrument between Calibration study and CLSA

	Ventricular Rate	QRS Duration	QT Interval	QTC Interval	P Axis	R Axis
Mean (SD)<br>Calibration<br><br>CLSA	 66.39<br>(11.31)<br>66.69<br>(11.07)	 81.37<br>(9.34)<br>86.73<br>(16.15)	 408.02<br>(25.39)<br>403.80<br>(29.87)	 425.30<br>(22.18)<br>421.60<br>(24.87)	 52.92<br>(17.82)<br>50.70<br>(22.66)	 20.58<br>(36.61)<br>26.70<br>(39.27)
**Cohen's *d***	0.03<br>(-0.25, 0.30)	0.33<br>(0.05, 0.61)	0.14<br>(-0.14, 0.42)	0.15<br>(-0.13, 0.43)	0.10<br>(-0.18, 0.38)	0.16<br>(-0.12, 0.43)
	**T Axis**	**P offset**	**P onset**	**Q offset**	**Q onset**	**T offset**
Mean (SD)<br>Calibration<br><br>CLSA	 40.37<br>(31.02)<br>42.94<br>(29.77)	 364.05<br>(36.42)<br>368.7<br>(31.89)	 272.65<br>(32.03)<br>278.9<br>(29.98)	 515.25<br>(9.19)<br>524.0<br>(14.89)	 434.00<br>(9.98)<br>437.3<br>(12.05)	 842.05<br>(26.01)<br>841.1<br>(30.02)
**Cohen's *d***	0.09<br>(-0.19, 0.36)	0.15<br>(-0.13, 0.42)	0.21<br>(-0.07, 0.49)	0.59<br>(0.31, 0.87)	0.27<br>(-0.00, 0.55)	0.03<br>(-0.25, 0.31)

Cohen's *d* effect size = .20 small, .50 medium, and .80 large.

Table S10B: Effect Size (Cohen's d) of the difference in ECG parameters using the old instrument between Calibration study and CLSA by age

	45-54	55-64	65-74	75+
**Ventricular Rate**				
Mean (SD)<br>Calibration<br>CLSA	 72.52 (11.25)<br>66.49 (10.82)	 61.92 (7.36)<br>67.0 (11.06)	 67.74 (12.58)<br>66.66 (11.03)	 64.72 (11.81)<br>66.44 (11.50)
**Cohen's *d*<br>(95% CI)**	0.56<br>(-0.06, 1.18)	0.46<br>(-0.11, 1.03)	0.10<br>(-0.45, 0.64)	0.15<br>(-0.36, 0.66)
**QRS Duration**				
Mean (SD)<br>Calibration<br>CLSA	 79.13 (11.50)<br>84.81 (11.99)	 78.72 (8.58)<br>85.59 (13.75)	 82.82 (8.05)<br>87.15 (16.82)	 83.73 (9.46)<br>91.07 (22.53)
**Cohen's *d*<br>(95% CI)**	0.47<br>(-0.15, 1.09)	0.50<br>(-0.07, 1.07)	0.26<br>(-0.29, 0.80)	0.33<br>(-0.18, 0.83)
**QT Interval**				
Mean (SD)<br>Calibration<br>CLSA	 390.43 (21.91)<br>400.43 (28.15)	 421.94 (23.64)<br>401.71 (28.69)	 409.49 (23.59)<br>405.33 (29.62)	 407.33 (25.33)<br>410.41 (33.38)
**Cohen's *d*<br>(95% CI)**	0.36<br>(-0.26, 0.98)	0.71<br>(0.14, 1.27)	0.14<br>(-0.40, 0.68)	0.09<br>(-0.41, 0.60)
**QTC Interval**				
Mean (SD)<br>Calibration<br>CLSA	 428.27 (24.64)<br>417.51 (22.47)	 426.14 (22.20)<br>420.42 (23.63)	 430.77 (23.59)<br>423.14 (24.90)	 417.92 (19.34)<br>427.48 (28.81)
**Cohen's *d***	0.48	0.24	0.31	0.33

**(95% CI)**	(-0.14, 1.10)	(-0.32, 0.81)	(-0.24, 0.85)	(-0.17, 0.84)
**P Axis**				
Mean (SD)<br>Calibration<br>CLSA	 41.97 (13.68)<br>49.22 (21.29)	 53.94 (11.73)<br>50.17 (21.72)	 54.74 (15.17)<br>51.17 (23.26)	 58.58 (24.67)<br>53.46 (25.34)
**Cohen's *d*<br>(95% CI)**	0.34<br>(-0.28, 0.96)	0.17<br>(-0.39, 0.74)	0.15<br>(-0.39, 0.70)	0.20<br>(-0.30, 0.71)
**R Axis**				
Mean (SD)<br>Calibration<br>CLSA	 29.32 (37.62)<br>38.18 (34.06)	 22.25 (40.85)<br>29.61 (36.75)	 15.67 (34.29)<br>20.66 (38.96)	 17.67 (33.48)<br>13.06 (45.21)
**Cohen's *d*<br>(95% CI)**	0.26<br>(-0.36, 0.88)	0.20<br>(-0.37, 0.77)	0.13<br>(-0.42, 0.67)	0.10<br>(-0.40, 0.61)
**T Axis**				
Mean (SD)<br>Calibration<br>CLSA	 35.33 (26.30)<br>39.95 (23.83)	 33.17 (29.75)<br>42.65 (28.01)	 39.85 (23.30)<br>43.37 (31.52)	 49.96 (40.02)<br>47.20 (36.83)
**Cohen's *d*<br>(95% CI)**	0.19<br>(-0.43, 0.81)	0.34<br>(-0.23, 0.90)	0.11<br>(-0.43, 0.66)	0.07<br>(-0.43, 0.58)
**P offset**				
Mean (SD)<br>Calibration<br>CLSA	 377.73 (21.40)<br>375.09 (25.53)	 369.61 (33.06)<br>372.47 (28.39)	 366.15 (33.92)<br>366.88 (32.11)	 346.28 (46.53)<br>353.87 (40.92)
**Cohen's *d*<br>(95% CI)**	0.10<br>(-0.52, 0.72)	0.10<br>(-0.47, 0.67)	0.02<br>(-0.52, 0.57)	0.19<br>(-0.32, 0.69)
**P onset**				
Mean (SD)<br>Calibration<br>CLSA	 289.03 (17.28)<br>285.98 (25.10)	 280.89 (30.02)<br>281.87 (27.08)	 270.87 (33.39)<br>276.42 (29.93)	 254.21 (34.51)<br>265.71 (37.12)
**Cohen's *d*<br>(95% CI)**	0.12<br>(-0.50, 0.74)	0.04<br>(-0.53, 0.60)	0.19<br>(-0.36, 0.73)	0.31<br>(-0.20, 0.82)
**Q offset**				
Mean (SD)<br>Calibration<br>CLSA	 514.47 (13.22)<br>522.46 (12.07)	 513.72 (7.49)<br>523.26 (13.60)	 515.03 (10.06)<br>524.47 (15.62)	 517.20 (6.79)<br>527.12 (18.84)
**Cohen's *d*<br>(95% CI)**	0.66<br>(0.04, 1.28)	0.70<br>(0.14, 1.27)	0.60<br>(0.06, 1.15)	0.53<br>(0.02, 1.03)
**Q onset**				
Mean (SD)<br>Calibration<br>CLSA	 436.67 (11.41)<br>437.64 (10.09)	 435.00 (10.10)<br>437.66 (11.22)	 432.21 (9.66)<br>437.31 (12.43)	 432.98 (9.75)<br>436.05 (15.15)
**Cohen's *d*<br>(95% CI)**	0.10<br>(-0.52, 0.72)	0.24<br>(-0.33, 0.80)	0.41<br>(-0.13, 0.95)	0.20<br>(-0.30, 0.71)
**T offset**				
Mean (SD)<br>Calibration<br>CLSA	 827.10 (26.68)<br>838.07 (29.02)	 856.94 (26.20)<br>839.38 (29.19)	 841.69 (24.92)<br>842.64 (29.76)	 840.42 (22.03)<br>846.46 (32.43)
**Cohen's *d*<br>(95% CI)**	0.38<br>(-0.24, 1.00)	0.60<br>(0.04, 1.17)	0.03<br>(-0.51, 0.58)	0.19<br>(-0.32, 0.69)

Cohen's *d* effect size = .20 small, .50 medium, and .80 large.

Table S11: Effect Size (Cohen's d) of the difference in cIMT using the old instrument between Calibration study and CLSA overall and by age

**cIMT right**	Overall	45-54	55-64	65-74	75+
Mean (SD)<br>Calibration<br>CLSA	 0.73 (0.20)<br>0.72 (0.20)	 0.57 (0.07)<br>0.65 (0.18)	 0.64 (0.12)<br>0.71 (0.19)	 0.75 (0.24)<br>0.76 (0.20)	 0.88 (0.14)<br>0.81 (0.21)
Cohen's *d*	0.05	0.44	0.37	0.05	0.33
**cIMT left**	Overall	45-54	55-64	65-74	75+
Mean (SD)<br>Calibration<br>CLSA	 0.78 (0.18)<br>0.74 (0.20)	 0.65 (0.12)<br>0.67 (0.18)	 0.73 (0.12)<br>0.73 (0.19)	 0.72 (0.14)<br>0.77 (0.21)	 0.94 (0.18)<br>0.82 (0.21)
Cohen's *d*	0.20	0.11	0.00	0.24	0.57

Cohen's *d* effect size = .20 small, .50 medium, and .80 large.

Table S12: Effect Size (Cohen's d) of the difference in FEV1 and FVC using the old instrument between Calibration study and CLSA overall and by age

	Overall	45-54	55-64	65-74	75+
**FEV1**					
Mean (SD)<br>Calibration<br>CLSA	 2.58 (0.78)<br>2.65 (0.76)	 2.85 (0.87)<br>3.06 (0.72)	 3.08 (0.71)<br>2.75 (0.70)	 2.31 (0.72)<br>2.41 (0.66)	 2.22 (0.59)<br>2.06 (0.61)
Cohen's *d*	0.09<br>(-0.19, 0.37)	0.29<br>(-0.33, 0.91)	0.47<br>(-0.09, 1.04)	0.15<br>(-0.41, 0.72)	0.26<br>(-0.24, 0.77)
**FVC**					
Mean (SD)<br>Calibration<br>CLSA	 3.43 (0.95)<br>3.47 (0.94)	 3.57 (1.05)<br>3.93 (0.90)	 4.04 (0.88)<br>3.57 (0.88)	 3.17 (0.85)<br>3.20 (0.84)	 3.07 (0.82)<br>2.81 (0.78)
Cohen's *d*	0.04<br>(-0.24, 0.32)	0.40<br>(-0.22, 1.02)	0.53<br>(-0.03, 1.10)	0.04<br>(-0.53, 0.60)	0.33<br>(-0.17, 0.84)

Cohen's *d* effect size = .20 small, .50 medium, and .80 large.

Table S13A: Effect Size (Cohen's d) of the difference in audiometer measures between Calibration study and CLSA using the old instrument

	Frequency 500 Hz	Frequency 1000 Hz	Frequency 2000 Hz	Frequency 3000 Hz	Frequency 4000 Hz	Frequency 6000 Hz	Frequency 8000 Hz
Mean (SD)							
Calibration	26.45 (13.61)	25.45 (14.21)	27.70 (19.23)	33.45 (21.16)	35.80 (21.10)	44.36 (22.75)	49.13 (23.98)
CLSA	20.75 (11.27)	15.06 (12.00)	18.48 (15.25)	24.88 (18.14)	30.36 (19.98)	41.78 (21.40)	45.23 (24.61)
**Cohen's *d* (95% CI)**	0.51 (0.23, 0.78)	0.87 (0.59, 1.14)	0.60 (0.33, 0.88)	0.47 (0.19, 0.75)	0.27 (-0.01, 0.55)	0.12 (-0.16, 0.40)	0.16 (-0.12, 0.44)

Cohen's *d* effect size = .20 small, .50 medium, and .80 large.

Table S13B: Effect Size (Cohen's d) of the difference in audiometer measures between Calibration study and CLSA using the old instrument by age

	**45-54**	**55-64**	**65-74**	**75+**
**Frequency 500 Hz**				
Mean (SD)				
Calibration	26.45 (13.61)	22.29 (10.95)	27.31 (15.36)	32.00 (16.48)
CLSA	17.34 (9.82)	19.79 (10.63)	21.95 (11.07)	27.27 (12.39)
**Cohen's *d* (95% CI)**	0.93 (0.31, 1.55)	0.32 (-0.25, 0.88)	0.48 (-0.06, 1.03)	0.38 (-0.13, 0.89)
**Frequency 1000 Hz**				
Mean (SD)				
Calibration	17.50 (5.65)	23.54 (9.50)	26.92 (18.26)	31.00 (15.69)
CLSA	10.11 (8.55)	12.80 (9.92)	16.83 (11.98)	24.11 (14.25)
**Cohen's *d* (95% CI)**	0.86 (0.24, 1.48)	1.08 (0.52, 1.65)	0.84 (0.30, 1.39)	0.48 (-0.02, 0.99)
**Frequency 2000 Hz**				
Mean (SD)				
Calibration	14.00 (7.09)	24.58 (14.41)	30.96 (22.58)	36.50 (20.63)
CLSA	10.11 (9.65)	14.84 (11.92)	21.93 (14.94)	32.79 (16.51)
**Cohen's *d* (95% CI)**	0.40 (-0.22, 1.02)	0.82 (0.25, 1.38)	0.60 (0.06, 1.15)	0.22 (-0.28, 0.73)
**Frequency 3000 Hz**				
Mean (SD)				
Calibration	15.75 (6.46)	28.33 (15.68)	37.50 (23.56)	45.83 (21.12)
CLSA	13.74 (11.80)	20.84 (14.96)	29.99 (17.31)	41.73 (17.74)
**Cohen's *d* (95% CI)**	0.17 (-0.45, 0.79)	0.50 (-0.07, 1.07)	0.43 (-0.11, 0.98)	0.23 (-0.28, 0.74)
**Frequency 4000 Hz**				
Mean (SD)				
Calibration	21.50 (10.29)	27.50 (15.70)	40.38 (23.05)	48.00 (21.36)
CLSA	17.28 (13.50)	26.12 (16.78)	36.51 (18.49)	49.01 (18.31)
**Cohen's *d* (95% CI)**	0.31 (-0.31, 0.93)	0.08 (-0.48, 0.65)	0.21 (-0.33, 0.75)	0.06 (-0.45, 0.56)
**Frequency 6000 Hz**				

Mean (SD)<br>Calibration<br>CLSA	 29.00 (15.33)<br>27.73 (15.06)	 34.96 (17.82)<br>36.95 (18.06)	 51.88 (27.46)<br>48.35 (19.50)	 56.39 (17.54)<br>62.61 (18.19)
**Cohen's *d*<br>(95% CI)**	0.08<br>(-0.54, 0.70)	0.11<br>(-0.46, 0.68)	0.18<br>(-0.36, 0.72)	0.34<br>(-0.16, 0.85)
**Frequency 8000 Hz**				
Mean (SD)<br>Calibration<br>CLSA	 32.00 (14.71)<br>26.22 (16.91)	 33.75 (20.46)<br>39.38 (20.31)	 55.77 (26.39)<br>54.81 (20.81)	 67.10 (12.20)<br>71.33 (16.82)
**Cohen's *d*<br>(95% CI)**	0.34<br>(-0.28, 0.96)	0.28<br>(-0.29, 0.84)	0.05<br>(-0.50 0.59)	0.25<br>(-0.26, 0.76)

Cohen's *d* effect size = .20 small, .50 medium, and .80 large.

Table S14A: Effect Size (Cohen's d) of the difference in tonometer measures using the old instrument between Calibration study and CLSA

	Corneal-Compensated IOP (right-eye)	Corneal-Compensate d IOP (left-eye)	Intraocular Pressure (right-eye)	Intraocular Pressure (left-eye)	Corneal Hysteresis (right-eye)	Corneal Hysteresis (left-eye)
Mean (SD)<br>Calibration<br>CLSA	 17.45 (3.28)<br>15.89 (4.05)	 16.90 (3.39)<br>16.34 (4.17)	 16.12 (3.85)<br>15.12 (3.87)	 15.63 (3.84)<br>15.28 (3.91)	 9.70 (1.27)<br>10.20 (2.02)	 9.65 (1.23)<br>9.90 (2.13)
**Cohen's *d*<br>(95% CI)**	0.39<br>(0.11, 0.66)	0.13<br>(-0.14, 0.41)	0.26<br>(-0.02, 0.54)	0.09<br>(-0.19, 0.37)	0.25<br>(-0.03, 0.53)	0.12<br>(-0.16, 0.39)

Cohen's *d* effect size = .20 small, .50 medium, and .80 large.

Table S14B: Effect Size (Cohen's d) of the difference in tonometer measures using the old instrument between Calibration study and CLSA by age

	**45-54**	**55-64**	**65-74**	**75+**
**Corneal-Compensated IOP (right eye)**				
Mean (SD)<br>Calibration<br>CLSA	 18.85 (3.87)<br>15.07 (3.65)	 16.33 (1.86)<br>15.85 (3.86)	 18.24 (3.57)<br>16.4 (4.22)	 16.74 (3.30)<br>16.49 (4.49)
**Cohen's *d*<br>(95% CI)**	1.04<br>(0.42, 1.66)	0.12<br>(-0.44, 0.69)	0.44<br>(-0.11, 0.98)	0.06<br>(-0.45, 0.56)
**Corneal-Compensated IOP (left eye)**				
Mean (SD)<br>Calibration<br>CLSA	 18.59 (3.05)<br>15.50 (3.71)	 15.87 (2.68)<br>16.27 (4.07)	 17.55 (3.25)<br>16.82 (4.20)	 16.03 (3.93)<br>17.04 (4.70)
**Cohen's *d*<br>(95% CI)**	0.83<br>(0.21, 1.45)	0.10<br>(-0.47, 0.66)	0.17<br>(-0.37, 0.72)	0.21<br>(-0.29, 0.72)

Intraocular Pressure (right eye)				
Mean (SD)<br>Calibration<br>CLSA	 18.58 (3.99)<br>14.58 (3.66)	 14.57 (2.51)<br>15.25 (3.79)	 17.06 (3.36)<br>15.54 (3.95)	 14.90 (4.26)<br>15.08 (4.13)
**Cohen's *d*<br>(95% CI)**	1.09<br>(0.47, 1.71)	0.18<br>(-0.39, 0.75)	0.38<br>(-0.16, 0.93)	0.04<br>(-0.46, 0.55)
**Intraocular Pressure (left eye)**				
Mean (SD)<br>Calibration<br>CLSA	 17.65 (3.57)<br>14.69 (3.64)	 14.75 (2.89)<br>15.39 (3.82)	 16.30 (3.53)<br>15.71 (3.99)	 14.41 (4.52)<br>15.33 (4.24)
**Cohen's *d*<br>(95% CI)**	0.81<br>(0.19, 1.43)	0.17<br>(-0.40, 0.73)	0.15<br>(-0.40, 0.69)	0.22<br>(-0.29, 0.72)
**Corneal Hysteresis (right-eye)**				
Mean (SD)<br>Calibration<br>CLSA	 10.19 (1.99)<br>10.52 (1.95)	 9.83 (1.19)<br>10.33 (1.92)	 9.64 (0.90)<br>10.06 (1.96)	 9.33 (0.97)<br>9.63 (2.26)
**Cohen's *d*<br>(95% CI)**	0.17<br>(-0.45, 0.79)	0.26<br>(-0.31, 0.83)	0.21<br>(-0.33, 0.76)	0.13<br>(-0.37, 0.64)
**Corneal Hysteresis (left eye)**				
Mean (SD)<br>Calibration<br>CLSA	 9.72 (1.46)<br>10.22 (2.03)	 9.80 (1.56)<br>10.06 (2.07)	 9.63 (0.71)<br>9.80 (2.13)	 9.48 (1.23)<br>9.29 (2.24)
**Cohen's *d*<br>(95% CI)**	0.25<br>(-0.37, 0.87)	0.13<br>(-0.44, 0.69)	0.08<br>(-0.46, 0.62)	0.08<br>(-0.42, 0.59)

Cohen's *d* effect size = .20 small, .50 medium, and .80 large.

Table S15: Effect Size (Cohen's d) of the difference in grip strength using the old instrument between Calibration study and CLSA overall and by age

	Overall	45-54	55-64	65-74	75+
Mean (SD)<br>Calibration<br>CLSA	 32.53 (11.31)<br>33.36 (11.47)	 35.77 (13.20)<br>37.39 (11.89)	 37.27 (13.79)<br>34.37 (11.26)	 31.47 (7.53)<br>31.77 (10.53)	 27.50 (9.12)<br>27.51 (9.51)
**Cohen's *d*<br>(95% CI)**	0.07<br>(-0.21, 0.35)	0.14<br>(-0.48, 0.76)	0.26<br>(-0.31, 0.82)	0.03<br>(-0.52, 0.57)	0.00<br>(-0.51, 0.51)

Cohen's *d* effect size = .20 small, .50 medium, and .80 large.

Table S16: Intraclass correlation coefficient (ICC) for key variables obtained from the old and new instruments

	ICC[a]	
	**Old Instrument**	**New Instrument**
**Blood pressure monitor**		
Systolic blood pressure (mmHg)	0.89 (0.82, 0.94)	0.88 (0.80, 0.93)
Diastolic blood pressure (mmHg)	0.90 (0.83, 0.94)	0.85 (0.76, 0.91)
Pulse rate (bpm)	0.97 (0.95, 0.98)	0.94 (0.89, 0.96)
**Spirometer (L)**		
FEV1	0.96 (0.93, 0.98)	0.97 (0.95, 0.98)
FVC	0.97 (0.95, 0.98)	0.96 (0.93, 0.98)
**Carotid ultrasound (mm)**		
cIMT right	0.98 (0.97, 0.99)	0.99 (0.97, 0.99)
cIMT left	0.98 (0.96, 0.99)	0.98 (0.96, 0.99)
**Grip strength (kg)**	0.88 (0.81, 0.93)	0.92 (0.87, 0.96)
**Audiometer (dB HL)**		
Frequency 500 Hz	0.67 (0.53, 0.78)	0.65 (0.52, 0.77)
Frequency 1000 Hz	0.71 (0.57, 0.81)	0.73 (0.60, 0.83)
Frequency 2000 Hz	0.82 (0.72, 0.89)	0.77 (0.66, 0.86)
Frequency 3000 Hz	0.74 (0.62, 0.84)	0.78 (0.66, 0.86)
Frequency 4000 Hz	0.79 (0.68, 0.87)	0.85 (0.76, 0.91)
Frequency 6000 Hz	0.68 (0.54, 0.79)	0.80 (0.69, 0.88)
Frequency 8000 Hz	0.70 (0.57, 0.81)	0.76 (0.64, 0.85)
**Tonometer (mmHg)**		
Corneal-compensated IOP - right-eye	0.76 (0.65, 0.85)	0.85 (0.76, 0.91)
Corneal-compensated IOP - left-eye	0.80 (0.69, 0.87)	0.86 (0.78, 0.92)
Intraocular pressure - right-eye	0.86 (0.78, 0.92)	0.91 (0.85, 0.95)
Intraocular pressure - left-eye	0.90 (0.84, 0.94)	0.92 (0.86, 0.95)
Corneal hysteresis - right-eye	0.58 (0.44, 0.71)	0.77 (0.65, 0.85)
Corneal hysteresis - left-eye	0.45 (0.31, 0.59)	0.57 (0.42, 0.70)
**Electrocardiogram (ECG)**		
Ventricular rate (bpm)	0.83 (0.73, 0.90)	0.87 (0.79, 0.92)
QRS duration (ms)	0.95 (0.91, 0.97)	0.93 (0.89, 0.96)
QT interval (ms)	0.92 (0.86, 0.95)	0.94 (0.89, 0.96)
QTC interval (ms)	0.74 (0.61, 0.83)	0.87 (0.80, 0.93)
P axis (degree)	0.71 (0.58, 0.81)	0.93 (0.89, 0.96)
R axis (degree)	0.99 (0.98, 0.99)	0.99 (0.99, 1.00)
T axis (degree)	0.95 (0.91, 0.97)	0.99 (0.98, 0.99)
P offset (ms)	0.97 (0.95, 0.98)	0.99 (0.98, 0.99)
P onset (ms)	0.97 (0.95, 0.98)	0.98 (0.97, 0.99)
Q offset (ms)	0.57 (0.42, 0.70)	0.78 (0.67, 0.87)
Q onset (ms)	0.78 (0.67, 0.86)	0.78 (0.66, 0.86)
T offset (ms)	0.85 (0.77, 0.91)	0.86 (0.77, 0.92)

[a]ICC quantifies the proportion of total variability attributable to differences between individuals, relative to total variability.

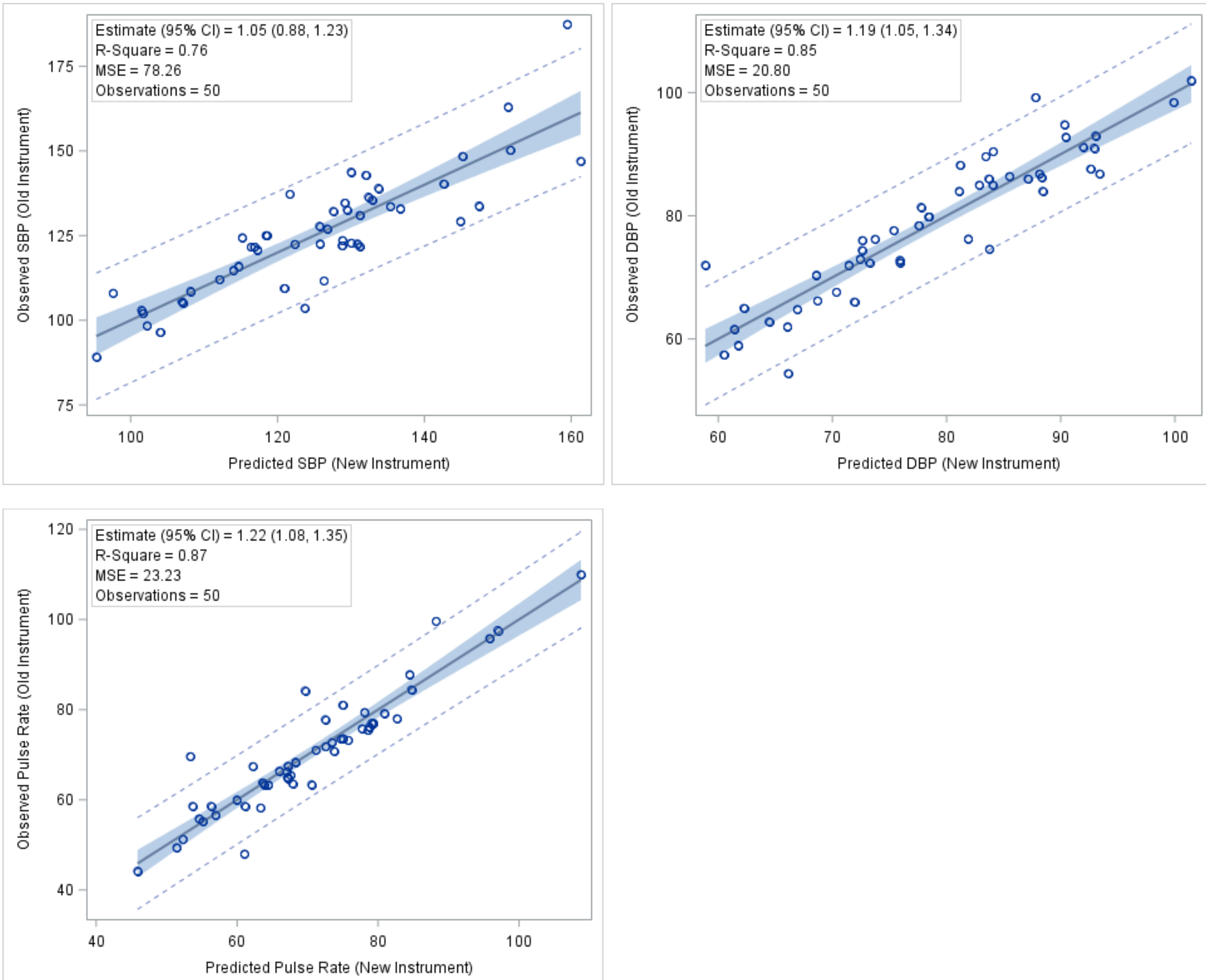

Figure S15: Fit Plots for Cross-calibration of SBP, DBP, and Pulse Rate

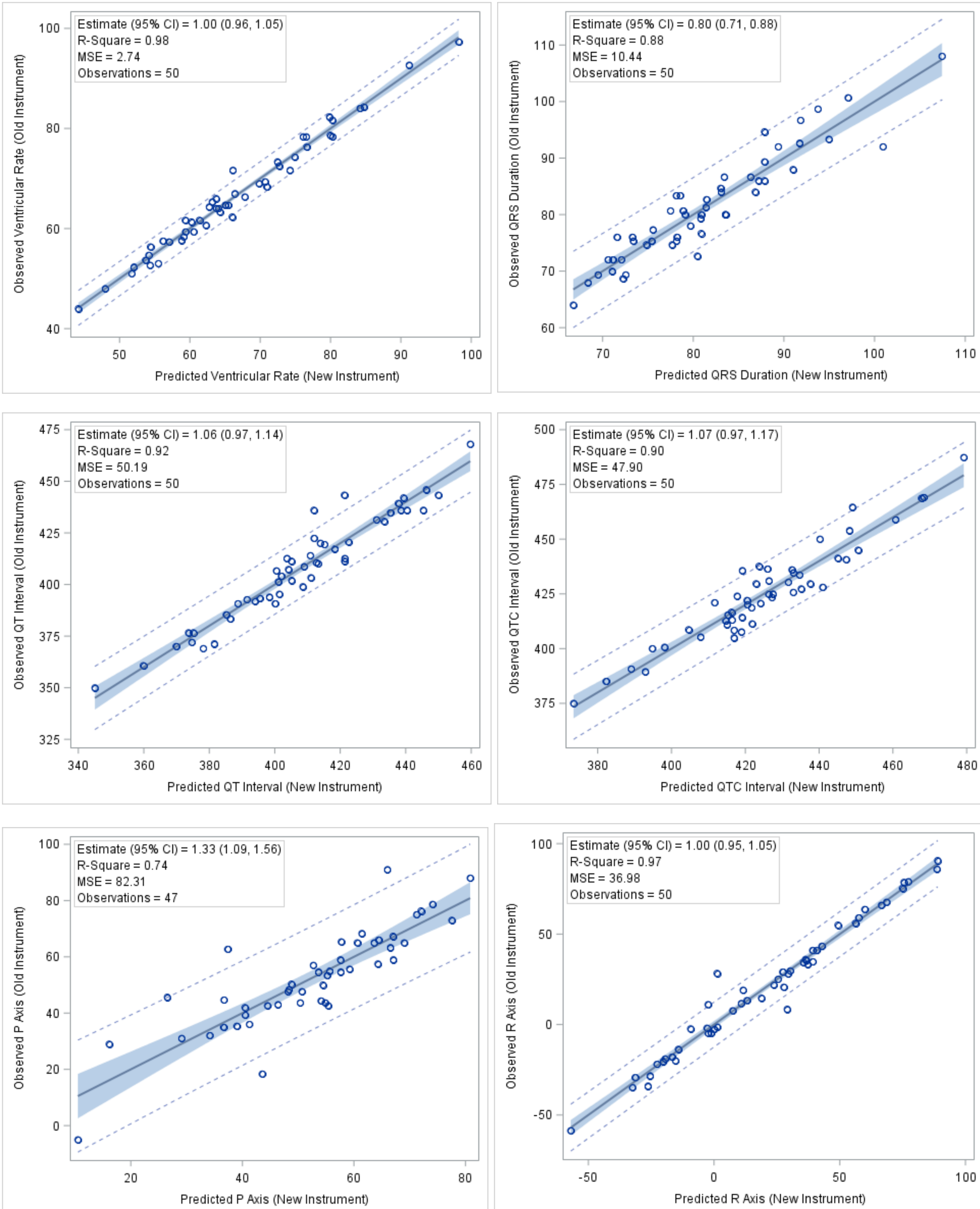

Figure S16A: Fit Plots for Cross-calibration of ECG measures

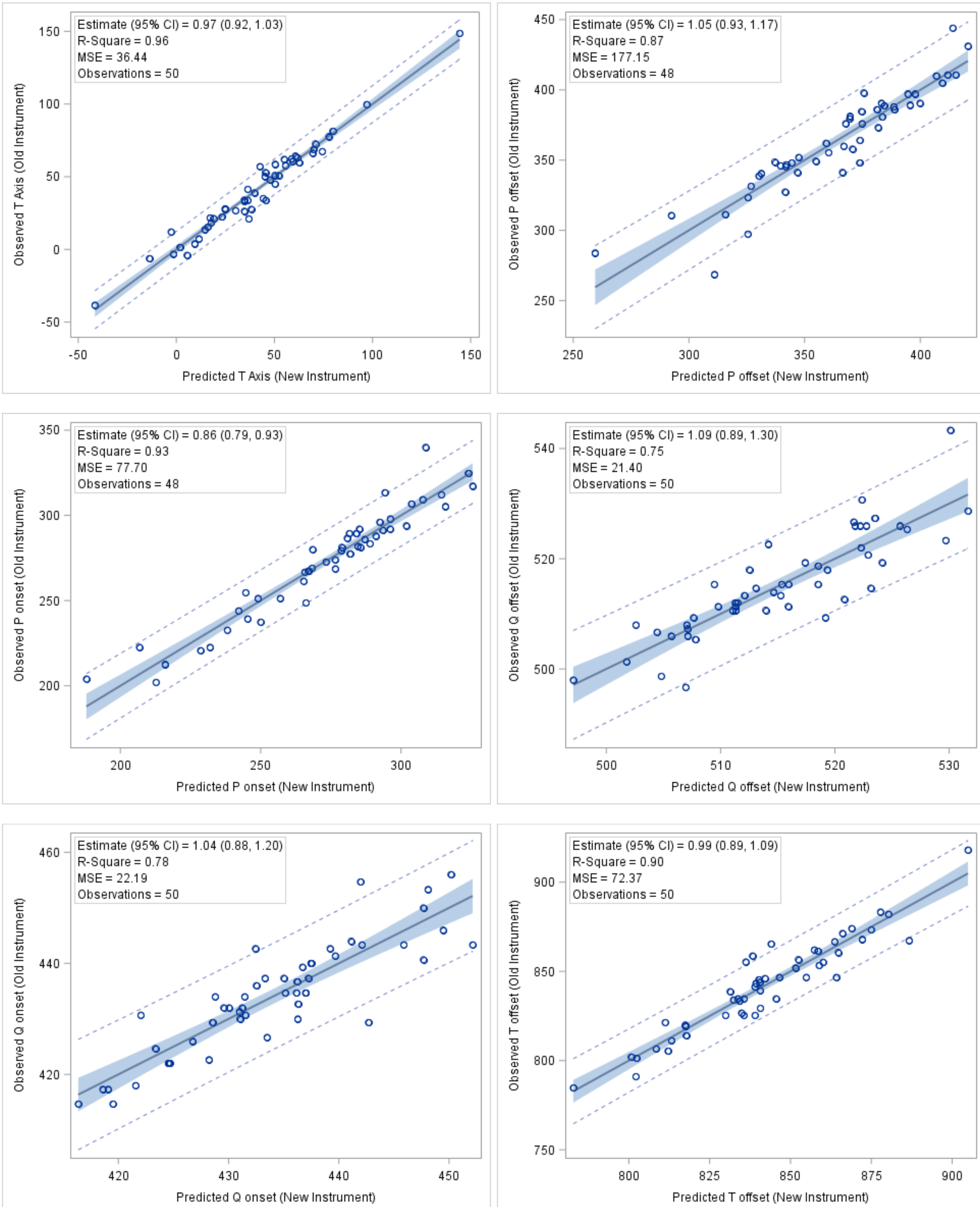

Figure S16B: Fit Plots for Cross-calibration of ECG measures

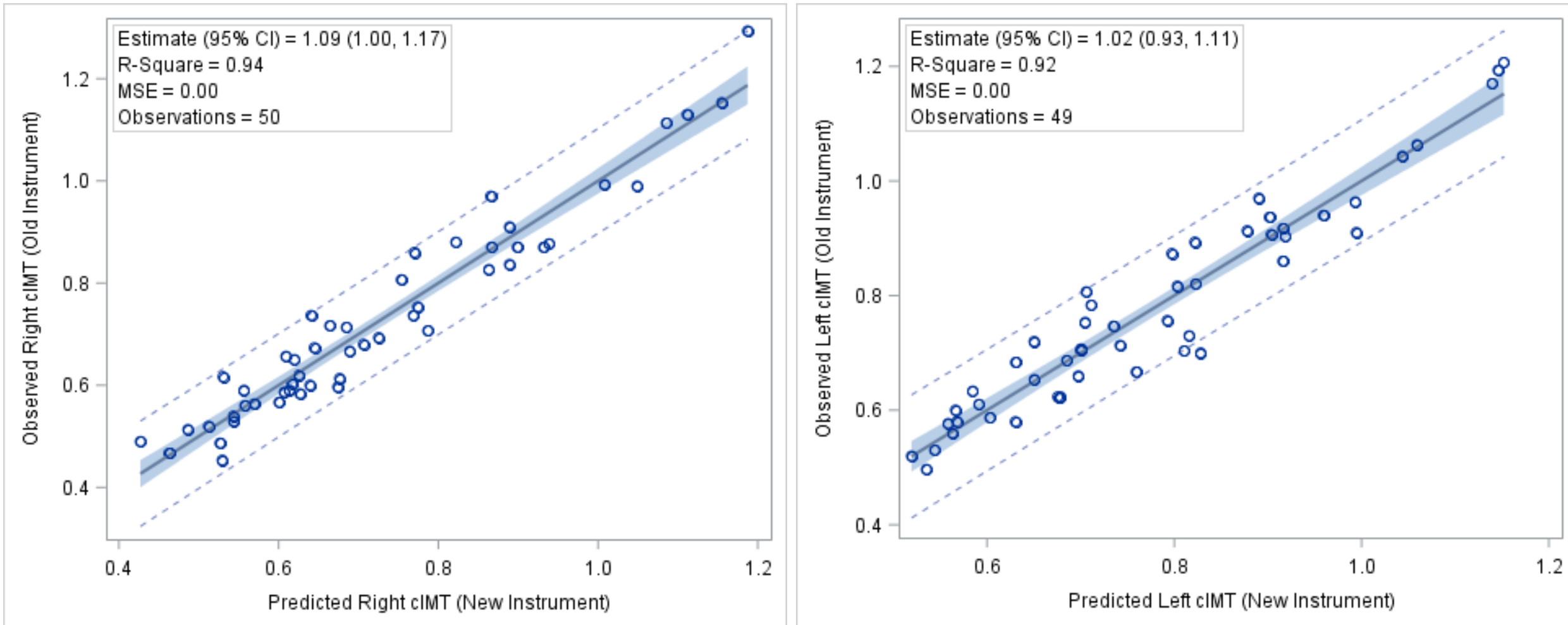

Figure S17: Fit Plots for Cross-calibration of cIMT

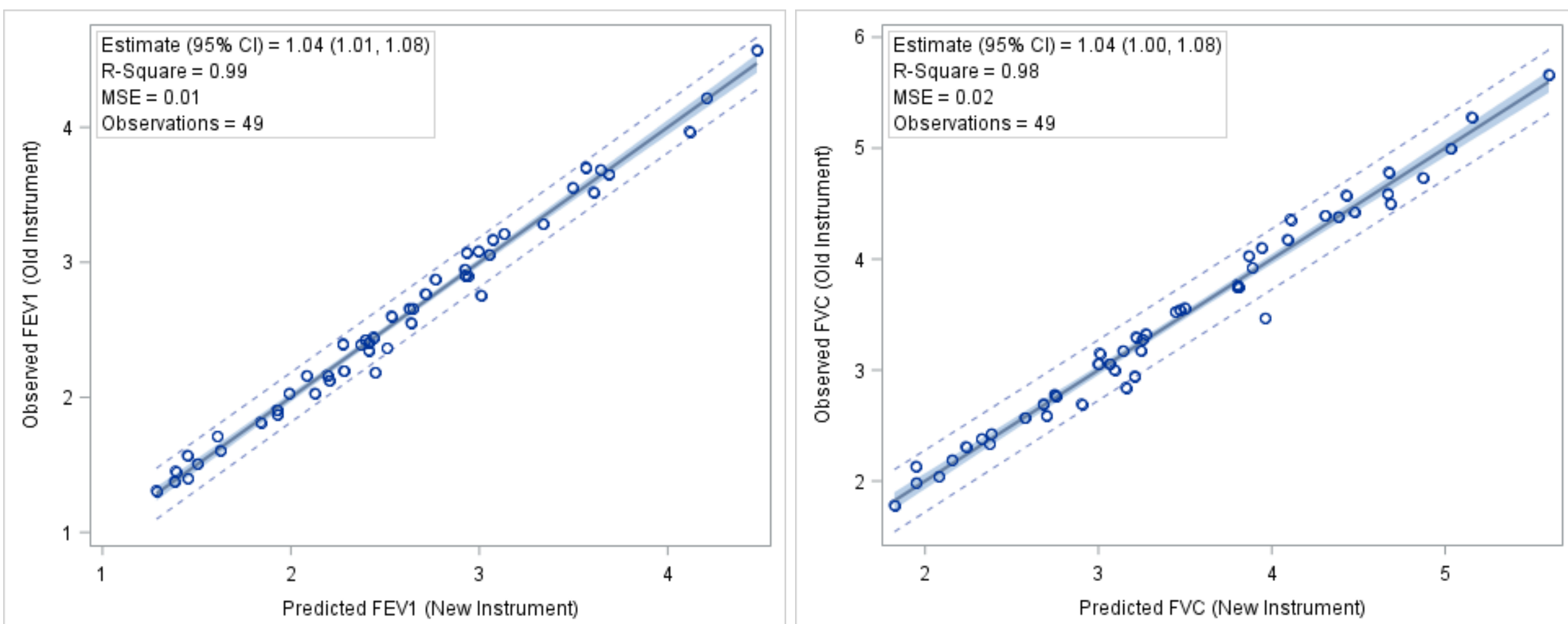

Figure S18: Fit Plots for Cross-calibration of FEV1 and FVC

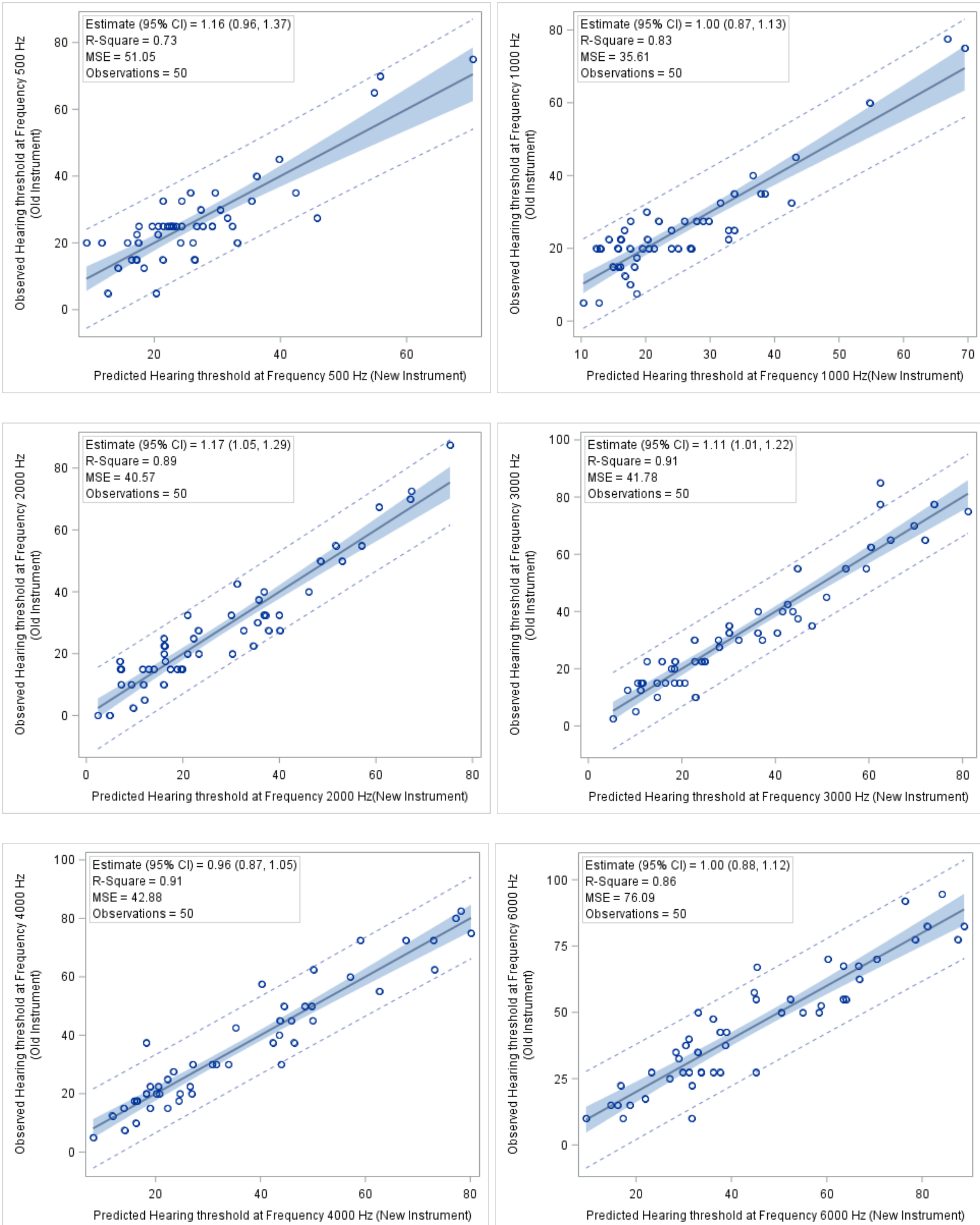

Figure S19A: Fit Plot for Cross-calibration of Hearing Assessment measures

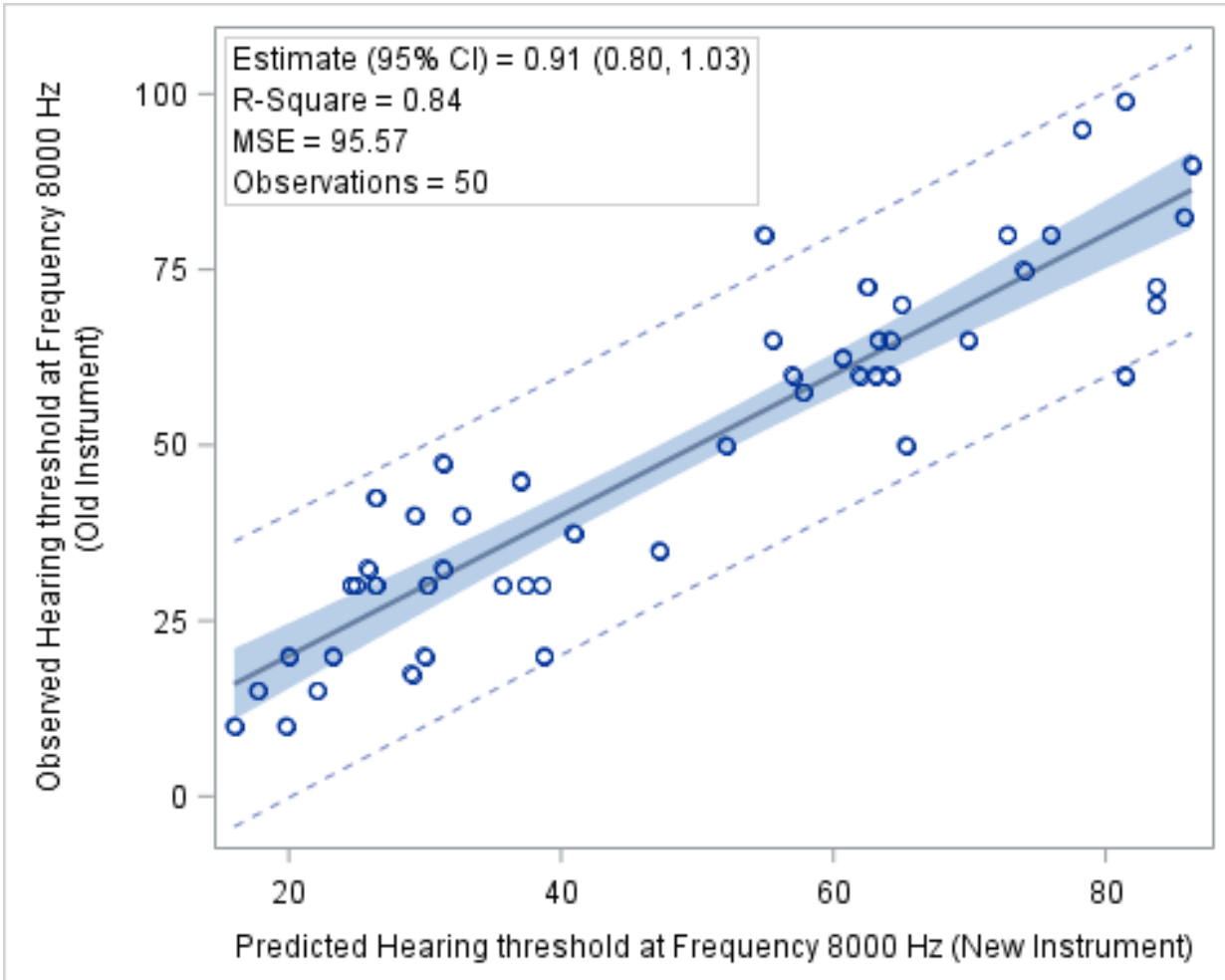

Figure S19B: Fit Plot for Cross-calibration of Hearing Assessment measures

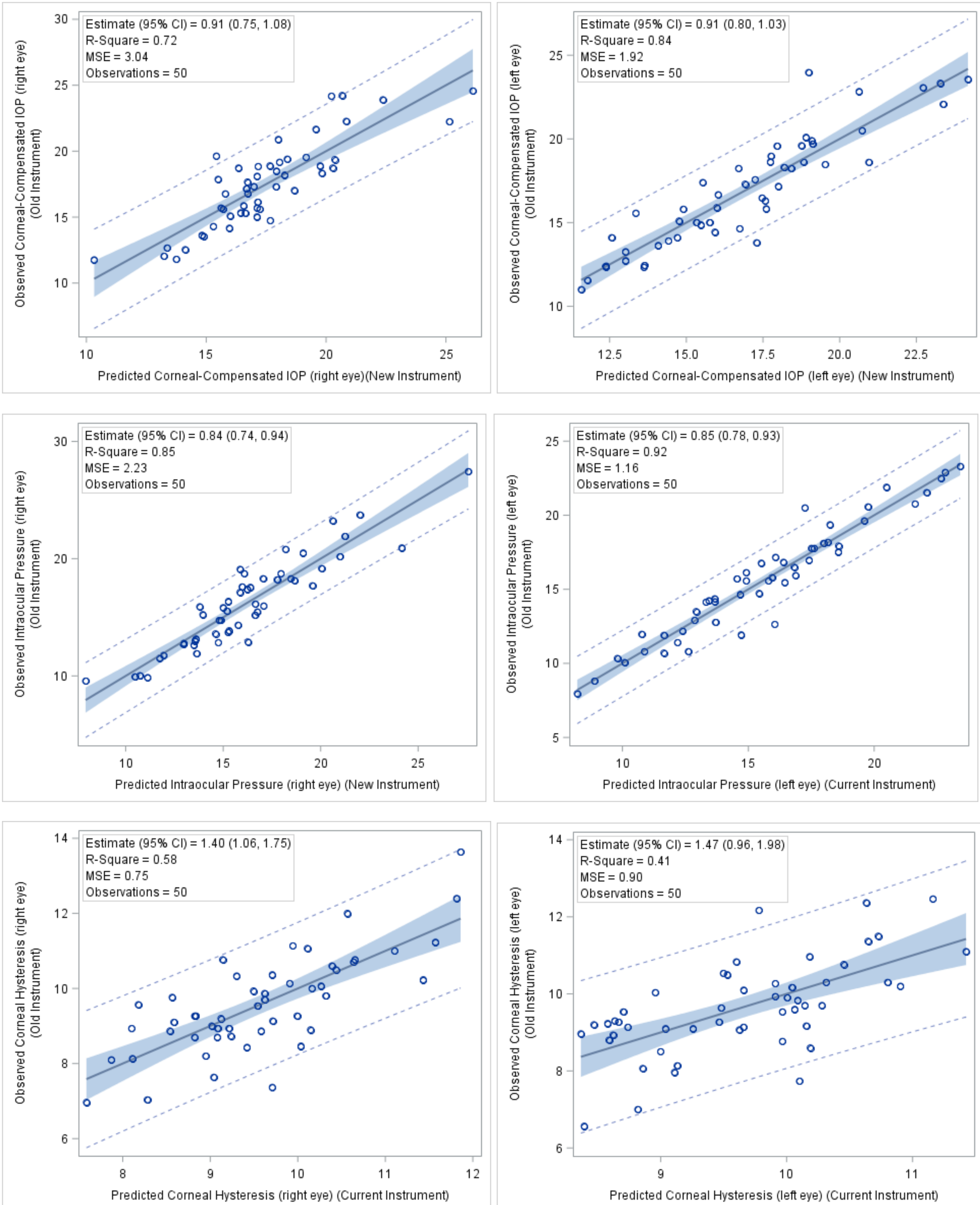

Figure S20: Fit Plot for Cross-calibration of Tonometry measures

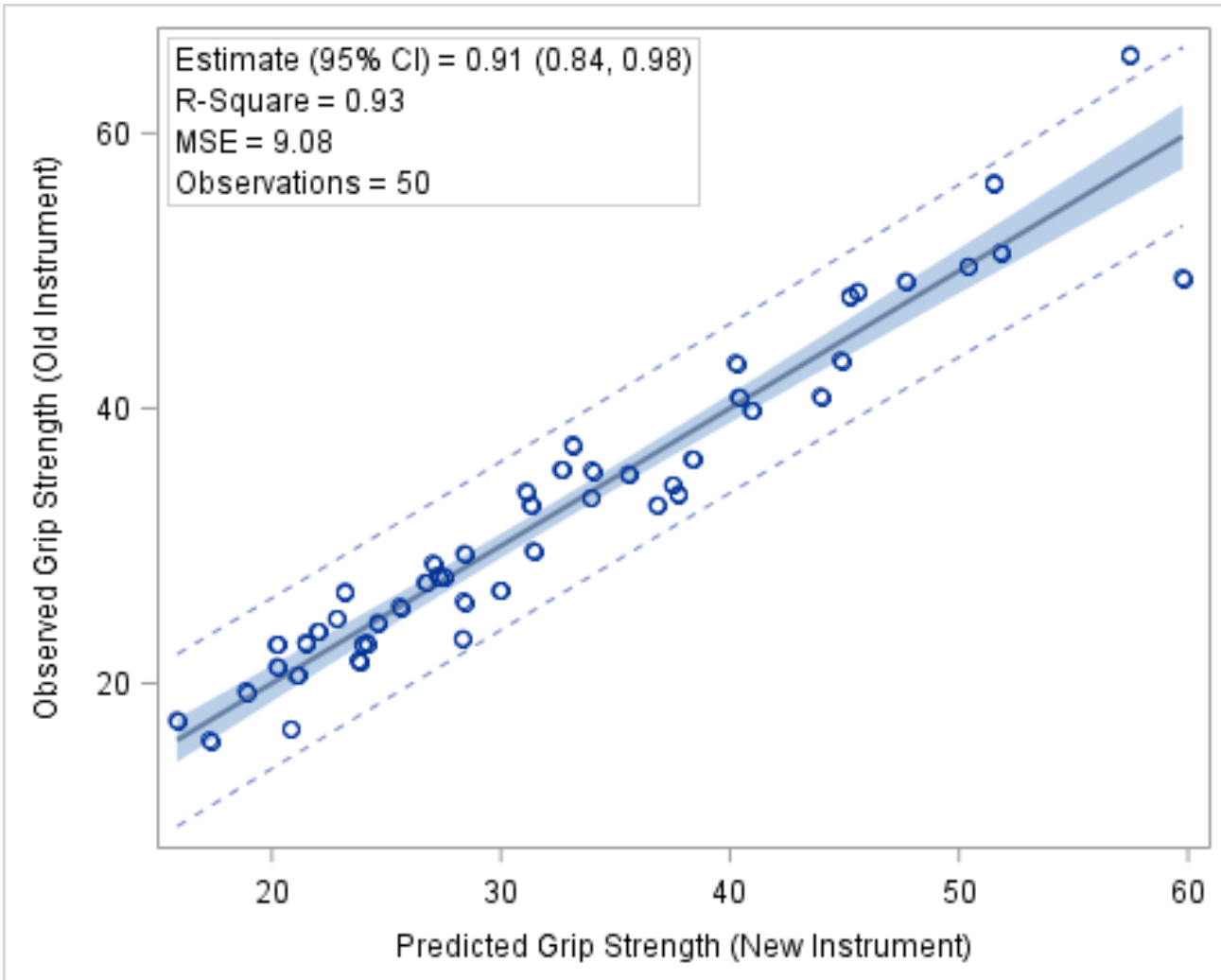

Figure S21: Fit Plot for Cross-calibration of Grip Strength

Table S17: Conversion equation for key variables between old and new instruments

	Conversion Equation
**Blood pressure monitor**	
Systolic blood pressure (mmHg)	$\hat{y}_0 = 120.94 + 0.80 \times (y_1 - 126.13)$
Diastolic blood pressure (mmHg)	$\hat{y}_0 = 67.89 + 0.77 \times (y_1 - 67.79)$
Pulse rate (bpm)	$\hat{y}_0 = 63.15 + 0.82 \times (y_1 - 61.99)$
**Spirometer (L)**	
FEV1	$\hat{y}_0 = 1.97 + 0.93 \times (y_1 - 2.02)$
FVC	$\hat{y}_0 = 2.80 + 0.92 \times (y_1 - 2.85)$
**Carotid ultrasound (mm)**	
cIMT right	$\hat{y}_0 = 0.93 + 0.89 \times (y_1 - 0.91)$
cIMT left	$\hat{y}_0 = 0.96 + 0.91 \times (y_1 - 0.94)$
**Grip strength (kg)**	$\hat{y}_0 = 17.79 + 0.91 \times (y_1 - 19.10)$
**Audiometer (dB HL)**	
Frequency 500 Hz	$\hat{y}_0 = 31.71 + 0.68 \times (y_1 - 26.36)$
Frequency 1000 Hz	$\hat{y}_0 = 30.01 + 0.77 \times (y_1 - 23.66)$
Frequency 2000 Hz	$\hat{y}_0 = 31.77 + 0.77 \times (y_1 - 26.61)$
Frequency 3000 Hz	$\hat{y}_0 = 36.17 + 0.75 \times (y_1 - 31.67)$
Frequency 4000 Hz	$\hat{y}_0 = 38.63 + 0.88 \times (y_1 - 33.03)$
Frequency 6000 Hz	$\hat{y}_0 = 48.60 + 0.77 \times (y_1 - 42.79)$
Frequency 8000 Hz	$\hat{y}_0 = 62.15 + 0.76 \times (y_1 - 56.43)$
**Tonometer (mmHg)**	
Corneal-compensated IOP - right-eye	$\hat{y}_0 = 16.87 + 0.80 \times (y_1 - 15.77)$
Corneal-compensated IOP - left-eye	$\hat{y}_0 = 16.38 + 0.86 \times (y_1 - 15.55)$
Intraocular pressure - right-eye	$\hat{y}_0 = 15.74 + 0.96 \times (y_1 - 15.37)$
Intraocular pressure - left-eye	$\hat{y}_0 = 15.41 + 1.00 \times (y_1 - 15.08)$
Corneal hysteresis - right-eye	$\hat{y}_0 = 9.66 + 0.48 \times (y_1 - 10.33)$
Corneal hysteresis - left-eye	$\hat{y}_0 = 10.04 + 0.35 \times (y_1 - 10.58)$
**Electrocardiogram (ECG)**	
Ventricular rate (bpm)	$\hat{y}_0 = 65.39 + 0.89 \times (y_1 - 65.43)$
QRS duration (ms)	$\hat{y}_0 = 78.02 + 1.03 \times (y_1 - 83.30)$
QT interval (ms)	$\hat{y}_0 = 405.50 + 0.84 \times (y_1 - 404.43)$
QTC interval (ms)	$\hat{y}_0 = 419.24 + 0.80 \times (y_1 - 417.56)$
P axis (degree)	$\hat{y}_0 = 54.61 + 0.71 \times (y_1 - 53.89)$
R axis (degree)	$\hat{y}_0 = 24.11 + 0.98 \times (y_1 - 23.68)$
T axis (degree)	$\hat{y}_0 = 61.56 + 0.99 \times (y_1 - 62.61)$
P offset (ms)	$\hat{y}_0 = 308.05 + 0.96 \times (y_1 - 319.08)$
P onset (ms)	$\hat{y}_0 = 231.93 + 1.04 \times (y_1 - 224.31)$
Q offset (ms)	$\hat{y}_0 = 514.37 + 0.70 \times (y_1 - 521.06)$
Q onset (ms)	$\hat{y}_0 = 436.33 + 0.72 \times (y_1 - 437.64)$
T offset (ms)	$\hat{y}_0 = 842.45 + 0.84 \times (y_1 - 842.99)$

References

1. Sawilowsky S. New Effect Size Rules of Thumb. *Journal of Modern Applied Statistical Methods* 2009; **8**: 597-9.
